# A critical review of the American Academy of Pediatrics technical report on abusive head trauma

**DOI:** 10.1016/j.fsisyn.2025.100650

**Published:** 2025-12-03

**Authors:** Chris Brook, Cyrille Rossant, Waney Squier, Anders Eriksson, Judy Melinek, Barry Schifrin

**Affiliations:** a Universidad de La Laguna, Spain; b Griffith University, Australia; c University College London, UK; d Previously Department of Neuropathology, John Radcliffe Hospital, Oxford, UK; e Dept. of Clinical Sciences, Forensic Medicine, Umeå University, Umeå, Sweden; f University of Otago, Wellington, New Zealand; g Chief Forensic Pathologist, Communio LTD, Auckland, New Zealand; h Western University of Health Sciences, Pomona, CA, USA

**Keywords:** Abusive head trauma, Evidence based medicine, Methodology

## Abstract

In 2025, the American Academy of Pediatrics (AAP) released a Technical Report (TR) providing guidance for diagnosing abusive head trauma (AHT). We critically examined the primary research studies cited in the TR, focusing on how cases of AHT were classified. We identified studies that sought to identify findings specific to AHT or to evaluate diagnostic accuracy, and categorized each by its methods for classifying cases as AHT. Across these studies, 71 % included cases classified by multidisciplinary team opinion, 30 % by medical records, 22 % by predetermined criteria, 22 % by caregiver confessions (only two relied exclusively on this), 9 % by court decision, and 6 % by witnessed events; totals exceed 100 % because several studies applied more than one method. We evaluated methodological rigor with particular attention to circular reasoning and incorporation bias, and found that none of these studies adequately addressed these risks. The heavy reliance on expert opinion and the systematic incorporation of prior assumptions into case classification call into question the validity of the current diagnostic framework. These findings highlight the urgent need for more rigorous, evidence-based approaches to support the validity of the AHT diagnosis.

## 1 Introduction

It is undisputed that physical assault can result in head injuries in infants. What is disputed is whether medical findings alone can reliably diagnose abusive head trauma (AHT) in particular cases. Methods used to diagnose AHT remain controversial, with ongoing critiques questioning their scientific foundation [[1], [2], [3]].

The stakes are high, and for this reason, the scientific validity of AHT diagnosis remains a matter of urgent concern. While undetected abuse may expose infants to ongoing danger, overdiagnosis creates its own cascade of harm: separation of infants from loving families precipitates measurable short-term distress [4,5] and correlates with long-term psychosocial vulnerabilities, including elevated risks of mental health disorders, cognitive deficits, and relational difficulties [6]. Moreover, erroneous diagnoses may lead to incarceration of innocent caregivers, or even a death sentence. Compounding this injustice, wrongful accusations divert attention from alternative medical explanations, leading to missed or delayed interventions and potentially exacerbating outcomes.

The recent American Academy of Pediatrics technical report [7] (AAP TR) provides a review of the literature “on various aspects of the AHT diagnosis.” While the AAP TR claims to offer “scientific information”, it explicitly acknowledges that it is not a properly conducted systematic review. The AAP TR lacks transparency in methodology, study inclusion criteria, formal quality control, and was produced without oversight by independent experts in evidence-based medicine. Instead, it states that it is “emphasizing those sources with the highest quality of evidence but not eliminating sources for which descriptive methods nonetheless provide useful and relevant information.”

This raises a critical question: how robust is the evidence base the AAP relies on to support the diagnostic methods it recommends in its TR?

The AAP TR recognises that AHT is a “complex and challenging diagnosis,” largely because there is no definitive, gold-standard test for AHT [[8], [9], [10], [11], [12]]. This not only complicates diagnosis [13,14], but also limits the ability to build reliable case samples for research —samples that are essential for drawing scientifically valid conclusions about which clinical findings support an AHT diagnosis.

To determine whether clinical findings are truly specific to AHT, especially those attributed to rotational forces (such as shaking, with or without impact), studies must include case samples where AHT has been confirmed with a high degree of certainty. Common methods for constructing samples of AHT cases include diagnosis-based categorisation (e.g., multidisciplinary team assessments), predefined diagnostic criteria, admissions or confessions, convictions, and witnessed events.

This article critically evaluates these sample creation methods and examines the references cited in the AAP TR, focusing on how each study defines its case sample and validates the recommended diagnostic methods. The frequency with which each method appears across the cited literature is quantified. Assessment is made of the risk of incorporation bias, which occurs when a diagnostic test is included in defining the group it is intended to predict. This methodological flaw is caused by circular reasoning, meaning the features used to classify cases, or related features, are then used to assess the test's accuracy.

The circular reasoning can be direct, where the test feature itself (e.g. extensive retinal hemorrhages) is a classification criterion. It can also be indirect when the classification relies on a finding that is strongly correlated with the test feature. For example, if classification relies on Subdural Hemorrhage (SDH), observing the presence of Retinal Hemorrhages (RH) in that sample only confirms the correlation between SDH and RH; it does not independently validate that RH is specific to AHT.

Circular reasoning creates artificial associations and makes the diagnostic test appear more accurate than it actually is.

Ultimately, the article aims to identify the methodologically sound scientific sources cited by the AAP TR that provide empirical support for the specificity of findings attributed to AHT, and to assess whether the diagnostic methods endorsed in the TR are supported by reliable, valid scientific evidence.

## 2 Methodology

We identified original research studies cited in the AAP TR that aim to: (1) establish which clinical findings are specific to AHT, and/or (2) provide evidence for the accuracy and validity of diagnostic methods used to identify AHT.

While it is widely accepted that AHT can cause bruising and skull fractures (as can accidental trauma), studies related to these findings were only included when a direct connection to AHT is made. For instance, studies that assess the presence of bruising in diagnosed AHT cases are included; studies focusing solely on general bruising patterns, without reference to AHT, are excluded. Similarly, cases involving skull fractures are included only when the study draws conclusions about the diagnostic relevance of such findings in the context of AHT.

It is also widely accepted that natural causes can result in clinical findings such as intracranial hemorrhages [[15], [16], [17], [18]], retinal hemorrhages [[19], [20], [21], [22]], hypoxic brain injury [[23], [24], [25]], apnea [26,27], seizures [28], vomiting, poor feeding, and irritability. Our study does not attempt to evaluate the evidence supporting specific alternative explanations for these findings. Nor does it independently assess the validity of evidence used to rule out particular alternative causes. Consequently, studies that explore alternative mechanisms (e.g., coagulopathies) without direct comparison to AHT are excluded. However, because consideration of differential diagnoses is an integral part of the diagnostic process for AHT that we are appraising, the differential diagnosis process is inherently included in our assessment of the accuracy and methodological rigor of AHT diagnostic methods.

In stage one, two reviewers categorized the articles’ reference type and subject using the following options:

A. Reference Type:
  Primary Research Study (Original research e.g., clinical trials, cohort studies)
  Case Report (report on one or a few patients or cases)
  Book/Book Chapter
  Systematic Review
  Meta-Analysis
  Narrative Review/Opinion Piece (qualitative summary w/o systematic methodology)
  Other. Please describe:
  Unsure
B. Subject of Study:
  Associated findings and diagnostic methods for SBS and/or AHT^1^
  Biomechanics (e.g., animal studies, crash test dummy simulations)
  Incidence rates of AHT
  Outcomes and Long-Term Effects of AHT
  Legal and Forensic Aspects (e.g., court case, forensic pathology, expert reliability)
  Perpetrator characteristics, Risk Factors and Prevention Strategies
  Race bias
  Other. Please describe:

Articles classified by two reviewers as primary research on AHT-associated findings or diagnostic methods were included; those classified by a single reviewer were reviewed by a third. Of the 683 articles cited in the AAP TR, 149 were deemed by two reviewers to meet these criteria.

These 149 articles were then reviewed to determine what method was used to classify cases as AHT. Six methods were identified. For each study, the method used to classify AHT was selected from the list of six, and assessed for risk of circularity and incorporation bias.

Method used to classify as AHT:
  Diagnosis by Multidisciplinary Teams (MDTs) or experts
  Diagnostic code/Medical records
  Predetermined Criteria
  Admission or Confession
  Conviction or court confirmation
  Witnessed
  Other classification method. Please specify:
Comment on Methodology:

Describe how the study creates a sample of AHT cases:

E. Has the study adequately addressed the risk of incorporation bias and/or circular reasoning?
  Yes
  No

## 3 Results

We found that 107 studies (71 %) used categorisation by multidisciplinary team or expert opinion, 43 (30 %) used diagnostic codes or medical records, 33 (22 %) used predetermined criteria, 32 (22 %) used confessed cases, (only 2 used them exclusively. The confessed cases were typically a small fraction of cases), conviction or court decision was used in 14 studies (9 %). Witnessed cases were used in 9 studies (6 %), but only comprised a small fraction of cases. Five studies provided no method, simply analysing cases said to be AHT.

**Fig. 1:**
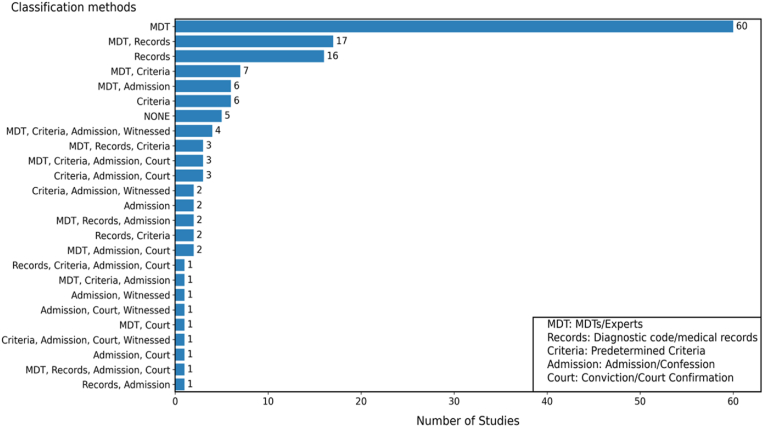
Classification methods, including combinations of methods, used in the 149 primary research studies cited in the AAP TR that relate to findings specific to and diagnostic methods for AHT. 5 of the studies provided no methodology.

The sum exceeds 100 % as some studies categorized cases by multiple methods (see Fig. 1). In supplementary material, we provide the full set of survey responses.

In three appendices, detailed analysis is provided of the methodology of articles cited in the AAP TR to support their assertions in three particularly important topics:

● Appendix 1: evidence for an association between subdural hemorrhage (SDH) and AHT, and for the claim that SDH is significantly more common in abusive than accidental head trauma.
● Appendix 2: evidence for the association of ocular findings with AHT.
● Appendix 3: evidence for the accuracy of diagnostic methods including Clinical Prediction Rules and Pooled Analyses.

These appendices include analysis of systematic reviews cited in support of assertions made regarding these topics. In Appendix 4, systematic reviews that were not already analysed in the first 3 appendices, and that are relevant to findings associated with AHT and diagnostic methods, are analysed.

## 4 Discussion

Methods of Categorisation.

### 4.1 Categorisation by multi-disciplinary teams

The most common method involves multidisciplinary teams (MDTs) and/or experts classifying cases as abusive or non-abusive. This approach has fundamental weaknesses.

Firstly, MDT classification identifies *who* made the determination but not how. It fails to disclose the actual evidence or reasoning behind the determination. This reliance on expert opinion without transparent justification reflects authoritative medicine, the antithesis of evidence-based medicine (EBM). Without a clear presentation of the evidence used to support an AHT classification, assessing the reliability and objectivity of these determinations becomes impossible, undermining scientific rigor and EBM principles.

Further, MDT-based diagnoses are prone to circular reasoning and incorporation bias. Child protection pediatricians associate certain medical findings with AHT, and MDTs (or ‘experts’) use these same findings to classify cases as AHT. Studies using such classifications then treat these findings as indicators of AHT, creating a self-reinforcing loop rather than providing independent validation of diagnostic criteria.

Including non-medical MDT members, such as police officers or social workers, does not break this circularity. Research shows that they defer to clinicians when making decisions [29] so classification remains overwhelmingly dependent on the medical interpretations of child protection pediatricians. If non-medical evidence is crucial for classifying a case as AHT, researchers must specify that evidence rather than merely noting non-medical involvement in the MDT.

In medicine, MDTs do get used in the presence of diagnostic uncertainty or multiple possible treatments, and their decisions are tested by patient outcomes. This feedback loop is central to clinical practice. By contrast, AHT MDTs do not treat patients and do not receive feedback, meaning that the accuracy of this diagnostic method cannot be determined. Even if every diagnosis were wrong, they would have no way to know, yet still accrue ‘experience’ in the diagnosis of AHT.

The AAP TR [30] claims that MDTs reduce cognitive bias and subjectivity diagnoses, but provide no evidence for this. Whilst *independent* experts and *independent* evidence may reduce the effects of cognitive bias [31], this does not apply when there is interaction and discussion between the different experts, with evidence assessed within the contextual information provided by the other experts, creating conditions for groupthink and the reinforcement of shared assumptions.

Indeed, the use of MDTs may *increase* the risk of bias, because “contextual information can have such a strong and biasing effect to a level that it may not only determine the decisions … it can even override the evidence-based decision, so the decision is modified to fit the contextual information” [32] This is referred to as the “biasing snowball effect” [33]. The use of MDTs incorporates the biasing snowball effect into the diagnostic process, contradicting recommendations for reducing cognitive bias within forensics [34], which instead highlight the importance of *compartmentalization* and the *blinding* of experts to task-irrelevant contextual information.

Regardless, the fact that MDTs are used in diagnosis more broadly in medicine, or the possibility that they may reduce bias, does not justify their use as a scientific reference standard. The two functions are separate, and it is important not to conflate them. MDT diagnoses cannot be used as a reference standard to determine the diagnostic methods used by MDTs to make diagnoses.

What studies relying on MDT classification *can* do is identify which findings are commonly used by experts and MDTs in making an AHT diagnosis. What they cannot do is verify whether those findings are being appropriately used for diagnostic purposes, nor whether the diagnoses are accurate.

A related approach in retrospective studies involves categorizing cases based on diagnosis codes. This method inherently assumes that the initial diagnosis was correct. Much like MDT-based classification, diagnosis-code-based studies do not assess whether these diagnoses were accurate. Instead, they merely identify findings that have been historically associated with AHT, reinforcing existing assumptions rather than validating them.

None of these studies can be used to identify findings associated with AHT, nor can they provide an evidence base for validating the diagnostic methods used for AHT.

### 4.2 Categorisation by predetermined diagnostic criteria

Classifying cases based on predefined criteria also has fundamental weaknesses.

First, none of these criteria have been independently validated as objective indicators of AHT and therefore cannot reliably be used to create a true sample of AHT cases.

Second, these studies invariably include criteria that are subjective, relying on preconceived assumptions held by experts and multidisciplinary teams about how AHT should be diagnosed. For example, the Bechtel et al. [35] sample is dominated by cases classified as AHT due to having “no history of traumatic event.” This is not an objective indicator of AHT: many infants who have not been subjected to abuse present with no history of a traumatic event. What this criterion effectively means is that the infant exhibited findings associated with AHT, was assessed by child protection paediatricians as suspicious for AHT based on those findings, and the caregiver provided no history of a traumatic event.

Simply stating “no history of a traumatic event” or that the caregiver “denied” head trauma [36], without describing how this is integrated with clinical findings, leaves the classification opaque and unscientific. As a result, the problem of circular reasoning and incorporation bias persists.

Indeed, Vinchon [37] states that they “have proposed since 2004 a four-tier grading based on easily identified features [38,39], however, it was difficult to validate the diagnostic value of this grading because of the circularity bias”, acknowledging that the use of predefined criteria leads to circularity and incorporation bias.

Similarly, Piteau et al. [40] stated that “as there are no standardized criteria for the definition of abuse, most authors developed their own criteria, and many of these are fraught with circular reasoning”. They used a previously published [41] 5-point scale to define abuse but accepted that it “does not compensate well for circularity” for features that have been “traditionally associated with abuse such as subdural hemorrhage and retinal hemorrhage”.

Some studies also include confessed cases as part of their classification criteria. While these typically represent a statistically insignificant subset (with a couple of exceptions), their use introduces additional problems, including circular reasoning and incorporation bias, as discussed below.

Some predetermined criteria are objective, such as independently witnessed cases of AHT. However, there are invariably only a very small number of such cases, making them statistically insignificant, so the existence of this criterion does not save the studies from the subjective, unvalidated criteria that dominate the samples.

Another issue with these unvalidated predetermined criteria for classifying cases as AHT is the correlation between findings associated with AHT and those linked to the most severe intracranial pathologies [[42], [43], [44]]. For example, unsupervised clustering techniques have identified two distinct clusters of cases with intracranial pathology: one characterized by findings purportedly associated with AHT, and the other by findings not associated with AHT [45]. The most serious cases, those involving loss of consciousness for 24 h or more after admission with clinical deterioration, have an odds ratio of 335 (95 % CI: 46–2441) of falling within the AHT-associated cluster. As a result, any classification system that selects for more serious cases will, by design, also select for findings purportedly associated with AHT, without independently establishing that those cases actually involve AHT, or that those findings are actually associated with AHT.

What classification using predetermined criteria *can* show is correlations between findings. For example, Duhaime et al., 1992 [46] include intradural hemorrhages (IDH) in their algorithm for classifying cases as AHT. This is a clear example of circular reasoning and incorporation bias. Because IDH formed part of the case definition, its association with AHT cannot be considered an independent finding: the association between IDH and AHT is an input of the study, not an output. However, the observation that cases classified with IDH tend to also have extensive retinal hemorrhages (RH) does support a correlation between IDH and RH but it does not establish an objective link between RH and AHT.

In summary, almost a quarter of studies used categorisation by predetermined diagnostic criteria that themselves have never been validated, and that include subjective criteria that risk circularity and incorporation bias.

### 4.3 Categorisation by confessions

Several proponents of orthodox approaches to diagnosing AHT maintain that confession studies constitute the central evidentiary basis for inferring the application of significant rotational forces (such as through violent shaking) from particular medical findings [[47], [48], [49]]. This is a concession of what we have outlined, i.e. the above methods for classification are flawed due the use of poor quality, subjective reference standards and the associated risk of circular reasoning and incorporation bias.

The idea of classification by confessions was to break this circularity. Vinchon claims that “basing the diagnosis of IHI [inflicted head injury] on confession rather than medical features, eliminates this bias …” [50].

However, almost all confessions to AHT in the literature have been made *after* medical evaluation has identified findings associated with AHT, creating a selection bias for including only such cases, and thus failing to avoid circular reasoning and incorporation bias [51]. Cases without AHT-associated findings are not reported to child protection teams, are not investigated, and caregivers are not questioned.

This explains why the confession based study of Vinchon [52] found 100 % specificity of the “Ontario triad” for AHT. A result of 100 % specificity is a red flag for circular reasoning, particularly in a field where diagnosis is complex, and overlaps with natural causes. The author said that “[w]e admit that we were a bit disturbed to find a 100 % positive predictive value for the association of severe RH with subdural hematoma (SDH) and absence of signs of impact, because this figure does not look like a scientific result; however, from a legal perspective, we think that this is precisely what a judge hopes for” [53].

In the small number of cases where confessions to shaking were made *before* medical evaluation, the infants did not exhibit significant intracranial pathologies [54].

A failure to address circularity and incorporation bias is not the only flaw in confession-based studies. It is now well-established that people sometimes confess to crimes they did not commit, even to the most serious crimes [55]. Moreover, extensive research has identified the conditions under which false confessions are most likely to occur—conditions that are inherently present in AHT cases. Key risk factors include interrogators who are already convinced a crime has occurred (often based on medical findings), coercive or suggestive questioning of vulnerable caregivers, plea bargains, hope for reduced sentences, the prospect of having children returned home, financial pressure, and a desire to protect a partner or bring an end to prolonged legal proceedings [[56], [57], [58], [59]].

Confessions also formed the central evidentiary basis for the existence of witches. Yes, many confessions to witchcraft were attained through torture, but many others were not. In numerous historical cases, individuals accused of witchcraft confessed under immense social pressure—facing stigma, religious condemnation, community fear, and the threat of ostracism or execution. Some confessed out of a desperate hope for leniency, others to protect loved ones, and some after isolation or psychological coercion [60].

Just as confessions to witchcraft do not constitute a scientific evidence base for the existence of witches, confessions to AHT do not provide valid scientific support for the diagnostic methods used in AHT.

In most studies, confessed cases were few and stastically insignificant. Two studies used only confessed cases, but they were made after medical evaluation had led to investigation, resulting in risk of selection bias and circularity. Circumstances such as whether they involved child removal, the nature of the investigative procedures, any judicial, sociological and/or societal pressure being felt by the accused or whether the accused was the one who suggested the mechanism of abuse, were not provided in any study.

A systematic review [61] of confessed cases of AHT, which is cited in the AAP TR, is analysed in Appendix 4.

### 4.4 Conviction or court confirmation

Convictions for AHT are typically based on medical findings that are believed to indicate abuse. As such, cases selected on the basis of conviction will, by definition, display those same findings. This creates a high risk of circular reasoning and incorporation bias: the presence of certain findings leads to a diagnosis of AHT, leading to conviction, and that conviction is then used to confirm that those findings are indicative of abuse.

### 4.5 Categorisation by witnessed events

Witnessed accounts provide another way to categorize cases as AHT, as they are assumed to offer independent confirmation of abuse. Some cases are even caught on video. Studies that have exclusively used witnessed shaking events as a reference standard have found that “few witnessed shaken infants have signs and symptoms of AHT” [62], contradicting results from the methods described above, and even those few cases lacked *independent* witnesses.

For a witnessed account to be considered independent and unbiased, it should come from someone unrelated to the infant or accused and be made prior to any medical evaluation, suspicion of abuse, or accusation. There are several reasons for this:

● When both parents are under suspicion, one may accuse the other to deflect blame.
● After being told by medical professionals that the infant was physically abused, a caregiver who knows they did not do it may infer that their partner must have, providing motive to accuse the other caregiver, not wanting them to get away with it.
● Child removal creates intense emotional pressure. In some cases, one caregiver may incriminate another in hopes of regaining custody or reducing their own legal jeopardy.
● One parent may falsely confess to deflect blame from the other, allowing their partner to regain custody of their children [63,64].

For these reasons, only pre-medical, spontaneous witness statements or third-party, independent eyewitness accounts—preferably corroborated by more than one witness—can be considered sufficiently unbiased to form a reliable sample of AHT cases suitable for identifying associated findings.

Witness statements made after medical suspicion has arisen and accusations have been made are subject to the same circularity and selection bias that undermine the evidentiary value of confessions following accusations based on particular medical findings.

Requiring independent witnesses to AHT mirrors the requirement for independent witnesses to verify accidental trauma, a common reference standard [[65], [66], [67]]. The same standards should apply when classifying cases as AHT or as accidental. No independently witnessed or videotaped shaking of a healthy infant, of which there are numerous cases, has ever resulted in the clinical findings associated with AHT [68].

## 5 Limitations

This review is restricted to the studies cited in the 2025 AAP Technical Report and therefore does not represent an exhaustive survey of all literature related to AHT. However, the AAP describes these studies as the “best available” in the field. Our focus was on methodological rigor, particularly the risks of circular reasoning and incorporation bias, and we did not attempt to evaluate the strength of evidence for other potential medical explanations of the findings.

## 6 Conclusions

We agree with the AAP TR that “no evidence-based guidelines for the management of the child with AHT currently exist.” The AAP TR does not fill this gap, nor does it claim to. Our analysis explicitly demonstrates the limitations of the evidence base, as outlined in the AAP TR, underpinning current diagnostic practice in suspected AHT.

The AAP TR does not provide specific diagnostic criteria, diagnostic thresholds, or a standardized diagnostic framework. While a series of findings are described as suggestive of, or suspicious for, AHT, none are diagnostic. It instead states that “throughout the diagnostic differentiation process, the pediatric provider will have to manage various uncertainties until the individual threshold of diagnostic sufficiency is reached,” thereby relying on subjective judgments.

Our assessment of the diagnosis of AHT encompasses the entire process, specifically including the differential diagnosis. This process of excluding other differential diagnoses requires further subjective judgments regarding their degree of likelihood. The diagnosis by exclusion process is particularly troubling given that approximately half of all infant deaths are attributed to rare conditions [69], including newly discovered genetic disorders [70], while 5–10 percent have causes that remain undetermined [[71], [72], [73]]. In such a context, diagnosis by exclusion is fraught with uncertainty, compounded by the complexity of AHT diagnostic methods, the weak evidence base underpinning those methods, and the broader problem of misdiagnosis in medicine [74].

Rather than provide clear diagnostic guidelines, the AAP TR emphasizes “the value of a multidisciplinary team approach to the evaluation and treatment of the child with AHT. Essential team members include the trauma surgeon, neurosurgeon, pediatrician (ideally, a child abuse pediatrician), and a social worker …” In the absence of specific diagnostic criteria or thresholds, this reliance on expert clinical judgment and multidisciplinary consensus highlights the continued dependence of the field on authoritative medicine rather than objective, evidence-based diagnostic standards.

## 7 Summary

A core feature of rigorous scientific methodology is the use of independent measures and reference standards, thereby avoiding circular reasoning and incorporation bias. The AAP TR identified no studies that purported to determine which findings are associated with AHT, or to determine diagnostic accuracy, and that had adequately addressed the risk of circular reasoning and incorporation bias. Our analysis indicates that the classification of cases as AHT is primarily based on expert or multidisciplinary team determinations, often guided by unvalidated predetermined criteria, and that the findings leading to these determinations are subsequently cited as diagnostic features of AHT.

While we acknowledge the clinical experience of practitioners in this field, expert or clinical opinion is routinely classified as the lowest tier of evidence within evidence-based medicine hierarchies [75,76]. Diagnoses based on expert consensus do not meet the standards of scientific validation. Accordingly, although the AAP TR reflects clinical perspectives and the ‘best available evidence,’ it should not be presented as validated science. Clear differentiation between clinical consensus and validated science is essential to ensure transparency, integrity, and public trust in medical guidance.

As Ioannidis [77] warned, “claimed research findings may often be simply accurate measures of the prevailing bias.” In the case of AHT research, circular reasoning and incorporation bias are not incidental flaws but systematic features of the methodology of the field. They serve as mechanisms for transforming prevailing opinion into seemingly empirical findings. Given the extensive, essentially exclusive reliance on circular reasoning and incorporation bias in the research cited by the AAP TR, there are clear indications that prevailing bias is the very foundation of the evidentiary framework supporting AHT diagnostic methods.

## CRediT authorship contribution statement

**Chris Brook:** Writing – original draft, Project administration, Methodology, Formal analysis. **Cyrille Rossant:** Writing – review & editing, Project administration, Conceptualization. **Waney Squier:** Writing – review & editing, Formal analysis, Conceptualization. **Anders Eriksson:** Writing – review & editing, Formal analysis. **Judy Melinek:** Writing – review & editing, Formal analysis. **Barry Schifrin:** Formal analysis, Writing – review & editing.

## Clinical trial registration

N/A.

## Funding/support

No funding was secured for this study.

## Declaration of competing interest

The authors declare the following financial interests/personal relationships which may be considered as potential competing interests:

Waney Squier, Anders Eriksson, and Judy Melinek have been consulted by and testified regarding child abuse cases (including AHT cases), for both prosecution and defense. Barry Schifrin has testified regarding child abuse cases (including AHT cases) for the defense. Cyrille Rossant serves as president of L’association Adikia, which provides families facing contested medical determinations of child abuse with access to support groups to (unpaid activity). Chris Brook has no interest competing to declare.

## Footnotes

1 For associated findings and diagnostic methods for SBS/AHT, researchers were guided by the following considerations: Does the study aim to determine which findings are associated with AHT/SBS? Does the study aim to compare rates of certain findings in cases of AHT/SBS to other causes such as accidents? Does the study aim to determine the accuracy of the diagnostic methods for AHT/SBS, including the complex differential diagnosis?.
