# Supplementary material for: A critical review of the American Academy of Pediatrics technical report on abusive head trauma
Source: Forensic Sci Int Synerg. 2025 Dec 3;11:100650. doi: 10.1016/j.fsisyn.2025.100650 (PMC12721060; doi:10.1016/j.fsisyn.2025.100650)
Supplement: Multimedia component 1 [file mmc1.docx]

**A Critical Review of the American Academy of Pediatrics Technical Report on Abusive Head Trauma**

**Appendix 1**

**Subdural Hemorrhage (SDH)**

In this appendix, we analyze citations in the section *Physical Findings c) Intracranial Hemorrhage/Hematoma* that purport to provide evidence for a close association between subdural hemorrhage (SDH) and abusive head trauma (AHT), and for the claim that SDH is significantly more common in abusive than accidental head trauma.

- The AAP TR claims that SDH is “the most common intracranial manifestation of AHT.”

This claim is supported by four citations^^[[1]](#endnote-2)^,^[[2]](#endnote-3)^,^[[3]](#endnote-4)^,^[[4]](#endnote-5)^^. The first, Kelly et al. 2015¹, describes a multidisciplinary diagnostic process involving a consultant pediatrician and a hospital social worker, who took a history and discussed the case with the primary team, including differential diagnosis. The MDT based their AHT diagnosis on either “a history of assault (and injuries compatible with that history) or injuries regarded as not compatible with the history provided by the end of the [medical and investigative] process described above.” In other words, the MDT examined a set of undisclosed findings and, applying prior assumptions about what could cause those findings, determined them to be compatible or incompatible with the infant’s history. No mention is made as to whether SDH was a factor in the MDT’s diagnosis, but SDH was widely considered closely associated with AHT at the time. There is therefore a high risk of incorporation bias and circular reasoning. The methodology relies on presumed associations between SDH and AHT, rather than establishing those associations independently.

The second, Bradford et al. 2013^2^ and third, Dias et al. 1998^3^, take AHT cases from medical records. While they provide no information on how AHT was diagnosed, it is highly likely that the presence of SDH influenced the diagnosis in many cases. Both articles carry a high risk of incorporation bias and circular reasoning

In the fourth, Matschke et al. 2009^4^, AHT was identified by confession, criminal conviction, or a unanimous conclusion of all authors based on the presence of at least three of the following criteria: signs of serious external injury (hematomas or lacerations), unexplained fractures (of long bones, ribs, or skull), SDH, traumatic intracerebral pathology (e.g., simple or gliding contusions), retinal hemorrhage, and/or a clearly inadequate history given the clinical findings. So SDH was explicitly included as one of the diagnostic criteria. The authors do not specify how many cases involved SDH as part of the diagnosis, nor how many cases were classified as AHT due to confession or conviction. They also provide no information on whether SDH contributed to making the accusation that preceded a confession, or to the medical opinion that led to conviction. The study demonstrates explicit incorporation bias by including SDH in the diagnostic process, and a high risk of additional, hidden incorporation bias. It carries a high risk of circular reasoning and does not establish the claimed close association between SDH and AHT.

- The AAP TR then claims that SDH “more commonly occurs following abusive rather than accidental head injury”, citing two reviews^^[[5]](#endnote-6)^,^[[6]](#endnote-7)^^ and eight primary articles.^^[[7]](#endnote-8)^,^[[8]](#endnote-9)^,^[[9]](#endnote-10)^,^[[10]](#endnote-11)^,^[[11]](#endnote-12)^,^[[12]](#endnote-13)^,^[[13]](#endnote-14)^,^[[14]](#endnote-15)^^

**The reviews**

The first review, Piteau et al, 2012^5^, outlines its inclusion criteria in Table 1, which required that studies use one of two highest levels of AHT classification. Level 1 was “abuse confirmed at case conference or civil, family, or criminal court proceedings, or admitted by perpetrator.” Given that SDH was widely regarded as indicative of AHT at the time, it is highly likely that SDH contributed to the confirmation of abuse in case conferences, the findings of abuse in court, and the accusations that led to confessions. The review did not require any information about the circumstances or contents of confessions. Level 2 was defined as “abuse confirmed by stated criteria including multidisciplinary assessment,” but there was no requirement that these criteria be validated, nor was there any information on how often SDH featured in the diagnostic process. As a result, this review includes studies with methodologies at high risk of incorporation bias and circular reasoning.

Of the 24 studies included in the review, 17 were also cited in the AAP Technical Report and have been examined in the main body of our article: *x* used multidisciplinary team (MDT) classification, *y* relied on confessions, and *z* used other criteria [specify]. Of the remaining seven studies:

- In one, Vinchon et al. 2005^^[[15]](#endnote-16)^^, the study’s own lead author explicitly acknowledged it suffered from “circularity bias,” ^^[[16]](#endnote-17)^^ yet was nevertheless rated as high quality (Level 1) by the review, based on the criteria used to determine the etiology of head injury.
- Two additional studies, Kelly & Hayes 2004^^[[17]](#endnote-18)^^ and Ruppel et al. 2001^^[[18]](#endnote-19)^^, were rated as low quality by the review itself, again based on their criteria for determining etiology.
- This means 20 of the 24 studies have been assessed as having poor methodology, leaving 4 studies^^[[19]](#endnote-20)^,^[[20]](#endnote-21)^,^[[21]](#endnote-22)^,^[[22]](#endnote-23)^^ which have not yet been assessed .

The first of these remaining four, Billmire & Myers 1985^19^, classified cases as AHT based on one or more of the following: a history or confession of abuse (10 cases), the presence of multiple injuries (12 cases), or an inadequate explanation for the infant’s injuries (6 cases). Among the 12 cases with “intracranial injury only,” 11 were classified as abuse. Notably, in at least 5 of these 6 cases, the explanation for the intracranial findings was deemed inadequate, and this led to the diagnosis of AHT. This represents explicit incorporation bias in roughly half of the “intracranial injury only” cases. Although it is not explicitly stated whether intracranial injury itself factored into the other diagnostic criteria used in these cases, it is highly likely, introducing a further risk of hidden incorporation bias.

The second, Tzioumi & Oates 1998^20^ relied on the diagnosis of AHT made by the child protection team, without providing any details on the diagnostic process, essentially a black-box authoritative categorization. Despite this, the review rated it as employing “high quality criteria” for determining the etiology of the head injuries.

The third, Hobbs 1984^21^, based AHT classification on “the history and clinical findings”. In 27 of 29 cases this was “assisted by the presence of multiple injuries characteristic of abuse”. In “at least 10” of those cases, the caregiver *later* confessed to police. Thus, AHT cases were identified—including confessed cases—because they had findings characteristic of abuse, and those same cases were then used to determine which findings are characteristic of abuse.

The fourth, Vavilala et al. 2007^22^, does not specify how AHT cases were classified, yet the review rated it as having “high quality criteria” for determining the etiology of the head injuries.

The second review, Kemp et al. 2011^6^, defines three study inclusion levels for categorizing AHT cases:

1. Abuse confirmed at case conference, in family, civil, or criminal court proceedings, or admitted by a perpetrator or eyewitness;
2. Abuse confirmed by stated criteria, including multidisciplinary assessment;
3. Abuse defined by stated criteria.

Critically, in the context of this section, the review imposes no requirements regarding the role of SDH in the classification of AHT. Did case conferences consider SDH in their determinations? Did courts rely on SDH as medical evidence? Did findings of SDH precede accusations and confessions? Were multidisciplinary teams influenced by the presence of SDH in their assessments? Did the “stated criteria” include SDH as a diagnostic feature? Given the widespread assumption of an association between SDH and AHT, the review carries a high risk of including studies subject to incorporation bias and circular reasoning. Furthermore, the review makes no attempt to assess whether the “stated criteria” used in the included studies had been independently validated for diagnostic accuracy.

Sixteen of the 21 studies included in this second review were also cited in the AAP TR and have been assessed in the main body of this article as employing flawed methods for classifying AHT. Of the five remaining studies not cited in the AAP TR, two, Vinchon et al. 2004^15^, 2005^^[[23]](#endnote-24)^^ included acknowledgments by their own lead author that they were subject to “circularity bias” ^16^. That leaves three studies^17,20,^[[24]](#endnote-25)^^ one of which, Kelly & Hayes 2004^17^, was classified by the other review by Piteau et al. 2012^5^ as being of low quality in terms of classification of etiology. That study only included cases with SDH, so whatever diagnostic methods they used, all cases of AHT had SDH, as did all accidental cases. So this article cannot be used to compare rates of SDH in AHT versus accident.

Another included study, Tzioumi & Oates 1998^20^, has been shown above to have high risk of incorporation bias and circularity.

The last study, Goldstein et al. 1993^24^, classified cases as AHT “after review of all medical and social data and criminal or family court decisions.” Given that SDH is so ubiquitous in these cases, it almost certainly featured prominently in medical records and the criminal or family court decisions; this study accordingly runs a high risk of incorporation bias and circular reasoning. By contrast, cases were classified as accidental only when “events were observed and if there were no inconsistencies between the described mechanism of injury and the physical and radiologic findings” (emphasis added). The review rated this study as Level 1, the highest level, for “security of diagnosis for abuse.”

**The primary articles**

Bechtel et al 2004^7^, the first primary article cited in the AAP TR to support the claim that SDH is more common in AHT than in accidental trauma, classified a majority of cases (12 out of 15) as AHT based on having “no history of traumatic event”. Yet many infants present without a history of trauma and are not abuse victims. In this study, classification as AHT required the judgment of “an expert in the evaluation of suspected child abuse,” a process inherently dependent on that expert’s prior assumptions about which findings indicate abuse. Given that SDH was already widely associated with AHT at the time, it is highly likely that its presence influenced the diagnosis. The article thus carries a high risk of incorporation bias and circular reasoning.

The second article, Feldman et al. 2001^8^ , only includes cases with SDH and sought to determine which were accident and which were AHT. They included “corroborated, witnessed, or confessed event” as one criterion for classification as AHT but did not provide any details about these cases. The remaining criteria relied on expert opinion informed by prior assumptions about which findings indicate abuse. For example, “definite abuse” included “findings of cranial impact without history or with inadequate history of impact trauma.” The determination of inadequacy is subjective, and relies on prior assumptions. Another criterion for “definite abuse” is “multiple injuries, incompatible with normal, unintentional childhood injury”. Again this is a subjective determination and relies on preconceived ideas on what findings indicate AHT and how they differ from unintentional injury, which is the aim of the article. The methods used to classify cases as abusive are unvalidated and subjective. At best they are finding correlations between SDH and whatever findings they interpret as abusive, but no independent link to AHT is established. For classification as “definite unintentional,” a “reliable and independent” third-party witness was required—yet for classification as “definite abuse,” no such reliability or independence was required of the witness.

The third article, Duhaime et al. 1992^9^, applied predetermined criteria using an algorithm that has not been validated for its accuracy in identifying cases of AHT. The algorithm includes SDH as one of the diagnostic criteria, along with assessments of history such as “trivial trauma” (e.g., falls under 3 feet), which relies on prior assumptions about how frequently SDH results from abuse versus accidental causes. Despite this, the study is cited as establishing differences in findings between abusive and accidental head trauma. No information is provided on how many AHT cases with SDH were diagnosed, at least in part, because of the SDH itself. By including SDH in the diagnostic process, the study introduces explicit incorporation bias in its assessment of how commonly SDH occurs in AHT compared to accidental or medical causes—and carries a high risk of additional, hidden incorporation bias.

The fourth, Hymel et al. 1997^10^, categorized AHT as “determined by an experienced multidisciplinary child abuse team”. This constitutes an appeal to authority rather than a transparent diagnostic method. Nevertheless, the study received the highest rating (Level 1) for “security of diagnosis for abuse” in the review article, despite offering no insight into how abuse determinations were made, only providing information as to who made it.

The fifth, Reese & Sege 2000^11^, categorized cases as AHT those “substantiated as inflicted injury by multidisciplinary team review supplemented by comprehensive review by the medical director of the Child Protection Program.” Criteria for definite abuse included witnessed abuse, confession, and “no history accounting for the patient’s serious head injury.” This means a determination was made—during the classification stage—as to whether the “serious head injury”, including any SDH, was more consistent with abuse than accident. The study therefore carries a high risk of incorporation bias and circular reasoning, given its goal was to compare what injuries are caused by accidental versus abusive head trauma.

The sixth, Myhre et al. 2007^12^, classified cases as AHT if they were documented as abuse in the medical record and referred to child protective services, or based on the authors’ own judgment having assessed additional criteria such as “injuries where the medical history could not explain the injury”—a determination that necessarily relies on prior assumptions about the causes of accidental versus abusive trauma. Similarly, classification as accidental included the criterion “injuries evaluated as accidental in the medical record,” again incorporating prior assumptions. Given the widespread belief in an association between SDH and AHT, the study carries a high risk of incorporation bias and circular reasoning when used to compare what injuries are caused by accidental versus abusive trauma.

In the seventh and eighth studies, Ewing-Cobbs et al. 1998^13^, 2000^14^, AHT cases were determined by “assessment of the Child Protection Committee.” The authors explicitly state that, as an example of the considerations made by the committee, bilateral SDH and RH were regarded as incompatible with a history of a fall—meaning such cases would be categorized as AHT. These studies both carry a high risk of incorporation bias and circular reasoning when used to assess how commonly SDH occurs in AHT versus accidental trauma or medical causes.

**In summary, we did not identify a single study that *establishes* an association between SDH and AHT, nor any study *demonstrating* that SDH is more common in AHT than in accidental trauma, that does not carry a high risk of incorporation bias or circular reasoning—particularly the risk that experts’ prior assumptions about this association influenced the diagnosis of AHT. While it is clear that head trauma can cause SDH, the available evidence suggests that the perceived link between SDH and *abusive* head trauma is a self-fulfilling prophecy rooted in professional belief systems rather than scientific validation.**

**References**

1. Kelly P, John S, Vincent AL, Reed P. Abusive head trauma and accidental head injury: a 20-year comparative study of referrals to a hospital child protection team. Arch Dis Child. 2015;100(12):1123–1130. doi: 10.1136/archdischild-2014-306960 [↑](#endnote-ref-2)
2. Bradford R, Choudhary AK, Dias MS. Serial neuroimaging in infants with abusive head trauma: timing abusive injuries. J Neurosurg Pediatr. 2013;12(2):110–9. doi: 10.3171/2013.4.PEDS12596 [↑](#endnote-ref-3)
3. Dias MS, Backstrom J, Falk M, Li V. Serial radiography in the infantshaken impact syndrome. Pediatr Neurosurg. 1998;29(2):77–85. doi: 10.1159/000028694 [↑](#endnote-ref-4)
4. Matschke J, Voss J, Obi N, et al. Nonaccidental head injury is the most common cause of subdural bleeding in infants <1 year of age. Pediatrics. 2009;124(6):1587–1594. doi: 10.1542/peds. 2008-3734 [↑](#endnote-ref-5)
5. Piteau SJ, Ward MG, Barrowman NJ, Plint AC. Clinical and radiographic characteristics associated with abusive and nonabusive head trauma: a systematic review. Pediatrics. 2012;130(2):315–323. doi: 10.1542/peds.2011-1545 [↑](#endnote-ref-6)
6. Kemp AM, Jaspan T, Grif ths J, et al. Neuroimaging: what neuroradiological features distinguish abusive from non-abusive head trauma? A systematic review. Arch Dis Child. 2011;96(12): 1103–1112. doi: 10.1136/archdischild-2011-300630 [↑](#endnote-ref-7)
7. Bechtel K, Stoessel K, Leventhal JM, et al. Characteristics that distinguish accidental from abusive injury in hospitalized young children with head trauma. Pediatrics. 2004;114(1):165–168. doi: 10.1542/peds.114.1.165 [↑](#endnote-ref-8)
8. Feldman KW, Bethel R, Shugerman RP, Grossman DC, Grady MS, Ellenbogen RG. The cause of infant and toddler subdural hemorrhage: a prospective study. Pediatrics. 2001;108(3):636–646. [↑](#endnote-ref-9)
9. Duhaime AC, Alario AJ, Lewander WJ, et al. Head injury in very young children: mechanisms, injury types, and ophthalmologic findings in 100 hospitalized patients younger than 2 years of age. Pediatrics. 1992;90(2 Pt 1):179–185 [↑](#endnote-ref-10)
10. Hymel KP, Rumack CM, Hay TC, et al. Comparison of intracranial computed tomographic (CT) fi ndings in pediatric abusive and accidental head trauma. Pediatr Radiol 1997;27:743–7. [↑](#endnote-ref-11)
11. Reece RM, Sege R. Childhood head injuries: accidental or inflicted? Arch Pediatr Adolesc Med. 2000;154(1):11–15 [↑](#endnote-ref-12)
12. Myhre MC, Grogaard JB, Dyb GA, Sandvik L, Nordhov M. Traumatic head injury in infants and toddlers. Acta Paediatr. 2007;96(8): 1159–1163. doi: 10.1111/j.1651-2227.2007.00356.x [↑](#endnote-ref-13)
13. Ewing-Cobbs L, Kramer L, Prasad M, et al. Neuroimaging, physical, and developmental findings after inflicted and noninflicted traumatic brain injury in young children. Pediatrics. 1998;102(2 Pt 1): 300–307. doi: 10.1542/peds.102.2.300 [↑](#endnote-ref-14)
14. Ewing-Cobbs L, Prasad M, Kramer L, et al. Acute neuroradiologic findings in young children with inflicted or noninflicted traumatic brain injury. Childs Nerv Syst. 2000;16(1):25–33. doi: 10.1007/s003810050006 [↑](#endnote-ref-15)
15. Vinchon M, Defoort-Dhellemmes S, Desurmont M, Dhellemmes P. Accidental and nonaccidental head injuries in infants: a prospective study. J Neurosurg. 2005;102(suppl 4): 380–384 [↑](#endnote-ref-16)
16. Vinchon M, de Foort-Dhellemmes S, Desurmont M, Delestret I.Confessed abuse versus witnessed accidents in infants: comparison of clinical, radiological, and ophthalmological data in corroborated cases. Childs Nerv Syst. 2010;26(5):637–645.doi: 10.1007/s00381-009-1048-7 [↑](#endnote-ref-17)
17. Kelly P, Hayes I. Infantile subdural haematoma in Auckland, New Zealand: 1988-1998.N Z Med J. 2004;117(1201):U1047 [↑](#endnote-ref-18)
18. Ruppel RA, Kochanek PM, Adelson PD, et al. Excitatory amino acid concentrations in ventricular cerebrospinal uid after severe traumatic brain injury in infants and children: the role of child abuse. J Pediatr.2001;138(1):18–25 [↑](#endnote-ref-19)
19. Billmire ME, Myers PA. Serious head injury in infants: accident or abuse? Pediatrics. 1985;75(2):340–342 [↑](#endnote-ref-20)
20. Tzioumi D, Oates RK. Subdural hematomas in children under 2 years. Accidental or inflicted? A 10-year experience. Child Abuse Negl. 1998;22(11):1105–1112 [↑](#endnote-ref-21)
21. Hobbs CJ. Skull fracture and the diagnosis of abuse. Arch Dis Child. 1984;59(3):246–252 [↑](#endnote-ref-22)
22. Vavilala MS, Muangman S, Waitayawinyu P,et al. Neurointensive care; impaired cerebral autoregulation in infants and young children early after inflicted traumatic brain injury: a preliminary report. J Neurotrauma. 2007;24(1):87–96 [↑](#endnote-ref-23)
23. Vinchon M, Noulé N, Jissendi-Tchofo P, Soto-Ares G, Fourier C, Dhellemmes P (2004) Imaging of head injuries in infants: temporal correlates and implications for the diagnosis of child abuse. J Neurosurg 101(1 Suppl):44–52 [↑](#endnote-ref-24)
24. Goldstein B, Kelly MM, Bruton D, et al. Inflicted versus accidental head injury in critically injured children. Crit Care Med 1993;21:1328–32 [↑](#endnote-ref-25)
