# Supplementary material for: A critical review of the American Academy of Pediatrics technical report on abusive head trauma
Source: Forensic Sci Int Synerg. 2025 Dec 3;11:100650. doi: 10.1016/j.fsisyn.2025.100650 (PMC12721060; doi:10.1016/j.fsisyn.2025.100650)
Supplement: Multimedia component 2 [file mmc2.docx]

**A Critical Review of the American Academy of Pediatrics Technical Report on Abusive Head Trauma**

**Appendix 2**

**Retinal Hemorrhage (RH)**

In this appendix, we analyze citations in the section *Physical Findings*, subsection *f) Ocular Findings, 2. Findings* (p. 11). This subsection purports to provide evidence for the association of specific ocular findings with AHT.

- The AAP TR states that “retinal hemorrhages are the most frequent and most sensitive acute sign of AHT”.

It cites a review article, Bhardwaj et al. 2010^^[[1]](#endnote-2)^^, that compiled 973 AHT cases from 20 observational studies and found RH in about 80%, with a mean sensitivity of 75% for AHT. The review itself acknowledges that “there was the potential for circular logic in all but 4 studies, because IOH [intraocular hemorrhages] are often used clinically as a diagnostic sign of abuse.” In other words, the authors recognized that 16 of the included studies were at risk of circular reasoning. That leaves only four^^[[2]](#endnote-3)^,^[[3]](#endnote-4)^,^[[4]](#endnote-5)^,^[[5]](#endnote-6)^^ studies.

The first, Gilles et al. 2003^2^, classified cases as AHT based on “witnessed events, confession, felony conviction, or minimal or absent trauma history in the presence of clear and convincing evidence of major head trauma.” However, it provided no information on how many cases fell under each classification criterion, no details about the nature of the witnessed events or confessions, and no clarification as to whether RHs were part of the findings that led to initial suspicions, accusations, or interrogations. Similarly, it did not specify whether RHs contributed to the medical evidence used in the convictions, nor did it define what was considered “clear and convincing evidence of major head trauma” or whether that included RHs or correlated findings such as subdural hematomas (SDH). This study clearly carries a high risk of incorporation bias and circular reasoning.

The second, Bechtel et al. 2004³, categorizes a statistically dominant number (12 of 15) of cases as AHT due to having “no history of traumatic event”. However, many infants present to hospital without a history of trauma and are not classified as AHT cases. The classification as AHT relied on the judgment of “an expert in the evaluation of suspected child abuse,” who attributed the findings to abuse—an attribution that inherently assumes a pre-existing association between particular findings and abuse. Although Bechtel did not include RH directly in this medical assessment, they did include SDH, which is highly correlated. The article has high risk of indirect circular reasoning with respect to RH: the study confirms an association between the findings used to classify the cases as AHT, but does not confirm that the cases were actually true AHT cases.

The third, Kivlin et al. 2000^4^, included cases with SDH that were diagnosed as shaken baby syndrome “by Child Advocacy physicians based on the presence of subdural hematomas, characteristic bone injuries, and the absence of a history of compatible accidental trauma.” Their finding of RH in these cases confirms the long-known association between SDH and RH^^[[6]](#endnote-7)^^, but does not by itself establish any link to AHT—unless the diagnostic criteria used to define AHT are themselves valid and accurate. However, we are not aware of any studies that have established the reliability of diagnosing AHT based solely on SDH, bone injuries, and a lack of reported trauma. Even if such criteria were valid, it remains unclear whether they were rigorously applied in these cases, as the article does not provide details about the specific findings that led to any of the AHT diagnoses.

The fourth, Pierre-Kahn et al. 2003^5^, included cases with SDH, where a “clotting screen and blood ion-concentration evaluation were done to rule out other possible causes of spontaneous SDH.” The categorization of these cases as AHT thus relied on the assumption that AHT can be accurately diagnosed based on the presence of SDH, after ruling out other causes using the blood ion-concentration evaluation. Of the 231 cases included, 186 showed no clinical or radiologic evidence of direct impact to the skull—meaning there was no corroborating evidence of trauma, let alone abusive trauma. Again, the study ultimately shows the long known link between SDH and RH^6^, but does not establish a link to AHT.

- The AAP TR then states that “The extent, distribution, and pattern of retinal hemorrhages and other associated ophthalmic findings determine their specificity, which can be as high as 94% for abuse.”

For this, the AAP TR cites the same review article, Bhardwaj et al. 2010^1^ discussed above, which included 16 of 20 studies that the study itself identified as being at risk of circular reasoning, and the remaining four with other significant methodological flaws as mentioned.

- The AAP TR then states that “ocular findings of AHT may include retinal folds, retinoschisis, papilledema, and optic nerve sheath and orbital fat hemorrhage on autopsy,” and that some “vitreoretinal findings of trauma… may require specialized retinal imaging (ie, optical coherence tomography) to identify and may be seen on autopsy in fatal injuries.”

Three citations^^[[7]](#endnote-8)^,^[[8]](#endnote-9)^,^[[9]](#endnote-10)^^ are provided. The first, Muni et al. 2010^7^, offers no explanation as to why the three cases examined were suspected of being SBS—they were selected based only on a suspicion. The purpose of the study was to examine cases with extensive RH using hand-held spectral domain optical coherence tomography, not to establish how or why these cases were diagnosed as SBS or to establish an association between any type of RH and AHT.

The second, Sturm et al. 2008^8^ also involved retcam imaging of three patients with “presumed shaken baby syndrome”, without details of what led to the suspicion.

The third, Koozekanani et al 2010^9^ , was similar —a single case study of a *presumed* SBS victim, presenting results from spectral domain optical coherence tomography.

- The AAP TR then states that “Poor vision on presentation, decreased pupillary response, papilledema, and severe retinal hemorrhages and retinal folds are ophthalmologic findings of AHT associated with increased mortality.”

Four citations^4,^[[10]](#endnote-11)^,^[[11]](#endnote-12)^,^[[12]](#endnote-13)^^ are provided. The first, Kivlin et al. 2000^4^, does not establish an association between ocular findings and AHT as already discussed above.

The second, Mills 1998^10^, includes cases with subdural hemorrhages (SDH) and RH that were assessed as consistent with Shaken Baby Syndrome. These were cases where “medical conditions causing bleeding were excluded by medical evaluation” and “the history in each case was inconsistent with an etiology of accidental trauma.” This medical information was considered “along with the conclusions of social workers and police.” However, the article does not clarify whether those conclusions were based on the medical opinions of the physicians involved. Notably, “no patient had external evidence of significant direct head or eye trauma,” meaning there was no corroborating medical evidence of trauma. The only information provided is that the patients died, had SDH and RH, and that doctors were unable to identify an underlying medical condition—leading them to presume shaking as the cause. RH was explicitly part of the case selection criteria and, at the time, was already considered closely associated with abuse. The study therefore suffers from incorporation bias and does not provide evidence that establishes an independent association between RH and AHT.

In the third, McCabe & Donahue 2000^11^, a “diagnosis of SBS was considered” when “bilateral retinal hemorrhages were observed in a situation where the injury was not consistent with the history, when other obvious signs of abuse were present, or if there had been a history of a previous suspicious episode in a lethargic infant.” Once again, bilateral retinal hemorrhages were a key component of the diagnostic criteria, meaning this study cannot be used to establish an independent association between bilateral RH and AHT.

The fourth, Wilkinson et al. 1989^12^, defined Shaken Baby Syndrome cases as those with “findings of intraocular hemorrhage and intracranial injury in the absence of external signs of head trauma.” Because retinal hemorrhage was part of the inclusion criteria, this study has explicit incorporation bias and cannot be used to demonstrate an association between bilateral RH and AHT.

We do note that, taken together, these four studies do suggest a general trend: more severe intracranial pathology appears to be associated with a greater number and severity of ophthalmologic findings.

- The AAP TR then discusses retinal folds and their characteristics, stating that their “incidence increases to 23% to 42% in severe or fatal cases of AHT”.

Four citations^1,^[[13]](#endnote-14)^,^[[14]](#endnote-15)^,^[[15]](#endnote-16)^^ are provided. The first, Bhardwaj et al. 2010^1^, is analysed above. The second, Breazzano et al. 2014^13^, involves AHT cases that—except for one—were “legally verified by confession or conviction.” The study provides no details on the medical findings that contributed to these convictions, nor does it clarify the circumstances or content of the confessions, including whether they occurred after medical evaluations and accusations were made. The number of cases involving confessions is not disclosed.

The third, Massicotte et al. 1991^14^, consists of a three case series involving infants who purportedly “died after episodes of severe shaking.” In the first case, a “complete forensic investigation determined that the child had been violently shaken,” though no specific medical or forensic criteria are described. In the second case, the basis for the AHT diagnosis is also unclear. A confession to shaking was reportedly made by a 12-year-old babysitter, but this would be inconsistent with the presence of multiple occipital skull fractures—injuries more aligned with her statement that the infant fell in the bath and struck its head. The third case involved “contusions on the cheeks and around the ears,” which were “considered evidence of violent shaking,” presumably in combination with the presence of SDH and RH. This diagnostic approach has not been independently validated and carries a high risk of circular reasoning.

The fourth, Gaynon et al. 1988^15^, consists of two case series involving “presumed child abuse victims.” In the first case, there were no “external signs of contusion or other trauma,” yet the child “was placed in the custody of the court because of the suspicion of child abuse.” In the second case, the caregiver reported a 5-foot fall while holding the child, but no specific reason was given for suspecting abuse. In both cases, subdural hemorrhages (SDH) and retinal hemorrhages (RH) were explicitly noted, and seemingly treated as providing sufficient evidence for a diagnosis of AHT.

Once again, none of these four studies *establish* any association between RH and AHT; they assume it. However, collectively, the four studies do offer some evidence suggesting that retinal folds may be associated with the *severity* of intracranial pathology.

**In summary, while the studies in this subsection highlight potential patterns and correlations in ocular and intracranial findings—some of which are associated with trauma—none provide independent, methodologically sound evidence of a specific diagnostic association between any ocular findings and *abusive* head trauma.**

**References**

1. Bhardwaj G, Chowdhury V, Jacobs MB, Moran KT, Martin FJ, Coroneo MT. A systematic review of the diagnostic accuracy of ocular signs in pediatric abusive head trauma. Ophthalmology. 2010;117(5):983–992.e17. [↑](#endnote-ref-2)
2. Gilles E, McGregor ML, Levy-Clarke G. Retinal hemorrhage asymmetry in inflicted head injury: a clue to pathogenesis? J Pediatr 2003;143:494 –9. [↑](#endnote-ref-3)
3. Bechtel K, Stoessel K, Leventhal JM, et al. Characteristics that distinguish accidental from abusive injury in hospitalized young children with head trauma. Pediatrics 2004;114: 165– 8. [↑](#endnote-ref-4)
4. Kivlin JD, Simons KB, Lazoritz S, Ruttum MS. Shaken baby syndrome. Ophthalmology 2000;107:1246 –54. [↑](#endnote-ref-5)
5. Pierre-Kahn V, Roche O, Dureau P, et al. Ophthalmologic findings in suspected child abuse victims with subdural hematomas. Ophthalmology 2003;110:1718 –23. [↑](#endnote-ref-6)
6. Till K. Subdural Haematoma and Effusion in Infancy *Br Med J*1968; 3 :400 [↑](#endnote-ref-7)
7. Muni RH, Kohly RP, Sohn EH, Lee TC. Hand-held spectral domain optical coherence tomography finding in shaken-baby syndrome. Retina. 2010;30(4 Suppl):S45–S50. doi: 10.1097/IAE. 0b013e3181dc048c [↑](#endnote-ref-8)
8. Sturm V, Landau K, Menke MN. Optical coherence tomography findings in shaken baby syndrome. Am J Ophthalmol. 2008; 146(3):363–368. doi: 10.1016/j.ajo.2008.04.023 [↑](#endnote-ref-9)
9. Koozekanani DD, Weinberg DV, Dubis AM, Beringer J, Carroll J. Hemorrhagic retinoschisis in shaken baby syndrome imaged with spectral domain optical coherence tomography. Ophthalmic Surg Lasers Imaging. 2010 Mar 9:1–3. [↑](#endnote-ref-10)
10. Mills M. Funduscopic lesions associated with mortality in shaken baby syndrome. J AAPOS. 1998;2(2):67–71. [↑](#endnote-ref-11)
11. McCabe CF, Donahue SP. Prognostic indicators for vision and mortality in shaken baby syndrome. Arch Ophthalmol. 2000;118(3): 373–377. [↑](#endnote-ref-12)
12. Wilkinson WS, Han DP, Rappley MD, Owings CL. Retinal hemorrhage predicts neurologic injury in the shaken baby syndrome. Arch Ophthalmol. 1989;107(10):1472–1474. doi: 10.1001/archopht. 1989.01070020546037 [↑](#endnote-ref-13)
13. Breazzano MP, Unkrich KH, Barker-Grif th AE. Clinicopathological findings in abusive head trauma: analysis of 110 infant autopsy eyes. Am J Ophthalmol. 2014;158(6):1146–1154.e2. doi: 10.1016/j.ajo.2014.08.011 [↑](#endnote-ref-14)
14. Massicotte SJ, Folberg R, Torczynski E, Gilliland MG, Luckenbach MW. Vitreoretinal traction and perimacular retinal folds in the eyes of deliberately traumatized children. Ophthalmology. 1991; 98(7):1124–1127. doi: 10.1016/s0161-6420(91)32167-5 [↑](#endnote-ref-15)
15. Gaynon MW, Koh K, Marmor MF, Frankel LR. Retinal folds in the shaken baby syndrome. Am J Ophthalmol. 15 1988;106(4): 423–425. doi: 10.1016/0002-9394(88)90877-x [↑](#endnote-ref-16)
