# Supplementary material for: A critical review of the American Academy of Pediatrics technical report on abusive head trauma
Source: Forensic Sci Int Synerg. 2025 Dec 3;11:100650. doi: 10.1016/j.fsisyn.2025.100650 (PMC12721060; doi:10.1016/j.fsisyn.2025.100650)
Supplement: Multimedia component 3 [file mmc3.docx]

**A Critical Review of the American Academy of Pediatrics Technical Report on Abusive Head Trauma**

**Appendix 3**

**Diagnosis**

In this appendix we analyse citations in the section *Diagnosis* including the subsection *Prediction Rules and Pooled Analyses* (p 33.)

- The Diagnosis section (p33) states that “the diagnosis of AHT is made in the context of a complete history, physical examination, and medical evaluation using the same process as in any other medical diagnosis: the diﬀerential diagnosis.”

The sole citation is to a 2020 AAP guideline^^[[1]](#endnote-1)^^ with the same lead author as the current AAP TR. Notably, in the context of AHT, the differential diagnosis process functions as a “diagnosis by exclusion”: when findings, or constellations of findings, believed to be associated with AHT are present, AHT is presumed unless another cause is explicitly identified and accepted.

- The AAP then states that “In addition, many providers have the advantage of a multidisciplinary hospital child protection team.”

One citation is provided, Tien et al. 2002^^[[2]](#endnote-2)^^, but it does not demonstrate any diagnostic advantage of such teams; it merely notes that they are commonly used.

- The AAP TR continues, stating that “such a team can help to minimize the risk of individual error that may occur from judgment heuristics or cognitive bias.”

While a citation is given to a narrative review explaining heuristics and biases by Kahneman 2003^^[[3]](#endnote-3)^^, no evidence is presented that multidisciplinary teams reduce these risks in the diagnostic process for AHT.

- The AAP TR then states that “Throughout the diagnostic differentiation process, the pediatric provider will have to manage various uncertainties until the individual threshold of diagnostic suﬃciency is reached.”

Narang et al. 2019^^[[4]](#endnote-4)^^ is cited in support of this statement, but that article does not explain how such uncertainties should be managed in practice. In particular, if multiple differential diagnoses are each considered “unlikely,” it is unclear how these likelihoods should be weighed or summed to reach diagnostic sufficiency. Furthermore, the cited article notes that “the differential diagnosis may include … unrecognized medical conditions.” It is unclear how a differential diagnosis can effectively account for—or exclude—conditions that are unrecognized. Finally, no justification is made as to why AHT should be the “default” diagnosis, the one that is presumed in absence of identifying another cause.

***Prediction Rules and Pooled Analyses***

- The AAP TR says that “Assisting providers in reaching a diagnosis are recently published prediction rules and pooled analyses.. evidence-based tools that combine clinical features to assist clinicians in probabilistic determinations of clinical diagnosis…”

Citation is made to McGinn et al. 2000^^[[5]](#endnote-5)^^, a guide for developing and using clinical decision rules (CDRs). Tellingly, the example provided in the CDR guide is the Ottawa Ankle Rules—a CDR for determining when an ankle x-ray is necessary—which was validated against a clear gold standard: the radiographic findings. By contrast, the CDRs used for diagnosing AHT lack such a gold reference standard and have not been validated. This limitation is evident in the tools cited by the AAP TR: the PredAHT tool, the PEDIBIRN clinical decision rule, and Maguire’s pooled analysis.

**Maguire’s pooled analysis**^^[[6]](#endnote-6)^^ included cases from six studies^^[[7]](#endnote-7)^,^[[8]](#endnote-8)^,^[[9]](#endnote-9)^,^[[10]](#endnote-10)^,^[[11]](#endnote-11)^,^[[12]](#endnote-12)^^, each required to meet the two highest levels of AHT classification for a study´s inclusion:

1. Abuse confirmed through case conference, civil/family/criminal court proceedings, perpetrator admission, or independent eyewitness testimony; and
2. Abuse confirmed by stated criteria, such as multidisciplinary assessment.

These criteria carry a high risk of including studies that are subject to incorporation bias and circular reasoning. Except for independently witnessed cases—which are rare, and absent in most of the cited studies—the classifications rely on expert or multidisciplinary assessment of medical findings, resting on prior assumptions about which findings are indicative of AHT (this includes confessions made after medical evaluations and accusations). As a result, these assessments presuppose the very association they aim to demonstrate—meaning they cannot reliably be used to establish which medical findings are actually diagnostic of AHT. The following section examines the categorisation methods used in each of the six included studies.

The first, Hobbs et al. 2005^7^, relied on “clinicians’ stated aetiology… based on the clinical history, examination, and investigations, which in some cases included a child protection investigation.” So the classification of AHT was determined using clinical history and related findings—criteria that overlap with the very features the pooled analysis later seeks to associate with AHT. The use of this study in the pooled analysis introduces a high risk of incorporation bias and circular reasoning.

The second, Bechtel et al. 2004,^8^ classified a statistically dominant number of cases (12 out of 15) as AHT on the basis of “no history of traumatic event.” In such cases, classification as AHT required the judgment of “an expert in the evaluation of suspected child abuse,” a determination based on whether observed findings were indicative of AHT. This reliance on prior assumptions introduces a high risk of incorporation bias and circular reasoning.

In the third, Ettaro et al. 2004^9^ , one of the authors “categorized patients into groups based on a combination of historical, radiographic, and physical examination information”, applying the criteria set out in Duhaime 1992^^[[13]](#endnote-13)^^, which themselves have not been validated, and require subjective interpretations of what can cause the findings in each case. Some findings such as SDH, are explicitly included in the algorithm and are also included as clinical features of AHT in the Maguire pooled analysis, meaning that there is explicit incorporation bias when using data from this study, as well as likely hidden incorporation bias.

The fourth, Hettler & Greenes 2003^10^, required intracranial hemorrhage (explicit incorporation bias when used in Maguire´s pooled analysis), as well as one or more of the following: witnessed or confessed abuse, retinal hemorrhages consistent with abuse, high-specificity skin findings (pattern marks), or moderate- or high-specificity fractures. This introduces further explicit incorporation bias. Highly likely there is further hidden incorporation bias involved in the confessed cases as they were made after medical evaluation and after accusations. Only one case (of 49) was classified as definite abuse due to being witnessed. No findings in that single case were provided, nor were details on whether the witnmesses was independent.

The fifth, Kemp et al. 2003^11^, classified as AHT: (1) head injury where there was a confession by the perpetrator (n = 19); (2) cases where AHT was established as a result of criminal conviction in the criminal court where there were unexplained extracranial injuries (n = 10); (3) cases where there were unexplained injuries elsewhere in the body, other than head injury, but no conviction (all diagnosed at case conference except two who died) (n = 15); and (5) cases where there was major discrepancy between the explanation given by the carer and significant injury, such as a skull fracture, or if the history was developmentally incompatible (AHT diagnosed at case conference in all except one who died) (n = 21).

It is commendable that this study provided numbers as to how many cases met each criterion. No details of confessions were provided, including whether particular medical findings led to accusations (were those findings also “clinical features” of AHT in Maguire´s pooled analysis?). No details of what were the unexplained extracranial injuries, and whether they contributed to the criminal conviction (almost certainly the unexplained extracranial injuries that were used in diagnosis are also “clinical features” of AHT in Maguire´s pooled analysis). Were any of the “unexplained injuries elsewhere in the body” also “clinical features” of AHT in Maguire´s pooled analysis? What about the findings that led to the case conference diagnosis in criteria 3 and 5? Including this study in Maguire´s pooled analysis leads to explicit and likely further hidden incorporation bias, and high risk of circular reasoning.

The sixth, Vinchon et al. 2005^12^, classified AHT by multidisciplinary team diagnosis, using an algorithm described as “similar to that used by Duhaime^^[[14]](#endnote-14)^^ and coworkers.” This was one of the studies that Vinchon himself acknowledged “it was difficult to validate the diagnostic value of this grading because of the circularity bias”^^[[15]](#endnote-15)^^.

What Maguire’s pooled analysis does do is identify which clinical findings are commonly used by experts and multidisciplinary teams to diagnose AHT. However, due to incorporation bias and inherent circularity, the analysis does not prove that these findings are diagnostic of AHT. It does not validate the diagnostic process, nor does it provide a meaningful measure of diagnostic accuracy.

**The PredAHT tool** is built on the flawed foundation of Maguire´s pooled analysis. The citation provided for PredAHT, Pfeifer et al. 2020^^[[16]](#endnote-16)^^, claims to “validate” the tool. They do so using a sample of AHT cases determined by multidisciplinary child protection teams—teams that relied on the same types of findings included in Maguire’s analysis. These teams categorized cases as AHT or non-AHT based on retrospective review of their own investigative records, citing “standard” child protection assessment processes in Australia and New Zealand, which the study does not define.

In other words, the tool was validated using diagnoses that were themselves informed, directly or indirectly, by the very findings the tool claims to test. This is not independent validation; it is circular reasoning. The PredAHT was developed from a process compromised by incorporation bias, and then “validated” using the same “logic”. This fundamentally undermines its credibility as a diagnostic tool.

**The PediBIRN clinical decision rule** was presented in Hymel et al. 2019^^[[17]](#endnote-17)^^. AHT was determined in two ways; by predetermined criteria and also by the final determination of the physician. Clearly, the latter is authoritative medicine, and the findings of cases categorised as AHT simply reflect the prior assumptions of those physicians as to what findings are associated with AHT, introducing high risk of incorporation bias and circularity.

The predetermined criteria are: (1) an admission of abuse by the caregiver, (2) abuse witnessed by an independent observer, (3) caregiver denial of any head trauma, (4) caregiver account of the head injury event that was clearly historically inconsistent with repetition over time, (5) developmentally implausible explanations, and (6) the presence of certain injuries such as patterned bruising, burns, or intra-abdominal injury.

In practice, very few cases in the PediBIRN dataset were based on witnessed events. The confessions occurred after the child had already undergone medical evaluation and clinicians had identified findings commonly associated with abuse.

Reliance on caregiver denial as a marker of abuse is also flawed. As Brook et al. 2023^^[[18]](#endnote-18)^^ demonstrated using the PediBIRN dataset itself, cases in which caregivers denied any head trauma showed significantly fewer signs of trauma—such as bruising or skull fractures—than cases where trauma was reported. In other words, the clinical evidence supports these denials rather than contradicts them. The only way such cases can be classified as AHT is if physicians interpret certain *intracranial* findings (e.g. subdural hemorrhage, cerebral edema) as necessarily traumatic and of the kind presumed to result from abuse. Thus, the cases classified in such a manner also have high risk of incorporation bias and circularity.

The next predetermined criteria used to classify AHT in the PediBIRN study is that the caregiver gave an account of the injury that was “historically inconsistent with repetition over time.” This refers to the caregiver offering differing explanations across multiple interviews. However, these changes occur only after the case has already been flagged as suspicious for abuse based on medical findings. At that point, clinicians challenge the initial explanation, asserting that it cannot account for the injuries observed. In response, the caregiver may offer other possibilities in an effort to explain the unexplained. These shifts are then interpreted as evidence of deception and guilt. Crucially, the process begins with a suspicion of abuse based on the medical findings. As such, this criteria is similarly at risk of incorporation bias and circular reasoning.

The next criterion, developmentally implausible explanations, also depends on prior assumptions about AHT. Caregiver explanations are evaluated only after suspicion is triggered by medical findings indicative of AHT. If the reported mechanism is deemed incompatible with those findings, it is dismissed, and this is used as a diagnostic criterion for abuse. This judgment relies on the same prior assumptions about medical findings that initially led to suspicion, thereby introducing incorporation bias and circular reasoning.

The criteria used to classify cases as AHT for the “validation” of the PediBIRN clinical decision rule have never themselves been independently validated and all rely heavily on prior assumptions about which findings are specific to AHT. The sensitivity, specificity, and predictive values reported do not reflect the true accuracy of the rule, as this would require a reliably and objectively determined set of AHT cases, one not defined by the very features the rule seeks to measure.

**In summary, the AAP TR discussion of AHT diagnosis reveals that the process is fundamentally rooted in expert and multidisciplinary team (MDT) determinations —forms of authoritative medicine. While the AAP TR presents MDTs as safeguards against bias, it provides no evidence that they improve diagnostic accuracy. The AAP TR cites clinical decision rules (CDRs) and pooled analyses, but these are both developed from, and validated against the same expert/MDT opinions, introducing incorporation bias and circular reasoning. Because no independent standard is used for AHT determination, no meaningful measure of diagnostic accuracy is possible in either the pooled analysis or the CDRs. As a result, AHT diagnosis remains grounded in expert opinion, not evidence-based methodology.**

1. Narang SK, Fingarson A, Lukefahr J; American Academy of Pediatrics, Council on Child Abuse and Neglect. Abusive head trauma in infants and children. Pediatrics. 2020;145(4): e20200203. doi: 10.1542/peds.2020-0203 [↑](#endnote-ref-1)
2. Tien I, Bauchner H, Reece RM. What is the system of care for abused and neglected children in children’s institutions? Pediatrics. 2002;110(6):1226–1231. doi: 10.1542/peds.110.6.1226 [↑](#endnote-ref-2)
3. Kahneman D. Maps of bounded rationality: a perspective on intuitive judgment and choice. The American Economic Review. 2003; 93(5):1449–1475 [↑](#endnote-ref-3)
4. Narang S, Campbell KA, Simonton K. Reporting abuse, managing uncertainty, and other legal issues. In: Laskey A, Sirotnak A, eds. Child Abuse: Medical Diagnosis and Management. American Academy of Pediatrics; 2019:875–920 465. McGinn TG, Guyatt GH, Wyer PC, Naylor CD [↑](#endnote-ref-4)
5. McGinn TG, Guyatt GH, Wyer PC, Naylor CD, Stiell IG, Richardson WS. Users’ guides to the medical literature: XXII: how to use articles about clinical decision rules. Evidence-Based Medicine Working Group. JAMA. 2000;284(1):79–84. [↑](#endnote-ref-5)
6. Maguire SA, Kemp AM, Lumb RC, Farewell DM. Estimating the probability of abusive head trauma: a pooled analysis. Pediatrics. 2011;128(3):e550–e64. doi: 10.1542/peds.2010-2949 [↑](#endnote-ref-6)
7. Hobbs C, Childs AM, Wynne J, Livingston J, Seal A. Subdural haematoma and effusion in infancy: an epidemiological study. Arch Dis Child. 2005;90(9):952–955 [↑](#endnote-ref-7)
8. Bechtel K, Stoessel K, Leventhal JM, et al. Characteristics that distinguish accidental from abusive injury in hospitalized young children with head trauma. Pediatrics. 2004;114(1):165–168 [↑](#endnote-ref-8)
9. Ettaro L, Berger RP, Songer T. Abusive head trauma in young children: characteristics and medical charges in a hospitalized population. Child Abuse Negl. 2004;28(10): 1099 –111 [↑](#endnote-ref-9)
10. Hettler J, Greenes DS. Can the initial history predict whether a child with a head injury has been abused? Pediatrics. 2003;111(3):602– 607 [↑](#endnote-ref-10)
11. Kemp AM, Stoodley N, Cobley C, Coles L, Kemp KW. Apnoea and brain swelling in non-accidental head injury. Arch Dis Child. 2003;88(6):472– 476 [↑](#endnote-ref-11)
12. Vinchon M, Defoort-Dhellemmes S, Desurmont M, Dhellemmes P. Accidental and non-accidental head injuries in infants: a prospective study. J Neurosurg. 2005;102(suppl 4):380 –384 [↑](#endnote-ref-12)
13. Duhaime AC, Alario AJ, Lewander WJ, et al. Head injury in very young children: mechanisms, injury types, and ophthalmologic findings in 100 hospitalized patients younger than 2 years of age. Pediatrics. 1992;90(2 Pt 1):179–185 [↑](#endnote-ref-13)
14. Duhaime AC, Alario AJ, Lewander WJ, et al. Head injury in very young children: mechanisms, injury types, and ophthalmologic findings in 100 hospitalized patients younger than 2 years of age. Pediatrics. 1992;90(2 Pt 1):179–185 [↑](#endnote-ref-14)
15. Vinchon M, de Foort-Dhellemmes S, Desurmont M, Delestret I.Confessed abuse versus witnessed accidents in infants: comparison of clinical, radiological, and ophthalmological data in corroborated cases. Childs Nerv Syst. 2010;26(5):637–645.doi: 10.1007/s00381-009-1048-7 [↑](#endnote-ref-15)
16. Pfeiffer H, Cowley LE, Kemp AM, et al. Validation of the PredAHT-2 prediction tool for abusive head trauma. Emerg Med J. 2020;37(3): 119–126. doi: 10.1136/emermed-2019-208893 [↑](#endnote-ref-16)
17. Hymel KP, Wang M, Chinchilli VM, et al. Estimating the probability of abusive head trauma after abuse evaluation. Child Abuse Negl. 2019;88:266–274. doi: 10.1016/j.chiabu.2018.11.015 [↑](#endnote-ref-17)
18. Brook, C.B. (2023), Data-driven evidence shows truthful caregiver histories and significant overdiagnosis of abusive head trauma. Ann Child Neurol Soc, 1: 299-304. [↑](#endnote-ref-18)
