# Supplementary material for: A critical review of the American Academy of Pediatrics technical report on abusive head trauma
Source: Forensic Sci Int Synerg. 2025 Dec 3;11:100650. doi: 10.1016/j.fsisyn.2025.100650 (PMC12721060; doi:10.1016/j.fsisyn.2025.100650)
Supplement: Multimedia component 4 [file mmc4.docx]

**A Critical Review of the American Academy of Pediatrics Technical Report on Abusive Head Trauma**

**Appendix 4**

**Systematic Reviews**

Several systematic reviews and/or pooled analysis are cited in the AAP TR, eight of them^^[[1]](#endnote-1)^,^[[2]](#endnote-2)^,^[[3]](#endnote-3)^,^[[4]](#endnote-4)^,^[[5]](#endnote-5)^,^[[6]](#endnote-6)^,^[[7]](#endnote-7)^,^[[8]](#endnote-8)^^ aim to identify findings specific to AHT, or diagnostic of AHT. We have already analysed five of these reviews, two^1,2^ in Appendix 1, one^3^ in Appendix 2 and two^4,5^ in Appendix 3. We looked at each of the studies that they relied upon, and found that those studies suffered the same methodological flaws as the primary articles analysed in the main text of our article. In other words, their quality control procedures did not involve assessing whether their included studies had a risk of incorporation bias and/or circular reasoning. The vast majority of the primary studies cited in these reviews were also cited in the AAP TR.

Here, we examine the remaining three systematic reviews whose subject matter is relevant to our analysis of the AAP TR. We note that the AAP TR also cites several narrative reviews; however, we do not analyse these, as such articles hold no substantive scientific value.

**Maquire 2009**^6^ reviews the literature to (purportendly) determine which clinical features distinguish inflicted from non-inflicted brain injury. 14 studies^^[[9]](#endnote-9)^,^[[10]](#endnote-10)^,^[[11]](#endnote-11)^,^[[12]](#endnote-12)^,^[[13]](#endnote-13)^,^[[14]](#endnote-14)^,^[[15]](#endnote-15)^,^[[16]](#endnote-16)^,^[[17]](#endnote-17)^,^[[18]](#endnote-18)^,^[[19]](#endnote-19)^,^[[20]](#endnote-20)^,^[[21]](#endnote-21)^,^[[22]](#endnote-22)^^ are included in the review. We have already analysed 12, which were assessed as having risk of incorporation bias and circular reasoning. The remaining two are assessed here.

The first, Fung 2002^11^, identified cases of AHT using diagnostic codes. In the discussion they raise the concern of circular reasoning in the field of AHT stating that the “classification of the children as abused was on the basis of several criteria, including *no* *history accounting for patient’s serious head injury*, physical findings consistent only with abusive injuries (pattern, old and new lesions, location, etc). It is therefore not clear to what extent these conclusions are a self-fulfilling prophecy, that is, defining child abuse on the basis of subdural hemorrhage and retinal hemorrhage when there is ‘*no history* *accounting for patient’s serious head injury*,’ and then concluding that there is a high incidence of retinal hemorrhage in child abuse.”

Ruppel 2001^19^ provides no details on how AHT cases were classified, stating only that “patients with severe TBI who had a ventricular catheter placed were included in the study” and that “age, sex, mechanism of injury, initial GCS score, and Glasgow Outcome Score were recorded for all patients.” They report that excitatory amino acids (EAAs) are increased in AHT cases, but also note increases in patients who are younger and have poorer outcomes, both characteristics of cases classified as AHT. There is no way to determine whether elevated EAA values were independently associated with AHT, or whether they simply correlated with the characteristics used to diagnose AHT cases, i.e., incorporation bias.

**The Royal College Systematic Review in Head and Spinal Injuries 2019** is large. Here we focus on their citations regarding retinal haemorrhages and subdural haemorrhages.

We firstly look at “Clinical Question 2. What are the distinguishing clinical features of abusive head trauma in children?”, “2.2. Retinal Findings” where they cite 16 articles^^[[23]](#endnote-23)^,^[[24]](#endnote-24)^,^[[25]](#endnote-25)^,^[[26]](#endnote-26)^,^[[27]](#endnote-27)^,^[[28]](#endnote-28)^,^[[29]](#endnote-29)^,^[[30]](#endnote-30)^,^[[31]](#endnote-31)^,^[[32]](#endnote-32)^,^[[33]](#endnote-33)^,^[[34]](#endnote-34)^,^[[35]](#endnote-35)^,^[[36]](#endnote-36)^,^[[37]](#endnote-37)^,^[[38]](#endnote-38)^^ including one meta analysis and 1 systematic review. The only three studies that were not fully analysed in our analysis, either in the main body or an appendix, were the Maguire 2009 systematic review mentioned above and Ruppel 2001 and Fung 2002, also analysed above. So this Royal College Systematic Review essentially repeats the analysis of Maguire 2009, with regard to retinal hemorrhages.

Regarding “Clinical Question 3. What neuroradiological features distinguish abusive from non-abusive head trauma (nAHT)?” “3.1 Extra axial haemorrhages” “Subdural haemorrhages”, the Royal Society review cites 18 studies^^[[39]](#endnote-39)^,^[[40]](#endnote-40)^,^[[41]](#endnote-41)^,^[[42]](#endnote-42)^,^[[43]](#endnote-43)^,^[[44]](#endnote-44)^,^[[45]](#endnote-45)^,^[[46]](#endnote-46)^,^[[47]](#endnote-47)^,^[[48]](#endnote-48)^,^[[49]](#endnote-49)^,^[[50]](#endnote-50)^,^[[51]](#endnote-51)^,^[[52]](#endnote-52)^,^[[53]](#endnote-53)^,^[[54]](#endnote-54)^,^[[55]](#endnote-55)^,^[[56]](#endnote-56)^^ related to the “prevalence of SDH in children with AHT and nAHT”. Of these, we have already analysed 13, leaving five studies.

The first, Baerg et al. 2017 aimed to document the incidence and pattern of cervical spine (c-spine) injuries in children below 36 months with inflicted trauma. It selected cases with loss of consciousness and one intracranial finding, with SDH as one of the findings included. So the study did not attempt to determine whether SDH is specific to AHT, or diagnostic. It selected AHT cases where “the perpetrator confessed or the inflicted abuse or shaking was witnessed.” No details were provided as to whether the witnesses were independent, or the circumstances of the confessions, but it was stated that each case involved a “social services and law enforcement investigation”, which would only be launched if suspicion of AHT had arisen, presumably based on the medical findings.

The second, Buttram et al. 2015, compared lesion detection between CT and MRI after TBI. The AHT sample was based on “determination by the institution’s child forensic team investigation of caregiver interviews and clinical findings (ophthalmologic examination, radiographic imaging [skeletal survey, abdominal CT]…” Presumably the presence of SDH played a role in that determination.

The third, Gencturk 2019, classified cases as AHT “based on a legal conviction or confession”. No other details were provided, such as what fraction of cases involved confessions, circumstances surrounding the confessions, or whether SDH was a finding used to instigate investigations that led to a confession, or by experts as part of their diagnosis of AHT during the trial that led to conviction.

The fourth, Hymel et al. 2013, uses a similar set of predetermined criteria as Hymel 2019, as analysed in Appendix 3. In the 2013 article the criteria were 1. witnessed by an independent observer (1 case), 2. admission or confession (14 cases) , 3. caregiver denial of any head trauma (32), 4. caregiver account of the head injury event that was clearly historically inconsistent with repetition over time (19), 5. developmentally implausible explanations (12), and 6. the presence of two or more extracranial injuries that are considered suspicious for abuse as patterned bruising or intra-abdominal injury (53).

A positive point of Hymel 2013 is that the number of cases classified in each way is provided. The statistically dominant categories (3, 4 and 6) all require subjective assessments by experts. For example regarding category 3, are parents only repeatedly asked for explanations after doctors raise the suspicion of AHT based on the clinical findings?

Regarding category 4, why is a history of no trauma considered a “denial”? Data shows that cases where caregivers provide a history of no trauma have a strong tendency to not have signs of trauma, corroborating those histories^^[[57]](#endnote-57)^^. Clearly, just having a history of no trauma is not enough to diagnose AHT, so the description of this category is incomplete and therefore unscientific. If, as appears to be the case, clinical findings associated with AHT were used to conclude that the parents were lying, this would constitute incorporation bias.

Regarding category 6, do intra-abdominal injuries correlate with hypoxia, which is present in most AHT cases? Is bruising correlated with intracranial hemorrhages, for example through processes related to coagulopathy or genetic bleeding disorders?

The criteria used in Hymel 2013 have not been validated. How do the authors know that they are not merely finding correlations between the findings they use to identify AHT cases, and the findings they associate with those AHT cases?

The fifth, Thalayasingam et al. 2012, classified cases as AHT after a “work-out” by the Suspected Child Abuse and Neglect (SCAN) team. Following protocols, the SCAN team only assessed cases of head injury that were already suspected to be AHT, based on clinical findings, and their “work-out” also included clinical findings in making their assessment.

In summary, the Maquire et al. 2009 and the Royal Society systematic reviews rely heavily on studies that have high risk of incorporation bias and/or circular reasoning.

**Edwards et al. 2020** is a systematic review of confessed cases. 55 studies were included, containing 434 confessions. As quality control, they use the level of detail in the confession, which they claim “may be a reflection of the quality of the confession”. Using 4 quality levels, they ranked 82.7% of the cases in the lowest level, with only the mechanism of AHT described, whilst a further 10% of cases had the second lowest level. Only 2.7% of cases were considered to be of high quality. All 434 cases were included in their analysis regardless.

Moreover, false confessions *can* provide extensive detail^^[[58]](#endnote-58)^^, especially when interviewers inadvertently supply information to the accused. In one case of a detailed confession to AHT, a French parent said he falsely confessed in order for the infants to be returned to his wife, and that he took the details from what the investigators had told him during interviews, stating that “I just had to repeat what they had told me”^^[[59]](#endnote-59)^^. To evaluate a confession's quality, it is crucial to determine whether the details were volunteered by the accused or prompted by the interviewer. Specifically, in AHT cases, it is essential to establish who first suggested the mechanism of shaking, the accused or the interviewer. None of the studies included in Edwards et al. 2020 provide this critical information.

Further, 35% of “confessions” included in Edwards et al. 2020 were to revival shaking, i.e. the parent reported that the infant had collapsed, and that they had subsequently given it a shake in an attempt to revive it^^[[60]](#endnote-60)^^. These revival shaking histories were included as confessions. Other “confessions” included in Edwards et al. were to acts that also fall well short of being confessions to violent shaking, such as one father whose report that he “often bounced the infant on his knee after feeding, causing her head to move up and down and back and forth.”^^[[61]](#endnote-61)^^ was taken to be a confession and was included in the Edwards et al. 2020 data.

Edwards et al. also include confessions made during plea bargains, and confessions made in circumstances where children have been removed from the family home, both of these circumstances providing intense motives to provide a false confession. Further, pleas are only required in suspected AHT cases after medical assessment based on findings associated with AHT have resulted in charges being laid, and children are only removed after AHT is suspected or diagnosed based on medical findings. So findings in these cases clearly suffer incorporation bias and circular reasoning.

In summary, Edwards et al. 2020 make no attempt to address the issue of incorporation bias and circular reasoning that occurs^^[[62]](#endnote-62)^^ when confessions come after medical evaluation has already raised suspicion of abuse based on the clinical findings. As far as can be ascertained, no confessions included in Ewards et al. 2020 were made prior to the medical evaluation has already raised suspicion for AHT, based on medical findings.

**References**

1. Piteau SJ, Ward MG, Barrowman NJ, Plint AC. Clinical and radiographic characteristics associated with abusive and nonabusive head trauma: a systematic review. Pediatrics. 2012;130(2):315–323. doi: 10.1542/peds.2011-1545 [↑](#endnote-ref-1)
2. Kemp AM, Jaspan T, Grif ths J, et al. Neuroimaging: what neuroradiological features distinguish abusive from non-abusive head trauma? A systematic review. Arch Dis Child. 2011;96(12): 1103–1112. doi: 10.1136/archdischild-2011-300630 [↑](#endnote-ref-2)
3. Bhardwaj G, Chowdhury V, Jacobs MB, Moran KT, Martin FJ, Coroneo MT. A systematic review of the diagnostic accuracy of ocular signs in pediatric abusive head trauma. Ophthalmology. 2010;117(5):983–992.e17. [↑](#endnote-ref-3)
4. Maguire SA, Kemp AM, Lumb RC, Farewell DM. Estimating the probability of abusive head trauma: a pooled analysis. Pediatrics. 2011;128(3):e550–e64. doi: 10.1542/peds.2010-2949 [↑](#endnote-ref-4)
5. Hymel KP, Wang M, Chinchilli VM, et al. Estimating the probability of abusive head trauma after abuse evaluation. Child Abuse Negl. 2019;88:266–274. doi: 10.1016/j.chiabu.2018.11.015 [↑](#endnote-ref-5)
6. Maguire S, Pickerd N, Farewell D, Mann M, Tempest V, Kemp AM. Which clinical features distinguish inflicted from non-inflicted brain injury? A systematic review. Arch Dis Child. 2009;94(11):860–867. [↑](#endnote-ref-6)
7. Royal College of Paediatrics and Child Health, Cardiff University. Child Protection Evidence: Systematic Review on Head and Spinal Injuries. Cardiff University; August 2019.  [↑](#endnote-ref-7)
8. Edwards GA, Maguire SA, Gaither JR, Leventhal JM. What do confessions reveal about abusive head trauma? A systematic review.

   Child Abuse Rev. 2020;29(3):253–268.  [↑](#endnote-ref-8)
9. Hobbs C, Childs AM, Wynne J, et al. Subdural haematoma and effusion in infancy: an epidemiological study. Arch Dis Child 2005;90:952–5. [↑](#endnote-ref-9)
10. Kemp AM, Stoodley N, Cobley C, et al. Apnoea and brain swelling in non-accidental head injury. Arch Dis Child 2003;88:472–6. [↑](#endnote-ref-10)
11. Fung ELW, Sung RYT, Nelson EAS, et al. Unexplained subdural hematoma in young children: is it always child abuse? Pediatr Int 2002;44:37–42. [↑](#endnote-ref-11)
12. Bechtel K, Stoessel K, Leventhal JM, et al. Characteristics that distinguish accidental from abusive injury in hospitalized young children with head trauma. Pediatrics 2004;114:165–8. [↑](#endnote-ref-12)
13. Ettaro L, Berger RP, Songer T. Abusive head trauma in young children: characteristics and medical charges in a hospitalized population. Child Abuse Negl 2004;28:1099–111. [↑](#endnote-ref-13)
14. Ewing-Cobbs L, Kramer L, Prasad M, et al. Neuroimaging, physical, and developmental findings after inflicted and noninflicted traumatic brain injury in young children. Pediatrics 1998;102(2 Pt 1):300–7. [↑](#endnote-ref-14)
15. Hettler J, Greenes DS. Can the initial history predict whether a child with a head injury has been abused? Pediatrics 2003;111:602–7. [↑](#endnote-ref-15)
16. Hoskote A, Richards P, Anslow P, et al. Subdural haematoma and non-accidental head injury in children. Childs Nerv Syst 2002;18:311–17 [↑](#endnote-ref-16)
17. Keenan HT, Runyan DK, Marshall SW, et al. A population-based comparison of clinical and outcome characteristics of young children with serious inflicted and noninflicted traumatic brain injury. Pediatrics 2004;114:633–9. [↑](#endnote-ref-17)
18. Pierre-Kahn V, Roche O, Dureau P, et al. Ophthalmologic findings in suspected child abuse victims with subdural hematomas. Ophthalmology 2003;110:1718–23. [↑](#endnote-ref-18)
19. Ruppel RA, Kochanek PM, Adelson PD, et al. Excitatory amino acid concentrations in ventricular cerebrospinal fluid after severe traumatic brain injury in infants and children: the role of child abuse. J Pediatr 2001;138:18–25. [↑](#endnote-ref-19)
20. Shugerman RP, Paez A, Grossman DC, et al. Epidural hemorrhage: is it abuse? Pediatrics 1996;97:664–48. [↑](#endnote-ref-20)
21. Tzioumi D, Oates RK. Subdural hematomas in children under 2 years. Accidental or inflicted? A 10-year experience. Child Abuse Negl 1998;22:1105–12. [↑](#endnote-ref-21)
22. Vinchon M, Defoort-Dhellemmes S, Desurmont M, et al. Accidental and nonaccidental head injuries in infants: a prospective study. J Neurosurg 2005;102(4 Suppl):380–4. [↑](#endnote-ref-22)
23. Baerg J., Thirumoorthi A., Hazboun R., et al. Cervical spine injuries in young children: pattern and outcomes in accidental versus inflicted trauma. Journal of Surgical Research 2017; 219: 366-373. [↑](#endnote-ref-23)
24. Bhardwaj G., Jacobs M.B., Martin F.J., et al. Photographic assessment of retinal hemorrhages in infant head injury: the Childhood Hemorrhagic Retinopathy Study. Journal of Aapos: American Association for Pediatric Ophthalmology & Strabismus 2017; 21(1): 28-33.e22. [↑](#endnote-ref-24)
25. Hoskote A, et al. Subdural haematoma and non-accidental head injury in children. Childs Nerv Syst 2002;18:311–17 [↑](#endnote-ref-25)
26. Hettler J, Greenes DS. Can the initial history predict whether a child with a head injury has been abused? Pediatrics 2003;111:602–7. [↑](#endnote-ref-26)
27. Keenan HT, Runyan DK, Marshall SW, et al. A population-based comparison of clinical and outcome characteristics of young children with serious inflicted and noninflicted traumatic brain injury. Pediatrics 2004;114:633–9. [↑](#endnote-ref-27)
28. Bechtel K, Stoessel K, Leventhal JM, et al. Characteristics that distinguish accidental from abusive injury in hospitalized young children with head trauma. Pediatrics 2004;114:165–8. [↑](#endnote-ref-28)
29. Ewing-Cobbs L, Kramer L, Prasad M, et al. Neuroimaging, physical, and developmental findings after inflicted and noninflicted traumatic brain injury in young children. Pediatrics 1998;102(2 Pt 1):300–7. [↑](#endnote-ref-29)
30. Fung ELW, Sung RYT, Nelson EAS, et al. Unexplained subdural hematoma in young children: is it always child abuse? Pediatr Int 2002;44:37–42. [↑](#endnote-ref-30)
31. Hobbs C, Childs AM, Wynne J, et al. Subdural haematoma and effusion in infancy: an epidemiological study. Arch Dis Child 2005;90:952–5. [↑](#endnote-ref-31)
32. Kemp AM, Stoodley N, Cobley C, et al. Apnoea and brain swelling in non-accidental head injury. Arch Dis Child 2003;88:472–6. [↑](#endnote-ref-32)
33. Pierre-Kahn V, Roche O, Dureau P, et al. Ophthalmologic findings in suspected child abuse victims with subdural hematomas. Ophthalmology 2003;110:1718–23. [↑](#endnote-ref-33)
34. Ruppel RA, Kochanek PM, Adelson PD, et al. Excitatory amino acid concentrations in ventricular cerebrospinal fluid after severe traumatic brain injury in infants and children: the role of child abuse. J Pediatr 2001;138:18–25. [↑](#endnote-ref-34)
35. Shugerman RP, Paez A, Grossman DC, et al. Epidural hemorrhage: is it abuse? Pediatrics 1996;97:664–48. [↑](#endnote-ref-35)
36. Tzioumi D, Oates RK. Subdural hematomas in children under 2 years. Accidental or inflicted? A 10-year experience. Child Abuse Negl 1998;22:1105–12. [↑](#endnote-ref-36)
37. Vinchon M, Defoort-Dhellemmes S, Desurmont M, et al. Accidental and nonaccidental head injuries in infants: a prospective study. J Neurosurg 2005;102(4 Suppl):380–4. [↑](#endnote-ref-37)
38. Maguire S., Pickerd N., Farewell D., et al. Which clinical features distinguish inflicted from non-inflicted brain injury? A systematic review. Arch Dis Child 2009; 94(11): 860- 867. http://adc.bmj.com/content/archdischild/94/11/860.full.pdf [↑](#endnote-ref-38)
39. Baerg J., Thirumoorthi A., Hazboun R., et al. Cervical spine injuries in young children: pattern and outcomes in accidental versus inflicted trauma. Journal of Surgical Research 2017; 219: 366-373. [↑](#endnote-ref-39)
40. Bhardwaj G., Jacobs M.B., Martin F.J., et al. Photographic assessment of retinal hemorrhages in infant head injury: the Childhood Hemorrhagic Retinopathy Study. Journal of Aapos: American Association for Pediatric Ophthalmology & Strabismus 2017; 21(1): 28-33.e22. [↑](#endnote-ref-40)
41. Buttram S.D., Garcia-Filion P., Miller J., et al. Computed tomography vs magnetic resonance imaging for identifying acute lesions in pediatric traumatic brain injury. Hospital Pediatrics 2015; 5(2): 79-84. [↑](#endnote-ref-41)
42. Gencturk M, Tore HG, Nascene DR, Zhang L, Koksel Y, McKinney AM. Various Cranial and Orbital Imaging Findings in Pediatric Abusive and Non-abusive Head trauma, and Relation to Outcomes. Clin Neuroradiol. 2019 Jun;29(2):253-261. [↑](#endnote-ref-42)
43. Roach J.P., Acker S.N., Bensard D.D., et al. Head injury pattern in children can help differentiate accidental from non-accidental trauma. Pediatric Surgery International 2014; 30(11): 1103-1106 [↑](#endnote-ref-43)
44. Duhaime A.C., Alario A.J., Lewander W.J., et al. Head injury in very young children: mechanisms, injury types, and ophthalmologic findings in 100 hospitalized patients younger than 2 years of age. Pediatrics 1992; 90(2 Pt 1): 179-185. [↑](#endnote-ref-44)
45. Hettler J., Greenes D.S. Can the initial history predict whether a child with a head injury has been abused? Pediatrics 2003; 111(3): 602-607. [↑](#endnote-ref-45)
46. Keenan H.T., Runyan D.K., Marshall S.W., et al. A Population-Based Comparison of Clinical and Outcome Characteristics of Young Children With Serious Inflicted and Noninflicted Traumatic Brain Injury. Pediatrics 2004; 114(3): 633-639. [↑](#endnote-ref-46)
47. Bechtel K., Stoessel K., Leventhal J.M., et al. Characteristics that distinguish accidental from abusive injury in hospitalized young children with head trauma. Pediatrics 2004; 114(1): 165-168. [↑](#endnote-ref-47)
48. Vinchon M., Defoort-Dhellemmes S., Desurmont M., et al. Accidental and nonaccidental head injuries in infants: a prospective study. J Neurosurg 2005; 102(4 Suppl): 380-384. [↑](#endnote-ref-48)
49. Ettaro L., Berger R.P., Songer T. Abusive head trauma in young children: characteristics and medical charges in a hospitalized population. Child Abuse Negl 2004; 28(10): 1099- 1111 [↑](#endnote-ref-49)
50. Adamo M.A., Drazin D., Smith C., et al. Comparison of accidental and nonaccidental traumatic brain injuries in infants and toddlers: demographics, neurosurgical interventions, and outcomes. J Neurosurg Pediatr 2009; 4(5): 414-419. [↑](#endnote-ref-50)
51. Hymel K.P., Willson D.F., Boos S.C., et al. Derivation of a clinical prediction rule for pediatric abusive head trauma. Pediatr Crit Care Med 2013; 14(2): 210-220. [↑](#endnote-ref-51)
52. Goldstein B., Kelly M.M., Bruton D., et al. Inflicted versus accidental head injury in critically injured children. Crit Care Med 1993; 21(9): 1328-1332. [↑](#endnote-ref-52)
53. Hymel K.P., Rumack C.M., Hay T.C., et al. Comparison of intracranial computed tomographic (CT) findings in pediatric abusive and accidental head trauma. Pediatr Radiol 1997; 27(9): 743-747 [↑](#endnote-ref-53)
54. Reece R.M., Sege R. Childhood head injuries: accidental or inflicted? Arch Pediatr Adolesc Med 2000; 154(1): 11-15. [↑](#endnote-ref-54)
55. Thalayasingam M., Veerakumarasivam A., Kulanthayan S., et al. Clinical clues for head injuries amongst Malaysian infants: accidental or non-accidental? Injury 2012; 43(12): 2083-2087 [↑](#endnote-ref-55)
56. Vinchon M., de Foort-Dhellemmes S., Desurmont M., et al. Confessed abuse versus witnessed accidents in infants: comparison of clinical, radiological, and ophthalmological data in corroborated cases. Childs Nerv Syst 2010; 26(5): 637-645. [↑](#endnote-ref-56)
57. Brook, C.B. (2023), Data-driven evidence shows truthful caregiver histories and significant overdiagnosis of abusive head trauma. Ann Child Neurol Soc, 1: 299-304. <https://doi.org/10.1002/cns3.20035> [↑](#endnote-ref-57)
58. Garrett BL. The substance of false confessions, 62. *Stanford Law Rev*. 2010;62: 1051-1118. [↑](#endnote-ref-58)
59. Rossant C, Brook C. Why admitted cases of AHT make a low quality reference standard: A survey of people accused of AHT in France. Forensic Sci Int Synerg. 2022 Dec 29;6:100312 [↑](#endnote-ref-59)
60. Lynøe, N. & Eriksson, A. (2022) Circular reasoning, confessions and abusive head trauma: A critical analysis of Edwards et al. (2020). *Child Abuse Review*, 31(6), e2777. Available from: [**https://doi.org/10.1002/car.2777**](https://doi.org/10.1002/car.2777) [↑](#endnote-ref-60)
61. D. Biron, D. Shelton, Perpetrator accounts in infant abusive head trauma brought about by a shaking event, Child Abuse Negl. 29 (2005) 1347–1358, https://doi.org/10.1016/j.chiabu.2005.05.003. [↑](#endnote-ref-61)
62. Brook C. Evidence for significant misdiagnosis of abusive head trauma in pediBIRN data. Forensic Sci Int Synerg. 2023 Jan 10;6:100314. [↑](#endnote-ref-62)
