# Supplementary material for: A critical review of the American Academy of Pediatrics technical report on abusive head trauma
Source: Forensic Sci Int Synerg. 2025 Dec 3;11:100650. doi: 10.1016/j.fsisyn.2025.100650 (PMC12721060; doi:10.1016/j.fsisyn.2025.100650)
Supplement: Multimedia component 5 [file mmc5.pdf]

Study Methodology

Reference Number

70

Year

2011

Authors

Bennett et al.

Correct Study? (check the reference number, the first number of the filename, against the reference number in AAP_refs.pdf)

- (•) Yes
- ( ) No

Classification Methods

- [x] MDTs/Experts
- [ ] Diagnostic code/medical records
- [ ] Predetermined Criteria
- [ ] Admission/Confession
- [ ] Conviction/Court Confirmation
- [ ] Witnessed
- [ ] Other:

Comment on Methodology

Describe how the study creates a sample of AHT cases

A survey of paediatricians and paediatric subspecialists who reported whether the case was a "sustained a head or brain injury consistentv with suspected child maltreatment and the injury was reported to a provincial or territorial child welfare agency." The main aim was to estimate of the rate of head injury secondary to suspected child maltreatment in Canada but does give tables of findings in AHT cases, which have high risk of incorporation bias and circular reasoning.

Has the study adequately addressed the risk of incorporation bias and/or circular reasoning?

- [ ] Yes
- [x] No

This form was created inside Universidad de La Laguna.

Google Forms

Study Methodology

Reference Number

164

Year

2016

Authors

Payne et al.

Correct Study? (check the reference number, the first number of the filename, against the reference number in AAP_refs.pdf)

- (●) Yes
- ( ) No

Classification Methods

- [x] MDTs/Experts
- [ ] Diagnostic code/medical records
- [ ] Predetermined Criteria
- [ ] Admission/Confession
- [ ] Conviction/Court Confirmation
- [ ] Witnessed
- [ ] Other:

Comment on Methodology

Describe how the study creates a sample of AHT cases

Infants with long bone injuries were found to not have RHs. Abused cases were those "considered by a child abuse specialist to be concerning for abuse."

Has the study adequately addressed the risk of incorporation bias and/or circular reasoning?

- [ ] Yes
- [x] No

This form was created inside Universidad de La Laguna.

Google Forms

Study Methodology

Reference Number

540

Year

2009

Authors

Gill et al.

Correct Study? (check the reference number, the first number of the filename, against the reference number in AAP_refs.pdf)

- ○ Yes
- ○ No

Classification Methods

- [ ] MDTs/Experts
- [x] Diagnostic code/medical records
- [ ] Predetermined Criteria
- [ ] Admission/Confession
- [ ] Conviction/Court Confirmation
- [ ] Witnessed
- [ ] Other:

Comment on Methodology

Describe how the study creates a sample of AHT cases

Records from the Office of Chief Medical Examiner were used retrospectively.

Has the study adequately addressed the risk of incorporation bias and/or circular reasoning?

- [ ] Yes
- [x] No

This form was created inside Universidad de La Laguna.

Google Forms

Study Methodology

Reference Number

117

Year

2002

Authors

Biousse et al.

Correct Study? (check the reference number, the first number of the filename, against the reference number in AAP_refs.pdf)

- (●) Yes
- ( ) No

Classification Methods

- [x] MDTs/Experts
- [ ] Diagnostic code/medical records
- [ ] Predetermined Criteria
- [ ] Admission/Confession
- [ ] Conviction/Court Confirmation
- [ ] Witnessed
- [ ] Other:

Comment on Methodology

Describe how the study creates a sample of AHT cases

AHT cases were "suspected or confirmed SBS (including extensive evaluation to rule-out medical conditions that can mimic SBS, skeletal survey, ocular evaluation, evaluation of patients and their families by the pediatric social service team and by a pediatrician from the Children's Protection Program"

Has the study adequately addressed the risk of incorporation bias and/or circular reasoning?

- [ ] Yes
- [x] No

This form was created inside Universidad de La Laguna.

Google Forms

Study Methodology

Reference Number

101

Year

2000

Authors

EwingCobbs et al.

Correct Study? (check the reference number, the first number of the filename, against the reference number in AAP_refs.pdf)

- (•) Yes
- ( ) No

Classification Methods

- [x] MDTs/Experts
- [ ] Diagnostic code/medical records
- [ ] Predetermined Criteria
- [ ] Admission/Confession
- [ ] Conviction/Court Confirmation
- [ ] Witnessed
- [ ] Other:

Comment on Methodology

Describe how the study creates a sample of AHT cases

Determination of inflicted injury was based on the assessment of the state protective agency and the children's protection committee at each hospital. Injuries incompatible with the stated mechanism of injury based on epidemiologic studies of consequences of falls (e.g. bilateral subdural and retinal hemorrhages attributed to falling off a couch; multiple skull fractures, multiple intracranial hemorrhages, and bilateral cephalohematomas resulting from a reported 4-foot fall), and unexplained injuries (e.g. no history of trauma with intracranial injuries and old skeletal fractures) were regarded as indicative of assault. Additionally, delay in seeking treatment and changing history were also considered to be possible indicators of inflicted injuries.

Has the study adequately addressed the risk of incorporation bias and/or circular reasoning?

- [ ] Yes
- [x] No

This form was created inside Universidad de La Laguna.

Google Forms

Study Methodology

Reference Number

214

Year

2014

Authors

Pontarelli et al.

Correct Study? (check the reference number, the first number of the filename, against the reference number in AAP_refs.pdf)

- (●) Yes
- ( ) No

Classification Methods

- [x] MDTs/Experts
- [ ] Diagnostic code/medical records
- [ ] Predetermined Criteria
- [ ] Admission/Confession
- [ ] Conviction/Court Confirmation
- [ ] Witnessed
- [ ] Other:

Comment on Methodology

Describe how the study creates a sample of AHT cases

"Nonaccidental trauma was determined after evaluation by the institutional child abuse team and the state Department of Human Services. "

Has the study adequately addressed the risk of incorporation bias and/or circular reasoning?

- [ ] Yes
- [x] No

This form was created inside Universidad de La Laguna.

Google Forms

Study Methodology

Reference Number

178

Year

2008

Authors

Watts and Obi

Correct Study? (check the reference number, the first number of the filename, against the reference number in AAP_refs.pdf)

- (●) Yes
- ( ) No

Classification Methods

- [ ] MDTs/Experts
- [x] Diagnostic code/medical records
- [ ] Predetermined Criteria
- [ ] Admission/Confession
- [ ] Conviction/Court Confirmation
- [ ] Witnessed
- [ ] Other:

Comment on Methodology

Describe how the study creates a sample of AHT cases

Single AHT case, taken from child protection registry.

Has the study adequately addressed the risk of incorporation bias and/or circular reasoning?

- [ ] Yes
- [x] No

This form was created inside Universidad de La Laguna.

Google Forms

Study Methodology

Reference Number

125

Year

1992

Authors

Jaspan et al.

Correct Study? (check the reference number, the first number of the filename, against the reference number in AAP_refs.pdf)

- (●) Yes
- ( ) No

Classification Methods

- [ ] MDTs/Experts
- [x] Diagnostic code/medical records
- [ ] Predetermined Criteria
- [x] Admission/Confession
- [ ] Conviction/Court Confirmation
- [ ] Witnessed
- [ ] Other:

Comment on Methodology

Describe how the study creates a sample of AHT cases

6 case series. 1 case had admissions by the boyfriend of the mother. 5 cases had been "referred to the regional paediatric neurosurgeon as cases of suspected child abuse "

Has the study adequately addressed the risk of incorporation bias and/or circular reasoning?

- [ ] Yes
- [x] No

This form was created inside Universidad de La Laguna.

Google Forms

Study Methodology

Reference Number

113

Year

2015

Authors

Hahnemann et al.

Correct Study? (check the reference number, the first number of the filename, against the reference number in AAP_refs.pdf)

- ○ Yes
- ○ No

Classification Methods

- [x] MDTs/Experts
- [ ] Diagnostic code/medical records
- [ ] Predetermined Criteria
- [ ] Admission/Confession
- [ ] Conviction/Court Confirmation
- [ ] Witnessed
- [ ] Other:

Comment on Methodology

Describe how the study creates a sample of AHT cases

Cases taken from the archives of the Institute of Legal Medicine (ILM). "Medicolegal expert opinions represent the most objective and best reference standard for AHT in Germany"

Has the study adequately addressed the risk of incorporation bias and/or circular reasoning?

- [ ] Yes
- [x] No

This form was created inside Universidad de La Laguna.

Google Forms

Study Methodology

Reference Number

447

Year

2007

Authors

Altman et al.

Correct Study? (check the reference number, the first number of the filename, against the reference number in AAP_refs.pdf)

- ○ Yes
- ○ No

Classification Methods

- [ ] MDTs/Experts
- [ ] Diagnostic code/medical records
- [x] Predetermined Criteria
- [ ] Admission/Confession
- [ ] Conviction/Court Confirmation
- [ ] Witnessed
- [ ] Other:

Comment on Methodology

Describe how the study creates a sample of AHT cases

"evidence of an intracranial hemorrhage that could not be explained by an accident or nontraumatic process and that was accompanied by other supportive findings consistent with child abuse. Those supportive findings included unexplained fractures involving ribs, long bones, or skull; unexplained abrasions or ecchymoses of the head, face, neck, or chest; and retinal hemorrhage or detachment"

Has the study adequately addressed the risk of incorporation bias and/or circular reasoning?

- [ ] Yes
- [x] No

This form was created inside Universidad de La Laguna.

Google Forms

Study Methodology

Reference Number

38

Year

2020

Authors

Hymel et al.

Correct Study? (check the reference number, the first number of the filename, against the reference number in AAP_refs.pdf)

- (selected) Yes
- ( ) No

Classification Methods

- [ ] MDTs/Experts
- [ ] Diagnostic code/medical records
- [x] Predetermined Criteria
- [ ] Admission/Confession
- [ ] Conviction/Court Confirmation
- [ ] Witnessed
- [ ] Other: ______

Comment on Methodology

Describe how the study creates a sample of AHT cases

Classification. as "definite" AHT: "Eligible patients with intracranial hemorrhage AND one or more of the following: retinal findings consistent with abuse, high-specificity skin findings, OR moderate or high-specificity fractures."

Has the study adequately addressed the risk of incorporation bias and/or circular reasoning?

- [ ] Yes
- [x] No

This form was created inside Universidad de La Laguna.

Google Forms

Study Methodology

Reference Number

56

Year

2005

Authors

Arbogast et al.

Correct Study? (check the reference number, the first number of the filename, against the reference number in AAP_refs.pdf)

- (●) Yes
- ( ) No

Classification Methods

- [ ] MDTs/Experts
- [x] Diagnostic code/medical records
- [ ] Predetermined Criteria
- [ ] Admission/Confession
- [ ] Conviction/Court Confirmation
- [ ] Witnessed
- [ ] Other:

Comment on Methodology

Describe how the study creates a sample of AHT cases

Data came from "a statewide registry of clinical data from all 26 accredited trauma centers (adult and pediatric) throughout the state of Pennsylvania. "

Has the study adequately addressed the risk of incorporation bias and/or circular reasoning?

- [ ] Yes
- [x] No

This form was created inside Universidad de La Laguna.

Google Forms

Study Methodology

Reference Number

596

Year

1997

Authors

Feldman et al.

Correct Study? (check the reference number, the first number of the filename, against the reference number in AAP_refs.pdf)

- (●) Yes
- ( ) No

Classification Methods

- [x] MDTs/Experts
- [ ] Diagnostic code/medical records
- [ ] Predetermined Criteria
- [ ] Admission/Confession
- [ ] Conviction/Court Confirmation
- [ ] Witnessed
- [ ] Other:

Comment on Methodology

Describe how the study creates a sample of AHT cases

Inclusion as a AHT case required "a positive diagnosis of inflicted head injury"

Has the study adequately addressed the risk of incorporation bias and/or circular reasoning?

- [ ] Yes
- [x] No

This form was created inside Universidad de La Laguna.

Google Forms

Study Methodology

Reference Number

467

Year

2019

Authors

Hymel et al.

Correct Study? (check the reference number, the first number of the filename, against the reference number in AAP_refs.pdf)

- (•) Yes
- ( ) No

Classification Methods

- [x] MDTs/Experts
- [ ] Diagnostic code/medical records
- [x] Predetermined Criteria
- [x] Admission/Confession
- [ ] Conviction/Court Confirmation
- [x] Witnessed
- [ ] Other: ____________

Comment on Methodology

Describe how the study creates a sample of AHT cases

Two methods were used independently: one was the physician's final diagnosis (which carries a high risk of incorporation bias), and the other was a set of predetermined criteria.

These criteria included witnessed cases; however, the number of such cases was very small and statistically insignificant. Another criterion was caregiver admissions (i.e., confessions), but no details are provided about the nature of these admissions — specifically, whether they were made spontaneously or after medical findings led to interrogation. As discussed in the main text, this raises a serious risk of incorporation bias.

Another criterion was that the caregiver's history did not include trauma. This group represents the largest subset of cases classified as AHT. In these cases, the caregiver's report is labeled a "denial" — not because of any external evidence contradicting it, but because it conflicts with the medical belief that certain present findings are indicative of AHT. If those findings were absent, the same caregiver account would not be questioned or labeled a denial. In other words, the assumption that denial is a marker of abuse only arises in the presence of findings already believed to signify AHT. This creates a circular logic: the medical findings generate suspicion, and the absence of a reported trauma is then interpreted as further confirmation of abus. This constitutes a clear risk of incorporation bias and circular reasoning.

Another criterion involved assessing inconsistencies in caregiver accounts over time, but no specific details are given. For instance, did a physician — based on findings commonly associated with AHT — dismiss the initial caregiver explanation and prompt the suggestion of an alternative? This introduces a string risk of incorporation bias and circular reasoning.
The criterion that the caregiver's account was developmentally inconsistent with the child's motor abilities is also problematic due to a lack of context. No information is provided about what medical findings triggered the caregiver's questioning. Were they interrogated because medical findings already suggested AHT? The assumption of findings are traumatic is also implicit in this criterion, as is the assumption of what types of accidents can lead to findings associated with abuse. There is strong risk of incorporation bias in this criterion.

Finally, another criterion was the presence of dry contact burns, hot water immersion burns, or CT-confirmed intra-abdominal injury. However, such cases are statistically rare in the dataset and their connection to AHT are not established.

Has the study adequately addressed the risk of incorporation bias and/or circular reasoning?

- [ ] Yes
- [x] No

This form was created inside Universidad de La Laguna.

Study Methodology

Reference Number

600

Year

2014

Authors

Foster et al.

Correct Study? (check the reference number, the first number of the filename, against the reference number in AAP_refs.pdf)

- Yes
- No

Classification Methods

- [x] MDTs/Experts
- [ ] Diagnostic code/medical records
- [ ] Predetermined Criteria
- [ ] Admission/Confession
- [ ] Conviction/Court Confirmation
- [ ] Witnessed
- [ ] Other:

Comment on Methodology

Describe how the study creates a sample of AHT cases

Diagnosis of child abuse "by the Child Advocacy Center of the University of Pittsburgh"

Has the study adequately addressed the risk of incorporation bias and/or circular reasoning?

- [ ] Yes
- [x] No

This form was created inside Universidad de La Laguna.

Google Forms

Study Methodology

Reference Number

519

Year

1998

Authors

Shannon et al.

Correct Study? (check the reference number, the first number of the filename, against the reference number in AAP_refs.pdf)

- (selected) Yes
- ( ) No

Classification Methods

- [ ] MDTs/Experts
- [ ] Diagnostic code/medical records
- [ ] Predetermined Criteria
- [x] Admission/Confession
- [x] Conviction/Court Confirmation
- [x] Witnessed
- [ ] Other:

Comment on Methodology

Describe how the study creates a sample of AHT cases

They "searched the forensic autopsy records at our institution over the last 15 years in conjunction with the files of the office of the Chief Coroner of Ontario to identify cases of fatal SBS. In every case included there was either a witnessed or confessed episode of
shaking prior to presentation, or a coroners verdict of child abuse."

No details provided on witnesses of confessions. No mention of how many cases were witnessed nor how many confessed, compared to how many were categorised due to coroners verdict.

Has the study adequately addressed the risk of incorporation bias and/or circular reasoning?

- [ ] Yes
- [x] No

This form was created inside Universidad de La Laguna.

Google Forms

Study Methodology

Reference Number

99

Year

2007

Authors

Myhre et al.

Correct Study? (check the reference number, the first number of the filename, against the reference number in AAP_refs.pdf)

- (●) Yes
- ( ) No

Classification Methods

- [x] MDTs/Experts
- [x] Diagnostic code/medical records
- [ ] Predetermined Criteria
- [ ] Admission/Confession
- [ ] Conviction/Court Confirmation
- [ ] Witnessed
- [ ] Other:

Comment on Methodology

Describe how the study creates a sample of AHT cases

Classified cases as AHT if they were documented as abuse in the medical record and referred to child protective services, or based on the authors' own judgment having assessed additional criteria such as "injuries where the medical history could not explain the injury"—a determination that necessarily relies on prior assumptions about the causes of accidental versus abusive trauma. Similarly, classification as accidental included the criterion "injuries evaluated as accidental in the medical record," again incorporating prior assumptions. Given the widespread belief in an association between SDH and AHT, the study carries a high risk of incorporation bias and circular reasoning when used to compare what injuries are caused by accidental versus abusive trauma.

Has the study adequately addressed the risk of incorporation bias and/or circular reasoning?

- [ ] Yes
- [x] No

This form was created inside Universidad de La Laguna.

Google Forms

Study Methodology

Reference Number

124

Year

2016

Authors

Palifka et al.

Correct Study? (check the reference number, the first number of the filename, against the reference number in AAP_refs.pdf)

- (•) Yes
- ( ) No

Classification Methods

- [x] MDTs/Experts
- [ ] Diagnostic code/medical records
- [ ] Predetermined Criteria
- [x] Admission/Confession
- [ ] Conviction/Court Confirmation
- [ ] Witnessed
- [ ] Other:

Comment on Methodology

Describe how the study creates a sample of AHT cases

"291 patients who either by confession and/or evaluation by the institutional child abuse medical evaluation team were determined to have inflicted trauma." No other details provided.

Has the study adequately addressed the risk of incorporation bias and/or circular reasoning?

- [ ] Yes
- [x] No

This form was created inside Universidad de La Laguna.

Google Forms

Study Methodology

Reference Number

548

Year

1998

Authors

Atwal et al.

Correct Study? (check the reference number, the first number of the filename, against the reference number in AAP_refs.pdf)

- (selected) Yes
- ( ) No

Classification Methods

- [ ] MDTs/Experts
- [x] Diagnostic code/medical records
- [ ] Predetermined Criteria
- [ ] Admission/Confession
- [ ] Conviction/Court Confirmation
- [ ] Witnessed
- [ ] Other:

Comment on Methodology

Describe how the study creates a sample of AHT cases

"24 cases of fatal non-accidental head injury in children were retrieved from the archival records of the Department of Forensic Pathology, University of Sheffield from 1990–1996."

Has the study adequately addressed the risk of incorporation bias and/or circular reasoning?

- [ ] Yes
- [x] No

This form was created inside Universidad de La Laguna.

Google Forms

Study Methodology

Reference Number

26

Year

2018

Authors

Young et al.

Correct Study? (check the reference number, the first number of the filename, against the reference number in AAP_refs.pdf)

- (●) Yes
- ( ) No

Classification Methods

- [x] MDTs/Experts
- [ ] Diagnostic code/medical records
- [ ] Predetermined Criteria
- [ ] Admission/Confession
- [ ] Conviction/Court Confirmation
- [ ] Witnessed
- [ ] Other:

Comment on Methodology

Describe how the study creates a sample of AHT cases

A nine-member panel of multidisciplinary injury experts including four child abuse pediatricians, four pediatric emergency medicine physicians, and a bioengineer subsequently reviewed the history of injury, physical examination, and test results of each de-identified subject. This compilation of reviewed data was referred to as the subject's "case" file. Using pre-defined criteria, each case was categorized as clinically determined abuse , clinically determined accident , or indeterminate by members of the MEP.

No information was provided as to what were the predetermined criteria, making it a "black box" MDT determination.

Has the study adequately addressed the risk of incorporation bias and/or circular reasoning?

- [ ] Yes
- [x] No

This form was created inside Universidad de La Laguna.

Google Forms

Study Methodology

Reference Number

337

Year

2017

Authors

Berger et al.

Correct Study? (check the reference number, the first number of the filename, against the reference number in AAP_refs.pdf)

- (●) Yes
- ( ) No

Classification Methods

- [x] MDTs/Experts
- [ ] Diagnostic code/medical records
- [ ] Predetermined Criteria
- [ ] Admission/Confession
- [ ] Conviction/Court Confirmation
- [ ] Witnessed
- [ ] Other:

Comment on Methodology

Describe how the study creates a sample of AHT cases

"Abusive head trauma was defined as acute ICH and the assessment of probable or definite abuse by each site’s hospital-based Child Protection Team."

Has the study adequately addressed the risk of incorporation bias and/or circular reasoning?

- [ ] Yes
- [x] No

This form was created inside Universidad de La Laguna.

Google Forms

Study Methodology

Reference Number

145

Year

2000

Authors

McCabe and Donahue

Correct Study? (check the reference number, the first number of the filename, against the reference number in AAP_refs.pdf)

- ◯ Yes
- ◯ No

Classification Methods

- [x] MDTs/Experts
- [ ] Diagnostic code/medical records
- [ ] Predetermined Criteria
- [ ] Admission/Confession
- [ ] Conviction/Court Confirmation
- [ ] Witnessed
- [ ] Other:

Comment on Methodology

Describe how the study creates a sample of AHT cases

"A diagnosis of SBS was considered when bilateral retinal hemorrhages were observed in a situation where the injury was not consistent with the history, when other obvious signs of abuse were present, or if there had been a history of a previous suspicious episode in a lethargic infant."

Has the study adequately addressed the risk of incorporation bias and/or circular reasoning?

- [ ] Yes
- [x] No

This form was created inside Universidad de La Laguna.

Google Forms

Study Methodology

Reference Number

106

Year

2002

Authors

Wells et al.

Correct Study? (check the reference number, the first number of the filename, against the reference number in AAP_refs.pdf)

- (●) Yes
- ( ) No

Classification Methods

- [x] MDTs/Experts
- [ ] Diagnostic code/medical records
- [ ] Predetermined Criteria
- [x] Admission/Confession
- [ ] Conviction/Court Confirmation
- [ ] Witnessed
- [ ] Other:

Comment on Methodology

Describe how the study creates a sample of AHT cases

"We considered an injury to be unintentional if it was witnessed by someone other than the caretaker or there were no discrepancies between the described mechanism and the physical findings. We additionally categorized the unintentional injury group according to whether the injury was caused by a fall, motor vehicle collision, a fall in an infant walker, or by being struck by an object.

We considered an injury to be intentional if there was a confession of abuse, the injuries were incompatible with the stated mechanism of injury, or the caretaker offered no explanation for the injuries."

Has the study adequately addressed the risk of incorporation bias and/or circular reasoning?

- [ ] Yes
- [x] No

This form was created inside Universidad de La Laguna.

Google Forms

Study Methodology

Reference Number

114

Year

2007

Authors

Zimmerman et al.

Correct Study? (check the reference number, the first number of the filename, against the reference number in AAP_refs.pdf)

- (●) Yes
- ( ) No

Classification Methods

- [ ] MDTs/Experts
- [ ] Diagnostic code/medical records
- [ ] Predetermined Criteria
- [ ] Admission/Confession
- [ ] Conviction/Court Confirmation
- [ ] Witnessed
- [x] Other: No selection method was described.

Comment on Methodology

Describe how the study creates a sample of AHT cases

"This paper looks at the patterns of DWI abnormalities in non-accidental trauma (NAT) in infants and young children who have been the victims of abuse." No mention is made of where the cases came from or how they were classified as AHT.

Has the study adequately addressed the risk of incorporation bias and/or circular reasoning?

- [ ] Yes
- [x] No

This form was created inside Universidad de La Laguna.

Google Forms

Study Methodology

Reference Number

21

Year

1999

Authors

Jenny

Correct Study? (check the reference number, the first number of the filename, against the reference number in AAP_refs.pdf)

- (●) Yes
- ( ) No

Classification Methods

- [x] MDTs/Experts
- [ ] Diagnostic code/medical records
- [ ] Predetermined Criteria
- [ ] Admission/Confession
- [ ] Conviction/Court Confirmation
- [ ] Witnessed
- [ ] Other:

Comment on Methodology

Describe how the study creates a sample of AHT cases

"Confirmation that head trauma was inflicted requires multidisciplinary team consensus."

Has the study adequately addressed the risk of incorporation bias and/or circular reasoning?

- [ ] Yes
- [x] No

This form was created inside Universidad de La Laguna.

Google Forms

Study Methodology

Reference Number

527

Year

2011

Authors

Johnson et al.

Correct Study? (check the reference number, the first number of the filename, against the reference number in AAP_refs.pdf)

- (●) Yes
- ( ) No

Classification Methods

- [ ] MDTs/Experts
- [x] Diagnostic code/medical records
- [ ] Predetermined Criteria
- [ ] Admission/Confession
- [ ] Conviction/Court Confirmation
- [ ] Witnessed
- [ ] Other:

Comment on Methodology

Describe how the study creates a sample of AHT cases

"Deaths in children under the age of 3, in the 1999 calendar year, were identified by electronic search of records of the Office of the Chief Medical Examiner (OCME) for the State of Maryland for cause of death, manner of death, and the presence of head injuries
demonstrated at autopsy. "

Has the study adequately addressed the risk of incorporation bias and/or circular reasoning?

- [ ] Yes
- [x] No

This form was created inside Universidad de La Laguna.

Google Forms

Study Methodology

Reference Number

227

Year

2010

Authors

Hymel et al.

Correct Study? (check the reference number, the first number of the filename, against the reference number in AAP_refs.pdf)

- (●) Yes
- ( ) No

Classification Methods

- [x] MDTs/Experts
- [ ] Diagnostic code/medical records
- [x] Predetermined Criteria
- [x] Admission/Confession
- [ ] Conviction/Court Confirmation
- [x] Witnessed
- [ ] Other:

Comment on Methodology

Describe how the study creates a sample of AHT cases

"Cases in which primary caregiver admitted abusive acts that resulted in acute clinical signs linked to child's acute traumatic cranial injuries
Cases in which primary caregiver's abusive acts were witnessed independently and resulted in acute clinical signs linked to child's acute traumatic cranial injuries
Cases in which child not yet cruising or walking first manifested acute clinical signs linked to acute traumatic cranial injuries while in the care of primary caregiver who specifically denied that child had experienced any trauma
Cases in which primary caregiver provided explanation for child's acute clinical signs and acute traumatic cranial injuries that was clearly
developmentally inconsistent with parents' description of child's developmental capabilities
Cases in which primary caregiver provided explanation for child's acute clinical signs and acute traumatic cranial injuries that was clearly
historically inconsistent with repetition over time
Cases in which child with acute head injury demonstrated 2 categories of noncranial injuries considered moderately or highly specific for abuse"

Has the study adequately addressed the risk of incorporation bias and/or circular reasoning?

- [ ] Yes
- [x] No

This form was created inside Universidad de La Laguna.

Google Forms

Study Methodology

Reference Number

343

Year

2015

Authors

Parisi et al.

Correct Study? (check the reference number, the first number of the filename, against the reference number in AAP_refs.pdf)

- (•) Yes
- ( ) No

Classification Methods

- [x] MDTs/Experts
- [ ] Diagnostic code/medical records
- [ ] Predetermined Criteria
- [ ] Admission/Confession
- [ ] Conviction/Court Confirmation
- [ ] Witnessed
- [ ] Other:

Comment on Methodology

Describe how the study creates a sample of AHT cases

"Ten children were identified through clinical child abuse consultation at the time of their diagnostic evaluations at either Seattle Children's or Harborview Medical Center in Seattle, Washington. Two cases arose from medical-legal consultations."

Has the study adequately addressed the risk of incorporation bias and/or circular reasoning?

- [ ] Yes
- [x] No

This form was created inside Universidad de La Laguna.

Google Forms

Study Methodology

Reference Number

111

Year

2018

Authors

Ronning et al.

Correct Study? (check the reference number, the first number of the filename, against the reference number in AAP_refs.pdf)

- (•) Yes
- ( ) No

Classification Methods

- [x] MDTs/Experts
- [ ] Diagnostic code/medical records
- [ ] Predetermined Criteria
- [ ] Admission/Confession
- [ ] Conviction/Court Confirmation
- [ ] Witnessed
- [ ] Other:

Comment on Methodology

Describe how the study creates a sample of AHT cases

A diagnosis of AHT was established by the consensus of an MDT (CAPs +pediatr. neurosurgeons+ped. radiologists+ped. ophthalmologists+ped. nurse practitioners and social workers. “A definition of abuse was based upon the outcome of a legal or multi-agency child protection investigation or stated criteria where diagnosis was based upon
additional features and did not rely on the neuroradiological findings, to minimize the risk of circularity..."

Has the study adequately addressed the risk of incorporation bias and/or circular reasoning?

- [ ] Yes
- [x] No

This form was created inside Universidad de La Laguna.

Google Forms

Study Methodology

Reference Number

436

Year

2018

Authors

Hansen et al.

Correct Study? (check the reference number, the first number of the filename, against the reference number in AAP_refs.pdf)

- (●) Yes
- ( ) No

Classification Methods

- [ ] MDTs/Experts
- [ ] Diagnostic code/medical records
- [x] Predetermined Criteria
- [ ] Admission/Confession
- [ ] Conviction/Court Confirmation
- [ ] Witnessed
- [ ] Other:

Comment on Methodology

Describe how the study creates a sample of AHT cases

"AHT" was not mentioned, but groups of "SDH-mild symptoms" and "SDH-severe symptoms" and "BESS") were compared regarding "concomitant suspicious injuries" (CSIs). RH and fx were *always* considered as "CSIs".

Has the study adequately addressed the risk of incorporation bias and/or circular reasoning?

- [ ] Yes
- [x] No

This form was created inside Universidad de La Laguna.

Google Forms

Study Methodology

Reference Number

376

Year

2003

Authors

Morad et al.

Correct Study? (check the reference number, the first number of the filename, against the reference number in AAP_refs.pdf)

- (selected) Yes
- ( ) No

Classification Methods

- [ ] MDTs/Experts
- [ ] Diagnostic code/medical records
- [x] Predetermined Criteria
- [ ] Admission/Confession
- [ ] Conviction/Court Confirmation
- [ ] Witnessed
- [ ] Other:

Comment on Methodology

Describe how the study creates a sample of AHT cases

Diagnosis required at least two of the following: characteristic neuroradiologic abnormalities; skeletal injury; RHs; a history of child abuse that included shaking; and no adequate history to explain the injuries

Has the study adequately addressed the risk of incorporation bias and/or circular reasoning?

- [ ] Yes
- [x] No

This form was created inside Universidad de La Laguna.

Google Forms

Study Methodology

Reference Number

638

Year

2023

Authors

Kelly et al.

Correct Study? (check the reference number, the first number of the filename, against the reference number in AAP_refs.pdf)

- (selected) Yes
- No

Classification Methods

- [x] MDTs/Experts
- [ ] Diagnostic code/medical records
- [ ] Predetermined Criteria
- [ ] Admission/Confession
- [ ] Conviction/Court Confirmation
- [ ] Witnessed
- [ ] Other:

Comment on Methodology

Describe how the study creates a sample of AHT cases

Method described only in reference #22 (Kelly et al 2020)

Has the study adequately addressed the risk of incorporation bias and/or circular reasoning?

- [ ] Yes
- [x] No

This form was created inside Universidad de La Laguna.

Google Forms

Study Methodology

Reference Number

466

Year

2020

Authors

Pfeiffer et al.

Correct Study? (check the reference number, the first number of the filename, against the reference number in AAP_refs.pdf)

- (x) Yes
- ( ) No

Classification Methods

- [x] MDTs/Experts
- [ ] Diagnostic code/medical records
- [ ] Predetermined Criteria
- [ ] Admission/Confession
- [ ] Conviction/Court Confirmation
- [ ] Witnessed
- [ ] Other:

Comment on Methodology

Describe how the study creates a sample of AHT cases

"AHT was defined as the diagnosis of ICI (confirmed on neuroimaging),
which was due to physical child abuse by parents or caregivers, rather than neglect, according to the decision of a multidisciplinary child protection team at the conclusion of their investigation. This decision was based on the consideration of the relevant social, forensic and clinical features in the context of the presenting history, in accordance with the Australian and New Zealand standard child protection assessment processes."

Has the study adequately addressed the risk of incorporation bias and/or circular reasoning?

- [ ] Yes
- [x] No

This form was created inside Universidad de La Laguna.

Google Forms

Study Methodology

Reference Number

609

Year

1986

Authors

Lambert, Johnson, Hoyt

Correct Study? (check the reference number, the first number of the filename, against the reference number in AAP_refs.pdf)

- (selected) Yes
- ( ) No

Classification Methods

- [ ] MDTs/Experts
- [ ] Diagnostic code/medical records
- [ ] Predetermined Criteria
- [x] Admission/Confession
- [ ] Conviction/Court Confirmation
- [ ] Witnessed
- [ ] Other:

Comment on Methodology

Describe how the study creates a sample of AHT cases

Single case report, babysitter admitted shaking four days prior to admission

Has the study adequately addressed the risk of incorporation bias and/or circular reasoning?

- [ ] Yes
- [x] No

This form was created inside Universidad de La Laguna.

Google Forms

Study Methodology

Reference Number

528

Year

2008

Authors

Oehmichen, Schliess et al

Correct Study? (check the reference number, the first number of the filename, against the reference number in AAP_refs.pdf)

- (•) Yes
- ( ) No

Classification Methods

- [ ] MDTs/Experts
- [ ] Diagnostic code/medical records
- [x] Predetermined Criteria
- [x] Admission/Confession
- [x] Conviction/Court Confirmation
- [ ] Witnessed
- [ ] Other:

Comment on Methodology

Describe how the study creates a sample of AHT cases

18 autopsies on infants reportedly dead from shaking, triad was found in all 18. some of the children did not die for days or weeks after hospitalization

Has the study adequately addressed the risk of incorporation bias and/or circular reasoning?

- [ ] Yes
- [x] No

This form was created inside Universidad de La Laguna.

Google Forms

Study Methodology

Reference Number

521

Year

2009

Authors

Gill Goldfeder et al

Correct Study? (check the reference number, the first number of the filename, against the reference number in AAP_refs.pdf)

- (●) Yes
- ( ) No

Classification Methods

- [x] MDTs/Experts
- [ ] Diagnostic code/medical records
- [ ] Predetermined Criteria
- [x] Admission/Confession
- [ ] Conviction/Court Confirmation
- [ ] Witnessed
- [ ] Other:

Comment on Methodology

Describe how the study creates a sample of AHT cases

10 dead infants with brain pathology without evidence of impact. some caregivers admitted to shaking. others did not.

Has the study adequately addressed the risk of incorporation bias and/or circular reasoning?

- [ ] Yes
- [x] No

This form was created inside Universidad de La Laguna.

Google Forms

Study Methodology

Reference Number

483

Year

2015

Authors

O'Neill Handler et al

Correct Study? (check the reference number, the first number of the filename, against the reference number in AAP_refs.pdf)

- (●) Yes
- ( ) No

Classification Methods

- [x] MDTs/Experts
- [ ] Diagnostic code/medical records
- [ ] Predetermined Criteria
- [ ] Admission/Confession
- [ ] Conviction/Court Confirmation
- [ ] Witnessed
- [ ] Other:

Comment on Methodology

Describe how the study creates a sample of AHT cases

No mention of how AHT was diagnosed in the methods section

Has the study adequately addressed the risk of incorporation bias and/or circular reasoning?

- [ ] Yes
- [x] No

This form was created inside Universidad de La Laguna.

Google Forms

Study Methodology

Reference Number

461

Year

2005

Authors

Datta Stoodley et al

Correct Study? (check the reference number, the first number of the filename, against the reference number in AAP_refs.pdf)

- (•) Yes
- ( ) No

Classification Methods

- [x] MDTs/Experts
- [ ] Diagnostic code/medical records
- [ ] Predetermined Criteria
- [x] Admission/Confession
- [x] Conviction/Court Confirmation
- [ ] Witnessed
- [ ] Other:

Comment on Methodology

Describe how the study creates a sample of AHT cases

All findings of NAT taken for granted and used to create expected CT findings with the condition

Has the study adequately addressed the risk of incorporation bias and/or circular reasoning?

- [ ] Yes
- [x] No

This form was created inside Universidad de La Laguna.

Google Forms

Study Methodology

Reference Number

175

Year

2009

Authors

Binenbaum, Mirza George et al

Correct Study? (check the reference number, the first number of the filename, against the reference number in AAP_refs.pdf)

- (selected) Yes
- ( ) No

Classification Methods

- [ ] MDTs/Experts
- [ ] Diagnostic code/medical records
- [x] Predetermined Criteria
- [ ] Admission/Confession
- [ ] Conviction/Court Confirmation
- [ ] Witnessed
- [ ] Other:

Comment on Methodology

Describe how the study creates a sample of AHT cases

it was a retrospective study, and so the decision had already been made about the diagnosis of abuse using all available information. to then 'remove' the eye findings and decide on the abuse diagnosis is irrelevant

Has the study adequately addressed the risk of incorporation bias and/or circular reasoning?

- [ ] Yes
- [x] No

This form was created inside Universidad de La Laguna.

Google Forms

Study Methodology

Reference Number

147

Year

2014

Authors

Breazzano Hudson et al

Correct Study? (check the reference number, the first number of the filename, against the reference number in AAP_refs.pdf)

- (•) Yes
- ( ) No

Classification Methods

- [ ] MDTs/Experts
- [ ] Diagnostic code/medical records
- [ ] Predetermined Criteria
- [x] Admission/Confession
- [x] Conviction/Court Confirmation
- [ ] Witnessed
- [ ] Other: ____

Comment on Methodology

Describe how the study creates a sample of AHT cases

Pathology of eyes from cases diagnosed by confession or conviction

Has the study adequately addressed the risk of incorporation bias and/or circular reasoning?

- [ ] Yes
- [x] No

This form was created inside Universidad de La Laguna.

Google Forms

Study Methodology

Reference Number

116

Year

2018

Authors

Orru Huisman Izbudak

Correct Study? (check the reference number, the first number of the filename, against the reference number in AAP_refs.pdf)

- (•) Yes
- ( ) No

Classification Methods

- [x] MDTs/Experts
- [ ] Diagnostic code/medical records
- [ ] Predetermined Criteria
- [ ] Admission/Confession
- [ ] Conviction/Court Confirmation
- [ ] Witnessed
- [ ] Other: ____

Comment on Methodology

Describe how the study creates a sample of AHT cases

Review of imaging looking for HIE in those diagnosed as AHT by the MDT

Has the study adequately addressed the risk of incorporation bias and/or circular reasoning?

- [ ] Yes
- [x] No

This form was created inside Universidad de La Laguna.

Google Forms

Study Methodology

Reference Number

15

Year

2008

Authors

Kelly Farrant

Correct Study? (check the reference number, the first number of the filename, against the reference number in AAP_refs.pdf)

- (•) Yes
- ( ) No

Classification Methods

- [x] MDTs/Experts
- [x] Diagnostic code/medical records
- [x] Predetermined Criteria
- [ ] Admission/Confession
- [ ] Conviction/Court Confirmation
- [ ] Witnessed
- [ ] Other:

Comment on Methodology

Describe how the study creates a sample of AHT cases

A mix of prospective and retrospective cases. Basis of diagnosis was infantile subdural hematoma. Described accidents were classified as credible or not.

Has the study adequately addressed the risk of incorporation bias and/or circular reasoning?

- [ ] Yes
- [x] No

This form was created inside Universidad de La Laguna.

Google Forms

Study Methodology

Reference Number

62

Year

2013

Authors

Sheets et al.

Correct Study? (check the reference number, the first number of the filename, against the reference number in AAP_refs.pdf)

- (●) Yes
- ( ) No

Classification Methods

- [x] MDTs/Experts
- [x] Diagnostic code/medical records
- [ ] Predetermined Criteria
- [ ] Admission/Confession
- [ ] Conviction/Court Confirmation
- [ ] Witnessed
- [ ] Other:

Comment on Methodology

Describe how the study creates a sample of AHT cases

Sheets et al. explores the relationship between sentinel injuries (primarily bruising) and later diagnosis of abusive head trauma (AHT). They claim to have addressed circular reasoning by retrospectively looking at their classifications to see whether sentinel injuries played a role. The authors claim that sentinel injuries did not influence case classification in any way.

However, during the classification process, the CPT and authors had full access to the medical records, including sentinel injuries, while assigning abuse status. In a study explicitly investigating the link between sentinel injuries and abuse, this setup invites confirmation bias. This looks a textbook case of circular reasoning: the exposure (sentinel injury) informs the diagnosis, which is then used to assess whether the exposure predicts the diagnosis.

To address circular reasoning, the assessors should have been blinded to sentinel injuries. Without blinded, independent classification, their claim that bruising did not affect decisions is unverifiable and subjective.

Indeed, the finding of zero bruises in the control group, implying 100% specificity, is highly implausible. Prior studies (e.g., Kemp et al.) have shown bruising in 6–7% of non-abused infants. 100% sepecificity strongly suggests selection bias and circularity: if bruising prevented a case from being placed in the control group, then the absence of bruising in that group is a foregone conclusion, not a meaningful finding.

The study also suffers from a more subtle form of incorporation bias: bruising may reflect underlying medical vulnerability (e.g., genetic condition, coagulopathy, or general fragility), which also contributes to the later findings associated with AHT. In such cases, both the sentinel injury and the diagnosis may stem from the same underlying cause—not from abuse. This confounding relationship is neither explored nor controlled for, and it further limits the validity of any causal claim between sentinel injuries and abuse diagnosis. There remains no concrete evidence of abuse that rules out this alternative explanation.

Despite claiming to avoid circular reasoning, the study's design allows the presence of sentinel injuries to shape cohort classification, without blinding. Combined with clear signs of incorporation bias and implausible results (e.g., 100% specificity), the conclusions are not methodologically sound.

At most, the study shows a correlation between sentinel injuries and other findings associated with abuse, but it cannot demonstrate causation.

Has the study adequately addressed the risk of incorporation bias and/or circular reasoning?

- [ ] Yes
- [x] No

This form was created inside Universidad de La Laguna.

Google Forms

Study Methodology

Reference Number

523

Year

2017

Authors

Serinelli et al.

Correct Study? (check the reference number, the first number of the filename, against the reference number in AAP_refs.pdf)

- (x) Yes
- ( ) No

Classification Methods

- [ ] MDTs/Experts
- [x] Diagnostic code/medical records
- [ ] Predetermined Criteria
- [ ] Admission/Confession
- [ ] Conviction/Court Confirmation
- [ ] Witnessed
- [ ] Other:

Comment on Methodology

Describe how the study creates a sample of AHT cases

"the files of the Cook County Medical Examiner's Office in Chicago, Illinois, between 2007 and 2012 to identify deaths of children under 3 years old whose cause and manner of death was determined to be homicide due to child abuse."

Has the study adequately addressed the risk of incorporation bias and/or circular reasoning?

- [ ] Yes
- [x] No

This form was created inside Universidad de La Laguna.

Google Forms

Study Methodology

Reference Number

167

Year

2005

Authors

Spitzer et al.

Correct Study? (check the reference number, the first number of the filename, against the reference number in AAP_refs.pdf)

- (●) Yes
- ( ) No

Classification Methods

- [x] MDTs/Experts
- [ ] Diagnostic code/medical records
- [ ] Predetermined Criteria
- [ ] Admission/Confession
- [ ] Conviction/Court Confirmation
- [ ] Witnessed
- [ ] Other:

Comment on Methodology

Describe how the study creates a sample of AHT cases

In each case they describe findings and why those findings lead to the diagnosis... no confessions or witnessed are mentioned in any case.. they simply make a diagnosis based on the medical findings

Has the study adequately addressed the risk of incorporation bias and/or circular reasoning?

- [ ] Yes
- [x] No

This form was created inside Universidad de La Laguna.

Google Forms

Study Methodology

Reference Number

356

Year

2003

Authors

Wells and Sty

Correct Study? (check the reference number, the first number of the filename, against the reference number in AAP_refs.pdf)

- (selected) Yes
- ( ) No

Classification Methods

- [x] MDTs/Experts
- [ ] Diagnostic code/medical records
- [ ] Predetermined Criteria
- [ ] Admission/Confession
- [ ] Conviction/Court Confirmation
- [ ] Witnessed
- [ ] Other: ____

Comment on Methodology

Describe how the study creates a sample of AHT cases

The authors (as "experts") "determined the most likely.. mechanism of injury from the medical records"

Has the study adequately addressed the risk of incorporation bias and/or circular reasoning?

- [ ] Yes
- [x] No

This form was created inside Universidad de La Laguna.

Google Forms

Study Methodology

Reference Number

526

Year

2015

Authors

Matschke et al.

Correct Study? (check the reference number, the first number of the filename, against the reference number in AAP_refs.pdf)

- (•) Yes
- ( ) No

Classification Methods

- [x] MDTs/Experts
- [ ] Diagnostic code/medical records
- [ ] Predetermined Criteria
- [x] Admission/Confession
- [x] Conviction/Court Confirmation
- [ ] Witnessed
- [ ] Other:

Comment on Methodology

Describe how the study creates a sample of AHT cases

A/ confession, or B/conviction, or C/ case conference with at least 3 of: serious external injury (hematomas or lacerations), unexplained fractures (long bones, ribs, or skull), SDH, traumatic intracerebral pathologic conditions (simple contusions or gliding contusions), retinal hemorrhage, and/or an alleged history overtly inadequate to the clinical picture.

Has the study adequately addressed the risk of incorporation bias and/or circular reasoning?

- [ ] Yes
- [x] No

This form was created inside Universidad de La Laguna.

Google Forms

Study Methodology

Reference Number

57

Year

2006

Authors

Graupman and Winston

Correct Study? (check the reference number, the first number of the filename, against the reference number in AAP_refs.pdf)

- (●) Yes
- ( ) No

Classification Methods

- [x] MDTs/Experts
- [ ] Diagnostic code/medical records
- [ ] Predetermined Criteria
- [ ] Admission/Confession
- [ ] Conviction/Court Confirmation
- [ ] Witnessed
- [ ] Other:

Comment on Methodology

Describe how the study creates a sample of AHT cases

Abusive head trauma was defined as radiographic evidence of intracranial injury and documentation from a multidisciplinary child protection team that the injury was nonaccidental.
The paper is bizarre: Results:"The most common abnormality on imaging studies was subdural hematoma (89%);" Conclusion "Evidence for hypoxic brain injury was our most common finding on CT scans." "Significant extraaxial hematomas were demonstrated in a few patients; however, the majority had small subdural hematomas only".

Has the study adequately addressed the risk of incorporation bias and/or circular reasoning?

- [ ] Yes
- [x] No

This form was created inside Universidad de La Laguna.

Google Forms

Study Methodology

Reference Number

211

Year

2003

Authors

King et al.

Correct Study? (check the reference number, the first number of the filename, against the reference number in AAP_refs.pdf)

- (●) Yes
- ( ) No

Classification Methods

- [x] MDTs/Experts
- [x] Diagnostic code/medical records
- [ ] Predetermined Criteria
- [ ] Admission/Confession
- [ ] Conviction/Court Confirmation
- [ ] Witnessed
- [ ] Other:

Comment on Methodology

Describe how the study creates a sample of AHT cases

Shaken baby syndrome was defined as any case reported at each institution of intracranial, intraocular or cervical spine injury resulting from a substantiated or suspected shaking, with or without impact, in children aged less than 5 years. Diagnosed by CAP and using ICD codes

Has the study adequately addressed the risk of incorporation bias and/or circular reasoning?

- [ ] Yes
- [x] No

This form was created inside Universidad de La Laguna.

Google Forms

Study Methodology

Reference Number

143

Year

2000

Authors

Kivlin et al.

Correct Study? (check the reference number, the first number of the filename, against the reference number in AAP_refs.pdf)

- (•) Yes
- ( ) No

Classification Methods

- [x] MDTs/Experts
- [ ] Diagnostic code/medical records
- [x] Predetermined Criteria
- [ ] Admission/Confession
- [ ] Conviction/Court Confirmation
- [ ] Witnessed
- [ ] Other:

Comment on Methodology

Describe how the study creates a sample of AHT cases

Children under 3 years with subdural hematomas of the brain secondary to abuse. Abuse determined by CAPs

Has the study adequately addressed the risk of incorporation bias and/or circular reasoning?

- [ ] Yes
- [x] No

This form was created inside Universidad de La Laguna.

Google Forms

Study Methodology

Reference Number

96

Year

1992

Authors

Duhaime et al.

Correct Study? (check the reference number, the first number of the filename, against the reference number in AAP_refs.pdf)

- (●) Yes
- ( ) No

Classification Methods

- [x] MDTs/Experts
- [ ] Diagnostic code/medical records
- [x] Predetermined Criteria
- [ ] Admission/Confession
- [ ] Conviction/Court Confirmation
- [ ] Witnessed
- [ ] Other:

Comment on Methodology

Describe how the study creates a sample of AHT cases

Children under two with head trauma. Fall heights were analysed. Falls under 3 feet were determined as minor and incapable of causing head trauma, therefore AHT assumed

Has the study adequately addressed the risk of incorporation bias and/or circular reasoning?

- [ ] Yes
- [x] No

This form was created inside Universidad de La Laguna.

Google Forms

Study Methodology

Reference Number

191

Year

2002

Authors

Schloff et al.

Correct Study? (check the reference number, the first number of the filename, against the reference number in AAP_refs.pdf)

- (●) Yes
- ( ) No

Classification Methods

- [x] MDTs/Experts
- [ ] Diagnostic code/medical records
- [ ] Predetermined Criteria
- [ ] Admission/Confession
- [ ] Conviction/Court Confirmation
- [ ] Witnessed
- [ ] Other:

Comment on Methodology

Describe how the study creates a sample of AHT cases

Children with intracranial haemorrhage from non-abuse. Children with abuse excluded on basis of CAP determination but criteria are not described. Old children, 5 m to 16 years, mean age 10 years

Has the study adequately addressed the risk of incorporation bias and/or circular reasoning?

- [ ] Yes
- [x] No

This form was created inside Universidad de La Laguna.

Google Forms

Study Methodology

Reference Number

393

Year

1996

Authors

Shugerman et al.

Correct Study? (check the reference number, the first number of the filename, against the reference number in AAP_refs.pdf)

- (selected) Yes
- ( ) No

Classification Methods

- [x] MDTs/Experts
- [ ] Diagnostic code/medical records
- [ ] Predetermined Criteria
- [ ] Admission/Confession
- [ ] Conviction/Court Confirmation
- [ ] Witnessed
- [ ] Other:

Comment on Methodology

Describe how the study creates a sample of AHT cases

Babies with SDH and EDH. Abuse determined by CAPs but no criteria are given

Has the study adequately addressed the risk of incorporation bias and/or circular reasoning?

- [ ] Yes
- [x] No

This form was created inside Universidad de La Laguna.

Google Forms

Study Methodology

Reference Number

378

Year

1987

Authors

Sinal and Ball

Correct Study? (check the reference number, the first number of the filename, against the reference number in AAP_refs.pdf)

- (•) Yes
- ( ) No

Classification Methods

- [ ] MDTs/Experts
- [x] Diagnostic code/medical records
- [x] Predetermined Criteria
- [x] Admission/Confession
- [x] Conviction/Court Confirmation
- [ ] Witnessed
- [ ] Other:

Comment on Methodology

Describe how the study creates a sample of AHT cases

Children <5 years with head injury and with intracranial or intraocular haemorrhages and no external signs of trauma to head.

Has the study adequately addressed the risk of incorporation bias and/or circular reasoning?

- [ ] Yes
- [x] No

This form was created inside Universidad de La Laguna.

Google Forms

Study Methodology

Reference Number

141

Year

2008

Authors

Sturm et al.

Correct Study? (check the reference number, the first number of the filename, against the reference number in AAP_refs.pdf)

- (●) Yes
- ( ) No

Classification Methods

- [ ] MDTs/Experts
- [ ] Diagnostic code/medical records
- [ ] Predetermined Criteria
- [ ] Admission/Confession
- [ ] Conviction/Court Confirmation
- [ ] Witnessed
- [ ] Other:

Comment on Methodology

Describe how the study creates a sample of AHT cases

3 infants, 4-7 months with suspected SBS. No diagnostic criteria stated. None, so do not know how they were classified

Has the study adequately addressed the risk of incorporation bias and/or circular reasoning?

- [ ] Yes
- [x] No

This form was created inside Universidad de La Laguna.

Google Forms

Study Methodology

Reference Number

221

Year

2014

Authors

SieswerdaHoogendoorn et al.

Correct Study? (check the reference number, the first number of the filename, against the reference number in AAP_refs.pdf)

- (●) Yes
- ( ) No

Classification Methods

- [x] MDTs/Experts
- [ ] Diagnostic code/medical records
- [ ] Predetermined Criteria
- [ ] Admission/Confession
- [ ] Conviction/Court Confirmation
- [ ] Witnessed
- [ ] Other:

Comment on Methodology

Describe how the study creates a sample of AHT cases

Retrospective assessment of cases under 5 diagnosed by forensic physician. No criteria given.

Has the study adequately addressed the risk of incorporation bias and/or circular reasoning?

- [ ] Yes
- [x] No

This form was created inside Universidad de La Laguna.

Google Forms

Study Methodology

Reference Number

392

Year

2022

Authors

O'Hara et al.

Correct Study? (check the reference number, the first number of the filename, against the reference number in AAP_refs.pdf)

- (●) Yes
- ( ) No

Classification Methods

- [x] MDTs/Experts
- [ ] Diagnostic code/medical records
- [ ] Predetermined Criteria
- [ ] Admission/Confession
- [ ] Conviction/Court Confirmation
- [ ] Witnessed
- [ ] Other:

Comment on Methodology

Describe how the study creates a sample of AHT cases

The child abuse consultation team´s "final determination of cause of injury at the time of initial consultation was recorded as definite accidental trauma, definite abusive injury, or indeterminate if a definite determination was not made."

Has the study adequately addressed the risk of incorporation bias and/or circular reasoning?

- [ ] Yes
- [x] No

This form was created inside Universidad de La Laguna.

Google Forms

Study Methodology

Reference Number

45

Year

2016

Authors

Berger et al.

Correct Study? (check the reference number, the first number of the filename, against the reference number in AAP_refs.pdf)

- (●) Yes
- ( ) No

Classification Methods

- [x] MDTs/Experts
- [ ] Diagnostic code/medical records
- [ ] Predetermined Criteria
- [ ] Admission/Confession
- [ ] Conviction/Court Confirmation
- [ ] Witnessed
- [ ] Other:

Comment on Methodology

Describe how the study creates a sample of AHT cases

"AHT diagnosis was defined as a brain injury that was assessed by each site's hospital-based Child Protection Team (CPT) as being due to definite or probable, but not possible, abuse."

Has the study adequately addressed the risk of incorporation bias and/or circular reasoning?

- [ ] Yes
- [x] No

This form was created inside Universidad de La Laguna.

Google Forms

Study Methodology

Reference Number

53

Year

2015

Authors

Feldman et al.

Correct Study? (check the reference number, the first number of the filename, against the reference number in AAP_refs.pdf)

- (●) Yes
- ( ) No

Classification Methods

- [x] MDTs/Experts
- [ ] Diagnostic code/medical records
- [ ] Predetermined Criteria
- [ ] Admission/Confession
- [ ] Conviction/Court Confirmation
- [ ] Witnessed
- [ ] Other:

Comment on Methodology

Describe how the study creates a sample of AHT cases

"Judgment that AHT was unequivocal was made by consensus of the CPTs of the contributing hospitals. The means by which this decision was made by the individual child's
caretaking team and CPTs for the parent study was not recorded "

Has the study adequately addressed the risk of incorporation bias and/or circular reasoning?

- [ ] Yes
- [x] No

This form was created inside Universidad de La Laguna.

Google Forms

Study Methodology

Reference Number

480

Year

2011

Authors

Liesemer et al.

Correct Study? (check the reference number, the first number of the filename, against the reference number in AAP_refs.pdf)

- (•) Yes
- ( ) No

Classification Methods

- [x] MDTs/Experts
- [ ] Diagnostic code/medical records
- [ ] Predetermined Criteria
- [ ] Admission/Confession
- [ ] Conviction/Court Confirmation
- [ ] Witnessed
- [ ] Other:

Comment on Methodology

Describe how the study creates a sample of AHT cases

"the determination of NAT was made by the hospital’s child abuse team."

Has the study adequately addressed the risk of incorporation bias and/or circular reasoning?

- [ ] Yes
- [x] No

This form was created inside Universidad de La Laguna.

Google Forms

Study Methodology

Reference Number

169

Year

1992

Authors

Buys et al.

Correct Study? (check the reference number, the first number of the filename, against the reference number in AAP_refs.pdf)

- (selected) Yes
- No

Classification Methods

- [x] MDTs/Experts
- [ ] Diagnostic code/medical records
- [ ] Predetermined Criteria
- [ ] Admission/Confession
- [ ] Conviction/Court Confirmation
- [ ] Witnessed
- [ ] Other:

Comment on Methodology

Describe how the study creates a sample of AHT cases

"categorization was based on the results of standard multidisciplinary evaluations (i.e., child abuse team, social work, law enforcement) obtained in cases suspicious for child abuse on the basis of history and or clinical findings. Children with retinal hemorrhages were automatically referred for investigation. if child abuse was not suspected, no referral was made, and the child was assigned to the accidental group"

Has the study adequately addressed the risk of incorporation bias and/or circular reasoning?

- [ ] Yes
- [x] No

This form was created inside Universidad de La Laguna.

Google Forms

Study Methodology

Reference Number

163

Year

2013

Authors

Greiner et al.

Correct Study? (check the reference number, the first number of the filename, against the reference number in AAP_refs.pdf)

- (•) Yes
- ( ) No

Classification Methods

- [x] MDTs/Experts
- [ ] Diagnostic code/medical records
- [ ] Predetermined Criteria
- [ ] Admission/Confession
- [ ] Conviction/Court Confirmation
- [ ] Witnessed
- [ ] Other:

Comment on Methodology

Describe how the study creates a sample of AHT cases

CAPs "recorded their perceived likelihood of abuse based on a previously published 7-point scale; a score of 6 or 7 was considered ‘high likelihood.’ "

Has the study adequately addressed the risk of incorporation bias and/or circular reasoning?

- [ ] Yes
- [x] No

This form was created inside Universidad de La Laguna.

Google Forms

Study Methodology

Reference Number

524

Year

1993

Authors

Munger et al.

Correct Study? (check the reference number, the first number of the filename, against the reference number in AAP_refs.pdf)

- (●) Yes
- ( ) No

Classification Methods

- [x] MDTs/Experts
- [ ] Diagnostic code/medical records
- [ ] Predetermined Criteria
- [ ] Admission/Confession
- [ ] Conviction/Court Confirmation
- [ ] Witnessed
- [ ] Other:

Comment on Methodology

Describe how the study creates a sample of AHT cases

AHT/SBS cases selected via "the clinical and pathologic diagnosis of established or suspected shaken baby syndrome"

Has the study adequately addressed the risk of incorporation bias and/or circular reasoning?

- [ ] Yes
- [x] No

This form was created inside Universidad de La Laguna.

Google Forms

Study Methodology

Reference Number

115

Year

2001

Authors

Suh et al.

Correct Study? (check the reference number, the first number of the filename, against the reference number in AAP_refs.pdf)

- (selected) Yes
- No

Classification Methods

- [x] MDTs/Experts
- [ ] Diagnostic code/medical records
- [x] Predetermined Criteria
- [ ] Admission/Confession
- [ ] Conviction/Court Confirmation
- [ ] Witnessed
- [ ] Other:

Comment on Methodology

Describe how the study creates a sample of AHT cases

"the determination of nonaccidental versus accidental head injury was based on an algorithm initially proposed by Duhaime et al. (13) and used by others in a modified form" and "A multidisciplinary team of clinicians, including a pediatric social services consultant and a pediatrician trained in child abuse investigation, examined all patients"

Has the study adequately addressed the risk of incorporation bias and/or circular reasoning?

- [ ] Yes
- [x] No

This form was created inside Universidad de La Laguna.

Google Forms

Study Methodology

Reference Number

522

Year

1996

Authors

Gilliland and Folberg

Correct Study? (check the reference number, the first number of the filename, against the reference number in AAP_refs.pdf)

- (selected) Yes
- ( ) No

Classification Methods

- [ ] MDTs/Experts
- [ ] Diagnostic code/medical records
- [x] Predetermined Criteria
- [ ] Admission/Confession
- [ ] Conviction/Court Confirmation
- [ ] Witnessed
- [ ] Other:

Comment on Methodology

Describe how the study creates a sample of AHT cases

Deaths were classified as shaking mechanism by the presence of two or more of the following criteria: 1) finger marks and/or rib fractures, 2) subdural and/or subarachnoid hemorrhage, or 3) a history of vigorous shaking.

Has the study adequately addressed the risk of incorporation bias and/or circular reasoning?

- [ ] Yes
- [x] No

This form was created inside Universidad de La Laguna.

Google Forms

Study Methodology

Reference Number

100

Year

1998

Authors

EwingCobbs et al.

Correct Study? (check the reference number, the first number of the filename, against the reference number in AAP_refs.pdf)

- ○ Yes
- ○ No

Classification Methods

- [x] MDTs/Experts
- [ ] Diagnostic code/medical records
- [x] Predetermined Criteria
- [ ] Admission/Confession
- [ ] Conviction/Court Confirmation
- [ ] Witnessed
- [ ] Other:

Comment on Methodology

Describe how the study creates a sample of AHT cases

"determination of whether an injury was inflicted or noninflicted was based on the assessment of the Child Protection Committee at each hospital and Harris County Children's Protective Services...Similar to the algorithm devised by Duhaime et al4 to detect probable inflicted injury, injuries incompatible with the stated mechanism of injury (eg, bilateral subdural hematomas and retinal hemorrhages with a history of falling from a couch; multiple skull fractures, multiple intracranial hemorrhages, and bilateral cephalohematomas with a history of falling 4 feet) and unexplained injuries (eg, no history of trauma in conjunction with intracranial injuries and old skeletal fractures) were presumed to indicate assault. Additional variables associated with inflicted injury such as delay in seeking treatment and inconsistent or changing history were also considered "

Has the study adequately addressed the risk of incorporation bias and/or circular reasoning?

- [ ] Yes
- [x] No

This form was created inside Universidad de La Laguna.

Google Forms

Study Methodology

Reference Number

172

Year

2013

Authors

Pérez et al.

Correct Study? (check the reference number, the first number of the filename, against the reference number in AAP_refs.pdf)

- (●) Yes
- ( ) No

Classification Methods

- [ ] MDTs/Experts
- [x] Diagnostic code/medical records
- [ ] Predetermined Criteria
- [ ] Admission/Confession
- [ ] Conviction/Court Confirmation
- [ ] Witnessed
- [ ] Other:

Comment on Methodology

Describe how the study creates a sample of AHT cases

Single case report. Acute and chronic SDH and RH. No other criteria stated

Has the study adequately addressed the risk of incorporation bias and/or circular reasoning?

- [ ] Yes
- [x] No

This form was created inside Universidad de La Laguna.

Google Forms

Study Methodology

Reference Number

105

Year

1997

Authors

Hymel et al.

Correct Study? (check the reference number, the first number of the filename, against the reference number in AAP_refs.pdf)

- (●) Yes
- ( ) No

Classification Methods

- [x] MDTs/Experts
- [ ] Diagnostic code/medical records
- [ ] Predetermined Criteria
- [ ] Admission/Confession
- [ ] Conviction/Court Confirmation
- [ ] Witnessed
- [ ] Other:

Comment on Methodology

Describe how the study creates a sample of AHT cases

AHT "determined by an experienced multidisciplinary child abuse team "

Has the study adequately addressed the risk of incorporation bias and/or circular reasoning?

- [ ] Yes
- [x] No

This form was created inside Universidad de La Laguna.

Google Forms

Study Methodology

Reference Number

517

Year

2001

Authors

Geddes

Correct Study? (check the reference number, the first number of the filename, against the reference number in AAP_refs.pdf)

- (●) Yes
- ( ) No

Classification Methods

- [ ] MDTs/Experts
- [ ] Diagnostic code/medical records
- [x] Predetermined Criteria
- [x] Admission/Confession
- [x] Conviction/Court Confirmation
- [ ] Witnessed
- [ ] Other:

Comment on Methodology

Describe how the study creates a sample of AHT cases

" The diagnostic criteria used were: (i) head injuries in which there had been a confession by the perpetrator (n = 7); (ii) cases in which non-accidental head injury had been established as a result of conviction in a criminal court, and in which there were also unexplained extracranial injuries to support this (n = 19); (iii) cases with unexplained injuries elsewhere in the body, in addition to the head injury, but no conviction (n = 8); (iv) cases in which the carer was tried and convicted of injuring the child, but in which there were no extracranial injuries (n = 12); and (v) cases in which there was a major discrepancy between the explanation of the incident given by the carer and significant injuries such as a skull fracture, or if the history was developmentally incompatible (n = 7).

Has the study adequately addressed the risk of incorporation bias and/or circular reasoning?

- [ ] Yes
- [x] No

This form was created inside Universidad de La Laguna.

Google Forms

Study Methodology

Reference Number

83

Year

2014

Authors

Lopez et al.

Correct Study? (check the reference number, the first number of the filename, against the reference number in AAP_refs.pdf)

- (•) Yes
- ( ) No

Classification Methods

- [x] MDTs/Experts
- [ ] Diagnostic code/medical records
- [ ] Predetermined Criteria
- [ ] Admission/Confession
- [ ] Conviction/Court Confirmation
- [ ] Witnessed
- [ ] Other:

Comment on Methodology

Describe how the study creates a sample of AHT cases

Intubated infants less than 3 years with MDT determination of abuse. No criteria

Has the study adequately addressed the risk of incorporation bias and/or circular reasoning?

- [ ] Yes
- [x] No

This form was created inside Universidad de La Laguna.

Google Forms

Study Methodology

Reference Number

91

Year

1998

Authors

Dias et al.

Correct Study? (check the reference number, the first number of the filename, against the reference number in AAP_refs.pdf)

- (●) Yes
- ( ) No

Classification Methods

- [ ] MDTs/Experts
- [x] Diagnostic code/medical records
- [ ] Predetermined Criteria
- [ ] Admission/Confession
- [ ] Conviction/Court Confirmation
- [ ] Witnessed
- [ ] Other:

Comment on Methodology

Describe how the study creates a sample of AHT cases

No criteria stated. Assumed AHT from hospital records

Has the study adequately addressed the risk of incorporation bias and/or circular reasoning?

- [ ] Yes
- [x] No

This form was created inside Universidad de La Laguna.

Google Forms

Study Methodology

Reference Number

93

Year

2004

Authors

Bechtel et al.

Correct Study? (check the reference number, the first number of the filename, against the reference number in AAP_refs.pdf)

- (x) Yes
- ( ) No

Classification Methods

- [x] MDTs/Experts
- [ ] Diagnostic code/medical records
- [x] Predetermined Criteria
- [x] Admission/Confession
- [ ] Conviction/Court Confirmation
- [ ] Witnessed
- [ ] Other:

Comment on Methodology

Describe how the study creates a sample of AHT cases

Infants under 2 y with head injury.

Has the study adequately addressed the risk of incorporation bias and/or circular reasoning?

- [ ] Yes
- [x] No

This form was created inside Universidad de La Laguna.

Google Forms

Study Methodology

Reference Number

374

Year

2016

Authors

Binenbaum et al.

Correct Study? (check the reference number, the first number of the filename, against the reference number in AAP_refs.pdf)

- (●) Yes
- ( ) No

Classification Methods

- [x] MDTs/Experts
- [ ] Diagnostic code/medical records
- [ ] Predetermined Criteria
- [ ] Admission/Confession
- [ ] Conviction/Court Confirmation
- [ ] Witnessed
- [ ] Other:

Comment on Methodology

Describe how the study creates a sample of AHT cases

Children <2y with RH. No criteria for abuse stated

Has the study adequately addressed the risk of incorporation bias and/or circular reasoning?

- [ ] Yes
- [x] No

This form was created inside Universidad de La Laguna.

Google Forms

Study Methodology

Reference Number

162

Year

2020

Authors

Ip et al.

Correct Study? (check the reference number, the first number of the filename, against the reference number in AAP_refs.pdf)

- (selected) Yes
- ( ) No

Classification Methods

- [ ] MDTs/Experts
- [x] Diagnostic code/medical records
- [ ] Predetermined Criteria
- [ ] Admission/Confession
- [ ] Conviction/Court Confirmation
- [ ] Witnessed
- [ ] Other:

Comment on Methodology

Describe how the study creates a sample of AHT cases

injuries in children less than 1 year of age that are not consistent with history, injuries as a result of family violence, or history of brief resolved un-explained event (also known as BRUE).

Has the study adequately addressed the risk of incorporation bias and/or circular reasoning?

- [ ] Yes
- [x] No

This form was created inside Universidad de La Laguna.

Google Forms

Study Methodology

Reference Number

421

Year

2018

Authors

Kim et al.

Correct Study? (check the reference number, the first number of the filename, against the reference number in AAP_refs.pdf)

- (●) Yes
- ( ) No

Classification Methods

- [x] MDTs/Experts
- [x] Diagnostic code/medical records
- [ ] Predetermined Criteria
- [ ] Admission/Confession
- [ ] Conviction/Court Confirmation
- [ ] Witnessed
- [ ] Other:

Comment on Methodology

Describe how the study creates a sample of AHT cases

AHT was defined as an injury to the skull or the intracranial contents of an infant or young child caused by inflicted blunt impact or blunt impact and violent shaking.

Has the study adequately addressed the risk of incorporation bias and/or circular reasoning?

- [ ] Yes
- [x] No

This form was created inside Universidad de La Laguna.

Google Forms

Study Methodology

Reference Number

531

Year

2011

Authors

Matshes et al.

Correct Study? (check the reference number, the first number of the filename, against the reference number in AAP_refs.pdf)

- (●) Yes
- ( ) No

Classification Methods

- [ ] MDTs/Experts
- [x] Diagnostic code/medical records
- [ ] Predetermined Criteria
- [ ] Admission/Confession
- [ ] Conviction/Court Confirmation
- [ ] Witnessed
- [ ] Other:

Comment on Methodology

Describe how the study creates a sample of AHT cases

Autopsy study. Abused babies defined as "confirmed or suspected by history and circumstances to have been subjected to hyperextension and hyperflexion forces, either as a result of accident or homicide".

Has the study adequately addressed the risk of incorporation bias and/or circular reasoning?

- [ ] Yes
- [x] No

This form was created inside Universidad de La Laguna.

Google Forms

Study Methodology

Reference Number

193

Year

2012

Authors

Agrawal et al.

Correct Study? (check the reference number, the first number of the filename, against the reference number in AAP_refs.pdf)

- (●) Yes
- ( ) No

Classification Methods

- [x] MDTs/Experts
- [ ] Diagnostic code/medical records
- [ ] Predetermined Criteria
- [ ] Admission/Confession
- [ ] Conviction/Court Confirmation
- [ ] Witnessed
- [ ] Other:

Comment on Methodology

Describe how the study creates a sample of AHT cases

"a multiprofessional team ... examined all the evidence" to exclude cases of AHT. Presumably they were, at least in part, using RH ias part of "all evidence", which means cases with RH are excluded from their analysis of RH in critically ill children.

Has the study adequately addressed the risk of incorporation bias and/or circular reasoning?

- [ ] Yes
- [x] No

This form was created inside Universidad de La Laguna.

Google Forms

Study Methodology

Reference Number

388

Year

2013

Authors

Hasbani et al.

Correct Study? (check the reference number, the first number of the filename, against the reference number in AAP_refs.pdf)

- (•) Yes
- ( ) No

Classification Methods

- [x] MDTs/Experts
- [ ] Diagnostic code/medical records
- [ ] Predetermined Criteria
- [ ] Admission/Confession
- [ ] Conviction/Court Confirmation
- [ ] Witnessed
- [ ] Other:

Comment on Methodology

Describe how the study creates a sample of AHT cases

"determination of abuse by the Child Protection Team. "

Has the study adequately addressed the risk of incorporation bias and/or circular reasoning?

- [ ] Yes
- [x] No

This form was created inside Universidad de La Laguna.

Google Forms

Study Methodology

Reference Number

542

Year

2015

Authors

Matschke et al.

Correct Study? (check the reference number, the first number of the filename, against the reference number in AAP_refs.pdf)

- (•) Yes
- ( ) No

Classification Methods

- [x] MDTs/Experts
- [ ] Diagnostic code/medical records
- [x] Predetermined Criteria
- [x] Admission/Confession
- [x] Conviction/Court Confirmation
- [ ] Witnessed
- [ ] Other:

Comment on Methodology

Describe how the study creates a sample of AHT cases

AHT victims were identified (i) on the basis of a perpetrator's confession, (ii) conviction for child abuse in a criminal court, or (iii) by case conference as defined perviously. This previous definition was "based on the presence of at least three of the following criteria: signs of serious external injury (hematomas or lacerations), unexplained fractures (of long bones, ribs, or skull), SDH, traumatic intracerebral pathology (e.g., simple or gliding contusions), retinal hemorrhage, and/or a clearly inadequate history given the clinical findings."

Has the study adequately addressed the risk of incorporation bias and/or circular reasoning?

- [ ] Yes
- [x] No

This form was created inside Universidad de La Laguna.

Google Forms

Study Methodology

Reference Number

121

Year

2014

Authors

Choudhary et al.

Correct Study? (check the reference number, the first number of the filename, against the reference number in AAP_refs.pdf)

- (●) Yes
- ( ) No

Classification Methods

- [ ] MDTs/Experts
- [x] Diagnostic code/medical records
- [ ] Predetermined Criteria
- [ ] Admission/Confession
- [ ] Conviction/Court Confirmation
- [ ] Witnessed
- [ ] Other:

Comment on Methodology

Describe how the study creates a sample of AHT cases

"AHT identified from an institutional AHT registry"

Has the study adequately addressed the risk of incorporation bias and/or circular reasoning?

- [ ] Yes
- [x] No

This form was created inside Universidad de La Laguna.

Google Forms

Study Methodology

Reference Number

120

Year

2003

Authors

Kemp et al.

Correct Study? (check the reference number, the first number of the filename, against the reference number in AAP_refs.pdf)

- (●) Yes
- ( ) No

Classification Methods

- [x] MDTs/Experts
- [ ] Diagnostic code/medical records
- [x] Predetermined Criteria
- [x] Admission/Confession
- [x] Conviction/Court Confirmation
- [ ] Witnessed
- [ ] Other:

Comment on Methodology

Describe how the study creates a sample of AHT cases

"(1) Head injury where there was a confession by the perpetrator (n = 19).
(2) Cases where NAHI was established as a result of criminal conviction in the criminal court where there were unexplained extracranial injuries (n = 10).
(3) Cases where there were unexplained injuries elsewhere in the body, other than head injury, but no conviction (all diagnosed at case conference except two who died) (n = 15).
(4) Cases where the carer was convicted of injuring the child but in which there was no extra cranial injury (n = 0).
(5) Cases where there was major discrepancy between the explanation given by the carer and significant injury, such as a skull fracture, or if the history was developmentally incompatible (NAHI diagnosed at case conference in all except one who died) (n = 21)."

Has the study adequately addressed the risk of incorporation bias and/or circular reasoning?

- [ ] Yes
- [x] No

This form was created inside Universidad de La Laguna.

Google Forms

Study Methodology

Reference Number

37

Year

2003

Authors

Hettler and Greenes

Correct Study? (check the reference number, the first number of the filename, against the reference number in AAP_refs.pdf)

- (●) Yes
- ( ) No

Classification Methods

- [ ] MDTs/Experts
- [ ] Diagnostic code/medical records
- [x] Predetermined Criteria
- [x] Admission/Confession
- [ ] Conviction/Court Confirmation
- [x] Witnessed
- [ ] Other:

Comment on Methodology

Describe how the study creates a sample of AHT cases

"Cases were defined as definite abuse when, in addition to intracranial hemorrhage, the patient had 1 or more of the following: witnessed or confessed abuse, retinal hemorrhages consistent with abuse,17–19 high-specificity skin findings (pattern marks),6,20 or moderate- or high-specificity fractures. As per the classification by Nimkin and Kleinman,21 fractures considered to be of moderate or high specificity for abuse included fractures of the sternum,
posterior rib, scapula, spinous process, metaphysis, epiphysis, vertebral body, or digit.21 Fractures of different ages and multiple bilateral fractures were also considered to be of high or moderate specificity for abuse. All other cases were defined as not definite abuse."

Has the study adequately addressed the risk of incorporation bias and/or circular reasoning?

- [ ] Yes
- [x] No

This form was created inside Universidad de La Laguna.

Google Forms

Study Methodology

Reference Number

119

Year

2008

Authors

McKinney et al.

Correct Study? (check the reference number, the first number of the filename, against the reference number in AAP_refs.pdf)

- (selected) Yes
- ( ) No

Classification Methods

- [x] MDTs/Experts
- [ ] Diagnostic code/medical records
- [ ] Predetermined Criteria
- [ ] Admission/Confession
- [x] Conviction/Court Confirmation
- [ ] Witnessed
- [ ] Other:

Comment on Methodology

Describe how the study creates a sample of AHT cases

"Patients were then categorized by the child abuse team’s clinical assessment (in combination with the legal assessment) as to whether they had suffered accidental head
trauma, nonaccidental (abusive) head trauma (AHT), or as-yet-unresolved head trauma (unknown or case pending).

Has the study adequately addressed the risk of incorporation bias and/or circular reasoning?

- [ ] Yes
- [x] No

This form was created inside Universidad de La Laguna.

Google Forms

Study Methodology

Reference Number

149

Year

1988

Authors

Gaynon et al.

Correct Study? (check the reference number, the first number of the filename, against the reference number in AAP_refs.pdf)

- (●) Yes
- ( ) No

Classification Methods

- [x] MDTs/Experts
- [ ] Diagnostic code/medical records
- [ ] Predetermined Criteria
- [ ] Admission/Confession
- [ ] Conviction/Court Confirmation
- [ ] Witnessed
- [ ] Other:

Comment on Methodology

Describe how the study creates a sample of AHT cases

No method mentioned but it seems a medical diagnosis based on clinical findings. One had a history of a fall that was ignored and the other was said to have collapsed in the arms of a babysitter. Not ethat the one with a history of a fall had unileteral hemorrhage. which would corroborate impact, whilst the other had no sign of trauma, which would also corroborate the histpry provided of no trauma.

Has the study adequately addressed the risk of incorporation bias and/or circular reasoning?

- [ ] Yes
- [x] No

This form was created inside Universidad de La Laguna.

Google Forms

Study Methodology

Reference Number

74

Year

2010

Authors

Pierce et al.

Correct Study? (check the reference number, the first number of the filename, against the reference number in AAP_refs.pdf)

- (•) Yes
- ( ) No

Classification Methods

- [x] MDTs/Experts
- [x] Diagnostic code/medical records
- [ ] Predetermined Criteria
- [ ] Admission/Confession
- [ ] Conviction/Court Confirmation
- [ ] Witnessed
- [ ] Other:

Comment on Methodology

Describe how the study creates a sample of AHT cases

The criteria for case subjects (abuse) were as follows: (1) trauma registry categorized the trauma as abuse; (2) hospital medical team determined the injuries to be highly suggestive of abuse; (3) stated cause of injury did not account for the type, severity, and/or number of injuries; (4) history of trauma was absent, vague, or changing; or (5) state social services determined the patient was abused.

Has the study adequately addressed the risk of incorporation bias and/or circular reasoning?

- [ ] Yes
- [x] No

This form was created inside Universidad de La Laguna.

Google Forms

Study Methodology

Reference Number

68

Year

2005

Authors

Hobbs

Correct Study? (check the reference number, the first number of the filename, against the reference number in AAP_refs.pdf)

- ◯ Yes
- ◯ No

Classification Methods

- [x] MDTs/Experts
- [ ] Diagnostic code/medical records
- [ ] Predetermined Criteria
- [ ] Admission/Confession
- [ ] Conviction/Court Confirmation
- [ ] Witnessed
- [ ] Other:

Comment on Methodology

Describe how the study creates a sample of AHT cases

"clinicians' stated aetiology". "This opinion was based on the clinical history, examination, and investigations which in some cases included a child protection investigation. "

Has the study adequately addressed the risk of incorporation bias and/or circular reasoning?

- [ ] Yes
- [x] No

This form was created inside Universidad de La Laguna.

Google Forms

Study Methodology

Reference Number

286

Year

2009

Authors

Brennan et al.

Correct Study? (check the reference number, the first number of the filename, against the reference number in AAP_refs.pdf)

- (●) Yes
- ( ) No

Classification Methods

- [x] MDTs/Experts
- [ ] Diagnostic code/medical records
- [ ] Predetermined Criteria
- [ ] Admission/Confession
- [ ] Conviction/Court Confirmation
- [ ] Witnessed
- [ ] Other:

Comment on Methodology

Describe how the study creates a sample of AHT cases

The mechanism of death was determined and reported by the chief medical examiner based on assessment of the available investigative data and results of the postmortem examination. Based on this report, we separated the cases into 2 categories: abusive head trauma and death consequent to non-CNS injury, including asphyxiation and abdominal trauma.

Has the study adequately addressed the risk of incorporation bias and/or circular reasoning?

- [ ] Yes
- [x] No

This form was created inside Universidad de La Laguna.

Google Forms

Study Methodology

Reference Number

363

Year

2011

Authors

Duffy et al.

Correct Study? (check the reference number, the first number of the filename, against the reference number in AAP_refs.pdf)

- (•) Yes
- ( ) No

Classification Methods

- [x] MDTs/Experts
- [ ] Diagnostic code/medical records
- [ ] Predetermined Criteria
- [ ] Admission/Confession
- [ ] Conviction/Court Confirmation
- [ ] Witnessed
- [ ] Other:

Comment on Methodology

Describe how the study creates a sample of AHT cases

cases were "classified as definite abuse, probable abuse, possible abuse, or not
abuse on the basis of the conclusion of the Children’s Hospital of Pittsburgh child protection team (CPT)."

Has the study adequately addressed the risk of incorporation bias and/or circular reasoning?

- [ ] Yes
- [x] No

This form was created inside Universidad de La Laguna.

Google Forms

Study Methodology

Reference Number

133

Year

1989

Authors

Hadley et al.

Correct Study? (check the reference number, the first number of the filename, against the reference number in AAP_refs.pdf)

- (selected) Yes
- ( ) No

Classification Methods

- [x] MDTs/Experts
- [ ] Diagnostic code/medical records
- [ ] Predetermined Criteria
- [ ] Admission/Confession
- [ ] Conviction/Court Confirmation
- [ ] Witnessed
- [ ] Other:

Comment on Methodology

Describe how the study creates a sample of AHT cases

"The clinical histories, physical examinations, and radio- graphic studies of 36 infants who sustained nonaccidental head injuries were reviewed."

Has the study adequately addressed the risk of incorporation bias and/or circular reasoning?

- [ ] Yes
- [x] No

This form was created inside Universidad de La Laguna.

Google Forms

Study Methodology

Reference Number

160

Year

1991

Authors

Riffenburgh and Sathyavagiswaran

Correct Study? (check the reference number, the first number of the filename, against the reference number in AAP_refs.pdf)

- (•) Yes
- ( ) No

Classification Methods

- [x] MDTs/Experts
- [x] Diagnostic code/medical records
- [ ] Predetermined Criteria
- [ ] Admission/Confession
- [ ] Conviction/Court Confirmation
- [ ] Witnessed
- [ ] Other:

Comment on Methodology

Describe how the study creates a sample of AHT cases

"Based on review ofthe.rec;ords, the cases were divided into three groups. Group 1 (55 cases) included children with a history of abuse or with definite evidence of nonaccidental trauma, such as multiple injuries, sexual assault, human bites, or ligature strangulation. Group 2 (43 cases) included questionable cases of child abuse: those with suspicious, unexplained trauma but inadequate hard evidence of nonaccidental trauma"

Has the study adequately addressed the risk of incorporation bias and/or circular reasoning?

- [ ] Yes
- [x] No

This form was created inside Universidad de La Laguna.

Google Forms

Study Methodology

Reference Number

98

Year

2000

Authors

Reece and Sege

Correct Study? (check the reference number, the first number of the filename, against the reference number in AAP_refs.pdf)

- ○ Yes
- ○ No

Classification Methods

- [ ] MDTs/Experts
- [ ] Diagnostic code/medical records
- [x] Predetermined Criteria
- [x] Admission/Confession
- [ ] Conviction/Court Confirmation
- [x] Witnessed
- [ ] Other: ______

Comment on Methodology

Describe how the study creates a sample of AHT cases

"Definite abuse:
Witnessed abuse
Confession of abuse
No history accounting for patient's serious head injury
Positive skeletal survey (new fractures or fractures of differing ages with no history accounting for the injuries), coupled with serious head injury
Physical findings consistent only with abusive injuries (patterns, old and new lesions, locations), coupled with serious head injury"

No indication of how many cases from each criteria.

Has the study adequately addressed the risk of incorporation bias and/or circular reasoning?

- [ ] Yes
- [x] No

This form was created inside Universidad de La Laguna.

Google Forms

Study Methodology

Reference Number

168

Year

1997

Authors

Kapoor et al.

Correct Study? (check the reference number, the first number of the filename, against the reference number in AAP_refs.pdf)

- (•) Yes
- ( ) No

Classification Methods

- [x] MDTs/Experts
- [ ] Diagnostic code/medical records
- [ ] Predetermined Criteria
- [ ] Admission/Confession
- [ ] Conviction/Court Confirmation
- [ ] Witnessed
- [ ] Other:

Comment on Methodology

Describe how the study creates a sample of AHT cases

"After the emergency physician alerted social services and the pediatric team, a thorough investigation confirmed child abuse. All infectious etiologies and blood disorders were excluded as possible causes in these children."

Has the study adequately addressed the risk of incorporation bias and/or circular reasoning?

- [ ] Yes
- [x] No

This form was created inside Universidad de La Laguna.

Google Forms

Study Methodology

Reference Number

518

Year

1987

Authors

Vowles et al.

Correct Study? (check the reference number, the first number of the filename, against the reference number in AAP_refs.pdf)

- (selected) Yes
- ( ) No

Classification Methods

- [ ] MDTs/Experts
- [x] Diagnostic code/medical records
- [ ] Predetermined Criteria
- [ ] Admission/Confession
- [ ] Conviction/Court Confirmation
- [ ] Witnessed
- [ ] Other:

Comment on Methodology

Describe how the study creates a sample of AHT cases

Simply states that "The brains of nine infants (cases 1-9 from Calderet al, 1984) and one additional case (10), all of which had been subject to non-accidental injury and had died of
head injury, were studied." and in Calder 1984 it says "it was alleged that the infants had been subjected to repeated trauma and had died from trauma to the head."

Has the study adequately addressed the risk of incorporation bias and/or circular reasoning?

- [ ] Yes
- [x] No

This form was created inside Universidad de La Laguna.

Google Forms

Study Methodology

Reference Number

118

Year

2017

Authors

Khan et al.

Correct Study? (check the reference number, the first number of the filename, against the reference number in AAP_refs.pdf)

- (•) Yes
- ( ) No

Classification Methods

- [x] MDTs/Experts
- [ ] Diagnostic code/medical records
- [ ] Predetermined Criteria
- [ ] Admission/Confession
- [ ] Conviction/Court Confirmation
- [ ] Witnessed
- [ ] Other:

Comment on Methodology

Describe how the study creates a sample of AHT cases

"AHT, or nonaccidental trauma (NAT), was defined as skull fracture or intracranial hemorrhage in a child under the age of 5 years with a suspicious mechanism, or direct evidence of other intentional injuries or consequences from such actions (e.g., retinal hemorrhages, old or new fractures, burns, or other soft tissue injury) that was con-
sistent with AHT, according to the final assessment of the CARES [Child Advocacy Resource and Evaluation Services] team."

Has the study adequately addressed the risk of incorporation bias and/or circular reasoning?

- [ ] Yes
- [x] No

This form was created inside Universidad de La Laguna.

Google Forms

Study Methodology

Reference Number

48

Year

2012

Authors

Oluigbo et al.

Correct Study? (check the reference number, the first number of the filename, against the reference number in AAP_refs.pdf)

- (●) Yes
- ( ) No

Classification Methods

- [x] MDTs/Experts
- [ ] Diagnostic code/medical records
- [ ] Predetermined Criteria
- [ ] Admission/Confession
- [ ] Conviction/Court Confirmation
- [ ] Witnessed
- [ ] Other:

Comment on Methodology

Describe how the study creates a sample of AHT cases

Series of children (up to 15years) who had decomprssive craniotomy. Abuse diagnosed by team with no stated criteria

Has the study adequately addressed the risk of incorporation bias and/or circular reasoning?

- [ ] Yes
- [x] No

This form was created inside Universidad de La Laguna.

Google Forms

Study Methodology

Reference Number

41

Year

2022

Authors

Kennedy et al.

Correct Study? (check the reference number, the first number of the filename, against the reference number in AAP_refs.pdf)

- (●) Yes
- ( ) No

Classification Methods

- [x] MDTs/Experts
- [x] Diagnostic code/medical records
- [ ] Predetermined Criteria
- [ ] Admission/Confession
- [ ] Conviction/Court Confirmation
- [ ] Witnessed
- [ ] Other:

Comment on Methodology

Describe how the study creates a sample of AHT cases

Children less than 6 years with acute head trauma.Abuse diagnosed by CAP team. No criteria stated

Has the study adequately addressed the risk of incorporation bias and/or circular reasoning?

- [ ] Yes
- [x] No

This form was created inside Universidad de La Laguna.

Google Forms

Study Methodology

Reference Number

90

Year

2013

Authors

Bradford et al.

Correct Study? (check the reference number, the first number of the filename, against the reference number in AAP_refs.pdf)

- (●) Yes
- ( ) No

Classification Methods

- [ ] MDTs/Experts
- [x] Diagnostic code/medical records
- [ ] Predetermined Criteria
- [ ] Admission/Confession
- [ ] Conviction/Court Confirmation
- [ ] Witnessed
- [ ] Other:

Comment on Methodology

Describe how the study creates a sample of AHT cases

Retrospective study of children under 24m with AHT in single medical centre. AHT is stated but no mantion of how it was diagnosed or criteria used.

Has the study adequately addressed the risk of incorporation bias and/or circular reasoning?

- [ ] Yes
- [x] No

This form was created inside Universidad de La Laguna.

Google Forms

Study Methodology

Reference Number

102

Year

2006

Authors

Tung et al.

Correct Study? (check the reference number, the first number of the filename, against the reference number in AAP_refs.pdf)

- (●) Yes
- ( ) No

Classification Methods

- [x] MDTs/Experts
- [x] Diagnostic code/medical records
- [ ] Predetermined Criteria
- [x] Admission/Confession
- [ ] Conviction/Court Confirmation
- [ ] Witnessed
- [ ] Other:

Comment on Methodology

Describe how the study creates a sample of AHT cases

CT scans in children <3 years with SDH. AHT diagnosed by CAP and multidisciplinary team with SDH and "injuries consistent with inflicted trauma".

Has the study adequately addressed the risk of incorporation bias and/or circular reasoning?

- [ ] Yes
- [x] No

This form was created inside Universidad de La Laguna.

Google Forms

Study Methodology

Reference Number

627

Year

1998

Authors

Gilles and Nelson

Correct Study? (check the reference number, the first number of the filename, against the reference number in AAP_refs.pdf)

- [x] Yes
- [ ] No

Classification Methods

- [ ] MDTs/Experts
- [ ] Diagnostic code/medical records
- [x] Predetermined Criteria
- [x] Admission/Confession
- [x] Conviction/Court Confirmation
- [x] Witnessed
- [ ] Other:

Comment on Methodology

Describe how the study creates a sample of AHT cases

Retrospective study of 14 infants who had head trauma and brain scans with follow up. All children had AHT but diagnostic criteria not stated.

Has the study adequately addressed the risk of incorporation bias and/or circular reasoning?

- [ ] Yes
- [x] No

This form was created inside Universidad de La Laguna.

Google Forms

Study Methodology

Reference Number

610

Year

1994

Authors

Budenz et al.

Correct Study? (check the reference number, the first number of the filename, against the reference number in AAP_refs.pdf)

- (●) Yes
- ( ) No

Classification Methods

- [x] MDTs/Experts
- [ ] Diagnostic code/medical records
- [ ] Predetermined Criteria
- [ ] Admission/Confession
- [ ] Conviction/Court Confirmation
- [ ] Witnessed
- [ ] Other:

Comment on Methodology

Describe how the study creates a sample of AHT cases

Autopsy series of infants whose injuries were attributed to abuse and "presumed violent shaking"

Has the study adequately addressed the risk of incorporation bias and/or circular reasoning?

- [ ] Yes
- [x] No

This form was created inside Universidad de La Laguna.

Google Forms

Study Methodology

Reference Number

520

Year

1999

Authors

Gleckman et al.

Correct Study? (check the reference number, the first number of the filename, against the reference number in AAP_refs.pdf)

- (●) Yes
- ( ) No

Classification Methods

- [x] MDTs/Experts
- [ ] Diagnostic code/medical records
- [ ] Predetermined Criteria
- [ ] Admission/Confession
- [ ] Conviction/Court Confirmation
- [ ] Witnessed
- [ ] Other:

Comment on Methodology

Describe how the study creates a sample of AHT cases

All SBS cases had a clinical history of whiplash shaking of the head and had autopsy find-
ings of subdural, subarachnoid, and bilateral retinal hemorrhages but did not have gross neuropathologic changes suggestive of DAI.

Has the study adequately addressed the risk of incorporation bias and/or circular reasoning?

- [ ] Yes
- [x] No

This form was created inside Universidad de La Laguna.

Google Forms

Study Methodology

Reference Number

481

Year

2018

Authors

Dingman et al.

Correct Study? (check the reference number, the first number of the filename, against the reference number in AAP_refs.pdf)

- (•) Yes
- ( ) No

Classification Methods

- [x] MDTs/Experts
- [x] Diagnostic code/medical records
- [ ] Predetermined Criteria
- [ ] Admission/Confession
- [ ] Conviction/Court Confirmation
- [ ] Witnessed
- [ ] Other:

Comment on Methodology

Describe how the study creates a sample of AHT cases

Chart revew of babies under 5 years with head trauma.AHT determine by CAP; no stated criteria

Has the study adequately addressed the risk of incorporation bias and/or circular reasoning?

- [ ] Yes
- [x] No

This form was created inside Universidad de La Laguna.

Google Forms

Study Methodology

Reference Number

225

Year

2000

Authors

DiScala et al.

Correct Study? (check the reference number, the first number of the filename, against the reference number in AAP_refs.pdf)

- (●) Yes
- ( ) No

Classification Methods

- [ ] MDTs/Experts
- [x] Diagnostic code/medical records
- [ ] Predetermined Criteria
- [ ] Admission/Confession
- [ ] Conviction/Court Confirmation
- [ ] Witnessed
- [ ] Other:

Comment on Methodology

Describe how the study creates a sample of AHT cases

Retrospective chart review of babies under 4 years.The distinction between child abuse and unintentional injury was made at the treating hospital and was not questioned. No criteria.

Has the study adequately addressed the risk of incorporation bias and/or circular reasoning?

- [ ] Yes
- [x] No

This form was created inside Universidad de La Laguna.

Google Forms

Study Methodology

Reference Number

173

Year

2013

Authors

Binenbaum et al.

Correct Study? (check the reference number, the first number of the filename, against the reference number in AAP_refs.pdf)

- (●) Yes
- ( ) No

Classification Methods

- [ ] MDTs/Experts
- [x] Diagnostic code/medical records
- [x] Predetermined Criteria
- [ ] Admission/Confession
- [ ] Conviction/Court Confirmation
- [ ] Witnessed
- [ ] Other:

Comment on Methodology

Describe how the study creates a sample of AHT cases

Consecutive study of children under 3 years.We rigorously applied a published and validated algorithm that excludes eye findings and HII to categorize a child as having had an inflicted injury.

Has the study adequately addressed the risk of incorporation bias and/or circular reasoning?

- [ ] Yes
- [x] No

This form was created inside Universidad de La Laguna.

Google Forms

Study Methodology

Reference Number

611

Year

2001

Authors

Marshall et al.

Correct Study? (check the reference number, the first number of the filename, against the reference number in AAP_refs.pdf)

- (●) Yes
- ( ) No

Classification Methods

- [x] MDTs/Experts
- [x] Diagnostic code/medical records
- [ ] Predetermined Criteria
- [ ] Admission/Confession
- [ ] Conviction/Court Confirmation
- [ ] Witnessed
- [ ] Other:

Comment on Methodology

Describe how the study creates a sample of AHT cases

Retrospective review of autopsy eye findings in babies with SBS diagnosed by general and ocular pathologists on the basis of SDH, RH and frequently occult bone fractures.

Has the study adequately addressed the risk of incorporation bias and/or circular reasoning?

- [ ] Yes
- [x] No

This form was created inside Universidad de La Laguna.

Google Forms

Study Methodology

Reference Number

40

Year

2010

Authors

Vinchon et al.

Correct Study? (check the reference number, the first number of the filename, against the reference number in AAP_refs.pdf)

- (●) Yes
- ( ) No

Classification Methods

- [ ] MDTs/Experts
- [ ] Diagnostic code/medical records
- [ ] Predetermined Criteria
- [x] Admission/Confession
- [ ] Conviction/Court Confirmation
- [ ] Witnessed
- [ ] Other:

Comment on Methodology

Describe how the study creates a sample of AHT cases

Corroborated Accidental Trauma was defined as “an accident having occurred in a public space in front of independent witnesses.”

Corroborated AHT was “abuse confessed by the perpetrator; information on the confession was obtained by a forensic pediatrician (MD) from judicial sources during expertise or after the judicial hearings were made public.”

The authors clarify that “in almost all cases, confession of abuse was obtained during the judicial process.” (Response to Lyn∅e: Questions about isolated trauma shaking and confessions, Vinchon, M. 2017, doi: 10.1007/s00381-017-3516-9).

A judicial process only starts after an investigation is begun, based on medical findings indicative of abuse. This selection bias means the article does not eliminate incorporation bias or circular reasoning. It was not stated how many cases involved child removal, but this is routine in France. No other circumstances of the confessions are provided, nor are interview transcripts, lengths of the investigations, possibilities of reunification with children, legal advice, likelihood of conviction, hopes for reduced sentences, or financial pressures.

One should also be concerned that “many cases are confessed as a single episode whereas evidence suggests repeated insults,” i.e., the confessions did not match the evidence—a red flag for false confessions.

Has the study adequately addressed the risk of incorporation bias and/or circular reasoning?

- [ ] Yes
- [x] No

This form was created inside Universidad de La Laguna.

Google Forms

Study Methodology

Reference Number

554

Year

2001

Authors

Maxeiner

Correct Study? (check the reference number, the first number of the filename, against the reference number in AAP_refs.pdf)

- (•) Yes
- ( ) No

Classification Methods

- [x] MDTs/Experts
- [ ] Diagnostic code/medical records
- [ ] Predetermined Criteria
- [ ] Admission/Confession
- [ ] Conviction/Court Confirmation
- [ ] Witnessed
- [ ] Other:

Comment on Methodology

Describe how the study creates a sample of AHT cases

This is a 2 case series. The medical evidence, including the SDHs, are interpreted by the "expert" authors as indicating acceleration/deceleration forces in both cases, and that the injuries were most likely inflicted. No independent evidence of abuse is provided and the caregivers denied abusing the infants.

Has the study adequately addressed the risk of incorporation bias and/or circular reasoning?

- [ ] Yes
- [x] No

This form was created inside Universidad de La Laguna.

Google Forms

Study Methodology

Reference Number

202

Year

1975

Authors

Tomasi and Rosman

Correct Study? (check the reference number, the first number of the filename, against the reference number in AAP_refs.pdf)

- (•) Yes
- ( ) No

Classification Methods

- [x] MDTs/Experts
- [ ] Diagnostic code/medical records
- [ ] Predetermined Criteria
- [ ] Admission/Confession
- [ ] Conviction/Court Confirmation
- [ ] Witnessed
- [ ] Other:

Comment on Methodology

Describe how the study creates a sample of AHT cases

This is a 2 case series of what was referred to as battered child syndrome, and involved ocular injuries.

The only information provided regarding the diagnosis was "The unusual histories, coupled with the multiple ecchymoses seen on both infants, led to a consideration of the battered child syndrome. This diagnosis was substantiated in both children after further investigation."

Has the study adequately addressed the risk of incorporation bias and/or circular reasoning?

- [ ] Yes
- [x] No

This form was created inside Universidad de La Laguna.

Google Forms

Study Methodology

Reference Number

97

Year

1997

Authors

Hymel et al.

Correct Study? (check the reference number, the first number of the filename, against the reference number in AAP_refs.pdf)

- (•) Yes
- ( ) No

Classification Methods

- [x] MDTs/Experts
- [ ] Diagnostic code/medical records
- [ ] Predetermined Criteria
- [ ] Admission/Confession
- [ ] Conviction/Court Confirmation
- [ ] Witnessed
- [ ] Other: ______

Comment on Methodology

Describe how the study creates a sample of AHT cases

"clear validation by a multidisciplinary child protection team that the head trauma had been inflicted".

This is clarified by "In this study, there was variability over the 13-year period in makeup of the multidisciplinary child protection team, expertise of radiographic reviewers, knowledge and application of emerging criteria for the diagnosis of child abuse, and cranial imaging technology, sensitivity, modality, and/or frequency. Accordingly, a conservative application of study inclusion criteria was deemed essential."

This seems to indicate that the authors used their own judgement as to which multidisciplinary child protection team validations to believe.

Has the study adequately addressed the risk of incorporation bias and/or circular reasoning?

- [ ] Yes
- [x] No

This form was created inside Universidad de La Laguna.

Google Forms

Study Methodology

Reference Number

216

Year

2009

Authors

Adamo et al.

Correct Study? (check the reference number, the first number of the filename, against the reference number in AAP_refs.pdf)

- (•) Yes
- ( ) No

Classification Methods

- [x] MDTs/Experts
- [x] Diagnostic code/medical records
- [ ] Predetermined Criteria
- [ ] Admission/Confession
- [ ] Conviction/Court Confirmation
- [ ] Witnessed
- [ ] Other:

Comment on Methodology

Describe how the study creates a sample of AHT cases

" a case was only classified as abuse or NAT after it was thoroughly investigated and proven as such by CPS."

" Patients with head injuries sustained from child abuse were identified using the International Classification of Diseases diagnosis codes."

Has the study adequately addressed the risk of incorporation bias and/or circular reasoning?

- [ ] Yes
- [x] No

This form was created inside Universidad de La Laguna.

Google Forms

Study Methodology

Reference Number

215

Year

2014

Authors

Roach et al.

Correct Study? (check the reference number, the first number of the filename, against the reference number in AAP_refs.pdf)

- (●) Yes
- ( ) No

Classification Methods

- [x] MDTs/Experts
- [x] Diagnostic code/medical records
- [ ] Predetermined Criteria
- [ ] Admission/Confession
- [ ] Conviction/Court Confirmation
- [ ] Witnessed
- [ ] Other:

Comment on Methodology

Describe how the study creates a sample of AHT cases

"queried the trauma database at Children's Hospital Colorado, a level I regional pediatric trauma center, over a 16-year period (January 1996–December 2011) for all children 5 years of age and younger with a diagnosis of traumatic brain injury (TBI)."

"Differentiation of AHT from accidental injury is determined by the Child Advocacy and Protection Team (CAP) at our institution. The CAP team is a multi-disciplinary team, which is asked to evaluate children if abuse is suspected by the healthcare providers. The CAP
team is made up of child abuse pediatricians, medical social workers, nurses, psychologists, and sociologist–attorney. When asked to consult on a patient, the CAP team is asked to make a determination as to the likelihood of abusive injury."

Has the study adequately addressed the risk of incorporation bias and/or circular reasoning?

- [ ] Yes
- [x] No

This form was created inside Universidad de La Laguna.

Google Forms

Study Methodology

Reference Number

112

Year

2015

Authors

Choudhary et al.

Correct Study? (check the reference number, the first number of the filename, against the reference number in AAP_refs.pdf)

- (selected) Yes
- ( ) No

Classification Methods

- [x] MDTs/Experts
- [x] Diagnostic code/medical records
- [ ] Predetermined Criteria
- [x] Admission/Confession
- [x] Conviction/Court Confirmation
- [ ] Witnessed
- [ ] Other:

Comment on Methodology

Describe how the study creates a sample of AHT cases

"Our institute maintains a child abuse registry, and the abusive head trauma cases were manually identified from that registry."

"Cases were identified as abusive head trauma if, after a diagnostic workup, there was consensus that the findings most likely represented abusive head trauma or there was a perpetrator confession or judicial ruling of abusive head trauma."

Has the study adequately addressed the risk of incorporation bias and/or circular reasoning?

- [ ] Yes
- [x] No

This form was created inside Universidad de La Laguna.

Google Forms

Study Methodology

Reference Number

362

Year

2019

Authors

Wright et al.

Correct Study? (check the reference number, the first number of the filename, against the reference number in AAP_refs.pdf)

- (●) Yes
- ( ) No

Classification Methods

- [x] MDTs/Experts
- [x] Diagnostic code/medical records
- [ ] Predetermined Criteria
- [ ] Admission/Confession
- [ ] Conviction/Court Confirmation
- [ ] Witnessed
- [ ] Other:

Comment on Methodology

Describe how the study creates a sample of AHT cases

"We identified children younger than 3 years with SDH caused by AHT from a previously initiated database of serial cases of AHT;"

Determination of AHT "was made by comparing the history with the injuries, including the constellation of extra-calvarial injuries... supplemented by results of routine inter-disciplinary conferences and protective services and police investigations. "

Has the study adequately addressed the risk of incorporation bias and/or circular reasoning?

- [ ] Yes
- [x] No

This form was created inside Universidad de La Laguna.

Google Forms

Study Methodology

Reference Number

148

Year

1991

Authors

Massicotte et al.

Correct Study? (check the reference number, the first number of the filename, against the reference number in AAP_refs.pdf)

- ○ Yes
- ○ No

Classification Methods

- [x] MDTs/Experts
- [ ] Diagnostic code/medical records
- [ ] Predetermined Criteria
- [x] Admission/Confession
- [ ] Conviction/Court Confirmation
- [ ] Witnessed
- [ ] Other:

Comment on Methodology

Describe how the study creates a sample of AHT cases

A 3 case series.
In one case "A complete forensic investigation determined that the child had been violently shaken."
In a second case "a 12-year-old babysitter .. stated that the infant rolled off the couch and landed on her milk bottle. When further questioned by the infant's mother, the babysitter stated that the infant may have slipped in the bathtub and hit her head. Still later, the caretaker mentioned shaking the infant and throwing her to the floor." This confession is from a 12 year old , and is made after medical evaluation identified findings assumed to be associated with abuse and after suspicion/accusations.
In the third case,
In the third, they state injuries including that "Contusions around these regions of the extremities are considered evidence of violent shaking." It is assessed as battered child syndrome.

Has the study adequately addressed the risk of incorporation bias and/or circular reasoning?

- [ ] Yes
- [x] No

This form was created inside Universidad de La Laguna.

Google Forms

Study Methodology

Reference Number

217

Year

2004

Authors

Ettaro et al.

Correct Study? (check the reference number, the first number of the filename, against the reference number in AAP_refs.pdf)

- (•) Yes
- ( ) No

Classification Methods

- [x] MDTs/Experts
- [x] Diagnostic code/medical records
- [x] Predetermined Criteria
- [ ] Admission/Confession
- [ ] Conviction/Court Confirmation
- [ ] Witnessed
- [ ] Other: ______

Comment on Methodology

Describe how the study creates a sample of AHT cases

Retrospective case note study children <3y. Abuse classified by one CAP on Duhaime criteria

Has the study adequately addressed the risk of incorporation bias and/or circular reasoning?

- [ ] Yes
- [x] No

This form was created inside Universidad de La Laguna.

Google Forms

Study Methodology

Reference Number

89

Year

2015

Authors

Kelly et al.

Correct Study? (check the reference number, the first number of the filename, against the reference number in AAP_refs.pdf)

- ○ Yes
- ○ No

Classification Methods

- [x] MDTs/Experts
- [ ] Diagnostic code/medical records
- [ ] Predetermined Criteria
- [ ] Admission/Confession
- [ ] Conviction/Court Confirmation
- [ ] Witnessed
- [ ] Other:

Comment on Methodology

Describe how the study creates a sample of AHT cases

"There is joint assessment by a consultant paediatrician and hospital social worker, meticulous re-taking of the history, detailed physical examination and additional investigations as indicated. Investigations are reviewed with appropriate subspecialists,
including radiology side-by-side with paediatric radiologists and neuroradiologists. Findings and differential diagnoses are discussed with the primary team. Further investigations and second opinions are sought where indicated, and all assessments receive weekly multidisciplinary peer review."

"AHT met these criteria: history of assault (and injuries compatible with that history) or injuries regarded as not compatible with the history provided by the end of the process described above. The diagnosis made in the final report was accepted for this review."

Has the study adequately addressed the risk of incorporation bias and/or circular reasoning?

- [ ] Yes
- [x] No

This form was created inside Universidad de La Laguna.

Google Forms

Study Methodology

Reference Number

384

Year

2007

Authors

Hymel et al.

Correct Study? (check the reference number, the first number of the filename, against the reference number in AAP_refs.pdf)

- (●) Yes
- ( ) No

Classification Methods

- [ ] MDTs/Experts
- [ ] Diagnostic code/medical records
- [ ] Predetermined Criteria
- [x] Admission/Confession
- [ ] Conviction/Court Confirmation
- [x] Witnessed
- [ ] Other: ____

Comment on Methodology

Describe how the study creates a sample of AHT cases

"Cases in which the child’s primary caregiver admitted abusive acts that could be linked to the child’s acute clinical presentation for traumatic cranial injuries

Cases in which an independent witness verified abusive acts that could be linked to the child’s acute clinical presentation for traumatic cranial injuries

Cases in which a child not yet cruising or walking became clearly and persistently ill with signs of acute cardiorespiratory compromise linked to his/her traumatic cranial injuries while in the care of a primary caregiver who denied any knowledge of a head injury
event

Cases in which the child’s primary caregiver provided an explanation for the child’s head injury event that was clearly developmentally inconsistent with the parent(s)’ description of their child’s developmental capabilities

Cases in which the child’s primary caregiver provided an explanation for the child’s head injury event that was highly inconsistent with repetition over time

Cases in which the head-injured child also revealed 2 noncranial injuries considered moderately or highly specific for abuse"

The reason caregivers were asked multiple times to provide an explanation was due to the existence of findings associated with AHT, so that started the process and is therefore incorporation bias. There is a strong risk that the parents' first explanation was rejected by the experts based on their preconceived ideas and also that their subsequent explanation was a genuine attempt to provide an explanation to findings that they were told were traumatic and inflicted.

If a caregiver denies trauma, the case is assumed to be AHT. That criterion is clealry incomplete in its description, as many cases where caregivers provide no history of trauma are not accused of AHT. The complete criteria involve findings associated with AHT, with the caregiver providing no history of trauma. Again, this has a high risk of incorporation bias.

Regarding the criteria of a caregivers explanation being developmentally inconsistent? Who judges that? Why is a caregiver's testimony a medical finding? What information was the caregiver reacting to? What findings lead to the explanation being asked for and why were the findings considered inconsistent with the explanation? Full details are required, including especially what findings lead to the investigation and what the caregiver was told and asked.

These categories of cases dominate the statistics.

Has the study adequately addressed the risk of incorporation bias and/or circular reasoning?

- [ ] Yes
- [x] No

This form was created inside Universidad de La Laguna.

Google Forms

Study Methodology

Reference Number

140

Year

2010

Authors

Muni et al.

Correct Study? (check the reference number, the first number of the filename, against the reference number in AAP_refs.pdf)

- (●) Yes
- ( ) No

Classification Methods

- [x] MDTs/Experts
- [ ] Diagnostic code/medical records
- [ ] Predetermined Criteria
- [ ] Admission/Confession
- [ ] Conviction/Court Confirmation
- [ ] Witnessed
- [ ] Other:

Comment on Methodology

Describe how the study creates a sample of AHT cases

Case series of infants with findings considered to be "consistent with SBS"

Has the study adequately addressed the risk of incorporation bias and/or circular reasoning?

- [ ] Yes
- [x] No

This form was created inside Universidad de La Laguna.

Google Forms

Study Methodology

Reference Number

347

Year

2016

Authors

Flom et al.

Correct Study? (check the reference number, the first number of the filename, against the reference number in AAP_refs.pdf)

- (●) Yes
- ( ) No

Classification Methods

- [x] MDTs/Experts
- [ ] Diagnostic code/medical records
- [ ] Predetermined Criteria
- [ ] Admission/Confession
- [ ] Conviction/Court Confirmation
- [ ] Witnessed
- [ ] Other:

Comment on Methodology

Describe how the study creates a sample of AHT cases

"The diagnosis of AHT in each case was made by the hospital-based Child Protection Team, a multi-disciplinary team that includes child abuse pediatricians."

Has the study adequately addressed the risk of incorporation bias and/or circular reasoning?

- [ ] Yes
- [x] No

This form was created inside Universidad de La Laguna.

Google Forms

Study Methodology

Reference Number

387

Year

2004

Authors

Keenan et al.

Correct Study? (check the reference number, the first number of the filename, against the reference number in AAP_refs.pdf)

- (●) Yes
- ( ) No

Classification Methods

- [x] MDTs/Experts
- [ ] Diagnostic code/medical records
- [ ] Predetermined Criteria
- [x] Admission/Confession
- [ ] Conviction/Court Confirmation
- [ ] Witnessed
- [ ] Other:

Comment on Methodology

Describe how the study creates a sample of AHT cases

"Inflicted TBI required evidence of TBI as defined above, accompanied by a confession documented in the medical record or a medical and child protective services determination that the injury was inflicted" When no social service or medical information was contained about injury cause in the medical record... two of the authors ("experts") "independently classified the case as inflicted or noninflicted."

Has the study adequately addressed the risk of incorporation bias and/or circular reasoning?

- [ ] Yes
- [x] No

This form was created inside Universidad de La Laguna.

Google Forms

Study Methodology

Reference Number

122

Year

2001

Authors

Geddes

Correct Study? (check the reference number, the first number of the filename, against the reference number in AAP_refs.pdf)

- (●) Yes
- ( ) No

Classification Methods

- [ ] MDTs/Experts
- [ ] Diagnostic code/medical records
- [x] Predetermined Criteria
- [x] Admission/Confession
- [x] Conviction/Court Confirmation
- [ ] Witnessed
- [ ] Other:

Comment on Methodology

Describe how the study creates a sample of AHT cases

"The diagnostic criteria used were: (i) head injuries in which there had been a confession by the perpetrator (n = 7); (ii) cases in which non-accidental head injury had been established as a result of conviction in a criminal court, and in which there were also unexplained extracranial injuries to support this (n = 19); (iii) cases with unexplained injuries elsewhere in the body, in addition to the head injury, but no conviction (n = 8); (iv) cases in which the carer was tried and convicted of injuring the child, but in which there were no extracranial injuries (n = 12); and (v) cases in which there was a major discrepancy between the explanation of the incident given by the carer and significant injuries such as a skull fracture, or if the history was developmentally incompatible (n = 7)."

Has the study adequately addressed the risk of incorporation bias and/or circular reasoning?

- [ ] Yes
- [x] No

This form was created inside Universidad de La Laguna.

Google Forms

Study Methodology

Reference Number

144

Year

1998

Authors

Mills

Correct Study? (check the reference number, the first number of the filename, against the reference number in AAP_refs.pdf)

- (x) Yes
- ( ) No

Classification Methods

- [x] MDTs/Experts
- [x] Diagnostic code/medical records
- [ ] Predetermined Criteria
- [ ] Admission/Confession
- [ ] Conviction/Court Confirmation
- [ ] Witnessed
- [ ] Other:

Comment on Methodology

Describe how the study creates a sample of AHT cases

All patients had a "diagnosis of traumatic retinal hemorrhage "
"all patients had undergone medial evaluation and had intracranial (subdural, subarachnoid, or intraparenchymal) hemorrhage on imaging studies (magnetic resonance imaging or computed tomography) as well as intraocular hemorrhage on ophthalmoscopy. Medical conditions causing bleeding were excluded by medical evaluation and systematic hematologic evaluation, when indicated. The history in each case was inconsistent with an etiology of accidental trauma. No patient had external evidence of significant direct head or eye trauma. Ophthalmic, radiologic, and neurologic evaluations were consistent with shaking as the mechanism of injury.3, 4 Patients varied from age 2 to 10 months at the time of diagnosis, with a mean age of 5 months. In addition to the medical evaluations, social work and police criminal investigations in each case concluded that the injury was not accidental.
"

Has the study adequately addressed the risk of incorporation bias and/or circular reasoning?

- [ ] Yes
- [x] No

This form was created inside Universidad de La Laguna.

Google Forms

Study Methodology

Reference Number

107

Year

2007

Authors

Ichord et al.

Correct Study? (check the reference number, the first number of the filename, against the reference number in AAP_refs.pdf)

- (●) Yes
- ( ) No

Classification Methods

- [ ] MDTs/Experts
- [x] Diagnostic code/medical records
- [x] Predetermined Criteria
- [ ] Admission/Confession
- [ ] Conviction/Court Confirmation
- [ ] Witnessed
- [ ] Other:

Comment on Methodology

Describe how the study creates a sample of AHT cases

AHT cases "identified from an institutional registry of children with inflicted trauma"

"Cases were classified as IT according to a previously published validated algorithm (Duhaime et al., 1992)."

Duhaime et al. 1992 algorithm has not been validated-

Has the study adequately addressed the risk of incorporation bias and/or circular reasoning?

- [ ] Yes
- [x] No

This form was created inside Universidad de La Laguna.

Google Forms

Study Methodology

Reference Number

123

Year

1995

Authors

Johnson et al.

Correct Study? (check the reference number, the first number of the filename, against the reference number in AAP_refs.pdf)

- (●) Yes
- ( ) No

Classification Methods

- [ ] MDTs/Experts
- [x] Diagnostic code/medical records
- [ ] Predetermined Criteria
- [ ] Admission/Confession
- [ ] Conviction/Court Confirmation
- [ ] Witnessed
- [ ] Other:

Comment on Methodology

Describe how the study creates a sample of AHT cases

"A retrospective review of medical records and radiologic imaging tudies was performed on 75 children admitted to the Children's lospital with a presumptive diagnosis of child abuse."

Has the study adequately addressed the risk of incorporation bias and/or circular reasoning?

- [ ] Yes
- [x] No

This form was created inside Universidad de La Laguna.

Google Forms

Study Methodology

Reference Number

128

Year

2012

Authors

Choudhary et al.

Correct Study? (check the reference number, the first number of the filename, against the reference number in AAP_refs.pdf)

- (●) Yes
- ( ) No

Classification Methods

- [x] MDTs/Experts
- [x] Diagnostic code/medical records
- [ ] Predetermined Criteria
- [ ] Admission/Confession
- [ ] Conviction/Court Confirmation
- [ ] Witnessed
- [ ] Other:

Comment on Methodology

Describe how the study creates a sample of AHT cases

Retrospective chart review of children <2 years. Cases selected from child abuse registry. No criteria stated

Has the study adequately addressed the risk of incorporation bias and/or circular reasoning?

- [ ] Yes
- [x] No

This form was created inside Universidad de La Laguna.

Google Forms

Study Methodology

Reference Number

367

Year

2005

Authors

Zimmerman et al.

Correct Study? (check the reference number, the first number of the filename, against the reference number in AAP_refs.pdf)

- (●) Yes
- ( ) No

Classification Methods

- [x] MDTs/Experts
- [ ] Diagnostic code/medical records
- [ ] Predetermined Criteria
- [ ] Admission/Confession
- [ ] Conviction/Court Confirmation
- [ ] Witnessed
- [ ] Other:

Comment on Methodology

Describe how the study creates a sample of AHT cases

Prospective study;Child Abuse Team, follow-up skeletal surveys were considered for all infants and toddlers who were suspected to be victims of physical abuse based upon their history, physical examination, initial skeletal survey and other available imaging studies such as head computed tomagraphy scans and radionucleotide bone scans. Information from the county children's services agency and law enforcement was considered as well.

Has the study adequately addressed the risk of incorporation bias and/or circular reasoning?

- [ ] Yes
- [x] No

This form was created inside Universidad de La Laguna.

Google Forms

Study Methodology

Reference Number

92

Year

2009

Authors

Matschke et al.

Correct Study? (check the reference number, the first number of the filename, against the reference number in AAP_refs.pdf)

- (●) Yes
- ( ) No

Classification Methods

- [x] MDTs/Experts
- [ ] Diagnostic code/medical records
- [x] Predetermined Criteria
- [x] Admission/Confession
- [x] Conviction/Court Confirmation
- [ ] Witnessed
- [ ] Other:

Comment on Methodology

Describe how the study creates a sample of AHT cases

"NAHI was identified on the basis of a confession by the perpetrator, a conviction for child abuse in a criminal court (with or without a confession), or a unanimous conclusion of all authors at a case conference, with the use of the following criteria for a positive decision: signs of serious external injury (hematomas or lacerations), unexplained fractures (long bones, ribs, or skull), SDB, traumatic intracerebral pathologic conditions (simple contusions or gliding contusions), retinal hemorrhage, and/or an alleged history overtly inadequate to the clinical picture. At least 3 criteria were needed for the diagnosis of NAHI in every case without a confession."

Has the study adequately addressed the risk of incorporation bias and/or circular reasoning?

- [ ] Yes
- [x] No

This form was created inside Universidad de La Laguna.

Google Forms

Study Methodology

Reference Number

598

Year

2008

Authors

Feldman et al.

Correct Study? (check the reference number, the first number of the filename, against the reference number in AAP_refs.pdf)

- (●) Yes
- ( ) No

Classification Methods

- [x] MDTs/Experts
- [ ] Diagnostic code/medical records
- [ ] Predetermined Criteria
- [x] Admission/Confession
- [ ] Conviction/Court Confirmation
- [ ] Witnessed
- [ ] Other:

Comment on Methodology

Describe how the study creates a sample of AHT cases

3 case series
Case 1: “the intracranial injury and multiple fractures led to a clear diagnosis of abusive injury. ”
Case 2: “her father pled guilty to her assault”
Case 3: “Her father subsequently admitted shaking her before the presenting event as well as grasping and jerking her leg during a prior event of frustration.”

Has the study adequately addressed the risk of incorporation bias and/or circular reasoning?

- [ ] Yes
- [x] No

This form was created inside Universidad de La Laguna.

Google Forms

Study Methodology

Reference Number

228

Year

1987

Authors

Meservey

Correct Study? (check the reference number, the first number of the filename, against the reference number in AAP_refs.pdf)

- (•) Yes
- ( ) No

Classification Methods

- [x] MDTs/Experts
- [ ] Diagnostic code/medical records
- [ ] Predetermined Criteria
- [ ] Admission/Confession
- [ ] Conviction/Court Confirmation
- [ ] Witnessed
- [ ] Other:

Comment on Methodology

Describe how the study creates a sample of AHT cases

“Suspected abuse cases were referred to the multidisaplinary child abuse team for assessment. The major criteria for referral included (1) inadequate, unlikely, or no explanation of the child’s injury, (2) history of injury inconsistent with the child’s development, (3) discrepancy in history given by caretakers or other family members, (4) multiple injuries or injuries of various ages, (5) past history of suspicious injuries or previous abuse reports, (6) delay in seeking medical treatment, (7) the child’s response to medical examination, since an abused or neglected child frequently accepts procedures in a passive manner, and (8) parent-child interactions suggesting lack of parental concern. “

“Children who did not meet the criteria established for identification of abuse were presumed to have sus-
tamed accidental injury. However, we have no absolute criteria for identification of accidental injury. It is possible, therefore, that cases of undetected abuse may have been included in the group of accidental injury cases.”

Some criteria were listed but they were described as “the major criteria included” so they are not strict predertermined criteria.

Extraordinary that they claim to have “no absolute criteria for identification of accidental injury. ” and think they have misclassified some abuse as accidents but do not consider at all they they may be misclassifying some accidents as abuse.

Has the study adequately addressed the risk of incorporation bias and/or circular reasoning?

- [ ] Yes
- [x] No

This form was created inside Universidad de La Laguna.

Google Forms

Study Methodology

Reference Number

129

Year

2014

Authors

Kadom et al.

Correct Study? (check the reference number, the first number of the filename, against the reference number in AAP_refs.pdf)

- (•) Yes
- ( ) No

Classification Methods

- [x] MDTs/Experts
- [ ] Diagnostic code/medical records
- [x] Predetermined Criteria
- [ ] Admission/Confession
- [ ] Conviction/Court Confirmation
- [ ] Witnessed
- [ ] Other:

Comment on Methodology

Describe how the study creates a sample of AHT cases

"A child abuse pediatrician with 8 years of experience (observer E) added the radiologic data to the pre-existing clinical, history and physical findings and used the information to assign children to one of four study outcome categories: presumptive, suspicious, accidental or undefined (modified Duhaime criteria)."

"Patient outcomes for statistical purposes of this study were defined based on modified Duhaime criteria [8]. The original Duhaime algorithm results in one of two clinical outcomes based on combining a child's injury type (skull fractures, intracranial hemorrhage, blunt craniofacial trauma) with a certain history or associated findings (unexplained long-bone fractures or old fractures; soft-tissue injury; no history of trauma; history of trivial or remote trauma, and changing history or developmentally incompatible history). The original Duhaime outcome is either"suspicious"or "presumptive" for inflicted head injury. We modified this classification scheme by adding two additional outcomes:"accidental" and "undefined." We used the study outcome"accidental" for children in whom we had complete information about injury type, history and associated findings, and who did not fulfill "presumptive" or "suspicious" Duhaime criteria. We used the study outcome"undefined" for children in whom we were missing information on injury type, history or physical examination findings. "

The Duhaime algorithm has never been validated. It selects for certain findings that are subjective.

Has the study adequately addressed the risk of incorporation bias and/or circular reasoning?

- [ ] Yes
- [x] No

This form was created inside Universidad de La Laguna.

Google Forms

Study Methodology

Reference Number

46

Year

2022

Authors

Hymel et al.

Correct Study? (check the reference number, the first number of the filename, against the reference number in AAP_refs.pdf)

- (●) Yes
- ( ) No

Classification Methods

- [x] MDTs/Experts
- [ ] Diagnostic code/medical records
- [ ] Predetermined Criteria
- [ ] Admission/Confession
- [ ] Conviction/Court Confirmation
- [ ] Witnessed
- [ ] Other:

Comment on Methodology

Describe how the study creates a sample of AHT cases

"All patients eligible for inclusion in the secondary analysis had previously been classified as having AHT, head trauma that was not abusive (non-AHT), or head trauma of indeterminate cause, by an expert medical panel."

Has the study adequately addressed the risk of incorporation bias and/or circular reasoning?

- [ ] Yes
- [x] No

This form was created inside Universidad de La Laguna.

Google Forms

Study Methodology

Reference Number

104

Year

1984

Authors

Merten et al.

Correct Study? (check the reference number, the first number of the filename, against the reference number in AAP_refs.pdf)

- (●) Yes
- ( ) No

Classification Methods

- [ ] MDTs/Experts
- [x] Diagnostic code/medical records
- [ ] Predetermined Criteria
- [ ] Admission/Confession
- [ ] Conviction/Court Confirmation
- [ ] Witnessed
- [ ] Other:

Comment on Methodology

Describe how the study creates a sample of AHT cases

Retrospective study reviewing medical records where AHT diagnosis was established by clinicians and the criteria used to identify AHT are not described in the study, but it is presumed to be based on MDTs/Experts. No control group: only those with a diagnosis of abusing injury were selected.

Has the study adequately addressed the risk of incorporation bias and/or circular reasoning?

- [ ] Yes
- [x] No

This form was created inside Universidad de La Laguna.

Google Forms

Study Methodology

Reference Number

146

Year

1989

Authors

Wilkinson et al.

Correct Study? (check the reference number, the first number of the filename, against the reference number in AAP_refs.pdf)

- (●) Yes
- ( ) No

Classification Methods

- [x] MDTs/Experts
- [ ] Diagnostic code/medical records
- [x] Predetermined Criteria
- [ ] Admission/Confession
- [ ] Conviction/Court Confirmation
- [ ] Witnessed
- [ ] Other:

Comment on Methodology

Describe how the study creates a sample of AHT cases

Prospective study of AHT/SBS determined based on findings of intraocular hemorrhage and intracranial injury in the absence of external signs of head trauma. No corroboration with history or scene. No control group.

Has the study adequately addressed the risk of incorporation bias and/or circular reasoning?

- [ ] Yes
- [x] No

This form was created inside Universidad de La Laguna.

Google Forms

Study Methodology

Reference Number

73

Year

2021

Authors

Pierce et al.

Correct Study? (check the reference number, the first number of the filename, against the reference number in AAP_refs.pdf)

- (selected) Yes
- No

Classification Methods

- [x] MDTs/Experts
- [ ] Diagnostic code/medical records
- [ ] Predetermined Criteria
- [ ] Admission/Confession
- [ ] Conviction/Court Confirmation
- [ ] Witnessed
- [ ] Other:

Comment on Methodology

Describe how the study creates a sample of AHT cases

Prospective study of children <4years looking for bruising.Each patient's case information was classified as abuse, nonabuse, or indeterminate by an expert panel composed of pediatric emergency medicine and child abuse pediatrics physicians and a biomechanical engineer, all with expertise in pediatric injury. No criteria stated.
One other potential limitation involves the categorization of patients as abuse or nonabuse and the possibility of misclassification. The study used a 9-member panel composed of experts in child injury with extensive clinical or research expertise, because there is no true criterion standard for abuse categorization.

Has the study adequately addressed the risk of incorporation bias and/or circular reasoning?

- [ ] Yes
- [x] No

This form was created inside Universidad de La Laguna.

Google Forms

Study Methodology

Reference Number

353

Year

2004

Authors

Zouros et al.

Correct Study? (check the reference number, the first number of the filename, against the reference number in AAP_refs.pdf)

- (●) Yes
- ( ) No

Classification Methods

- [x] MDTs/Experts
- [ ] Diagnostic code/medical records
- [ ] Predetermined Criteria
- [ ] Admission/Confession
- [ ] Conviction/Court Confirmation
- [ ] Witnessed
- [ ] Other:

Comment on Methodology

Describe how the study creates a sample of AHT cases

5 infants with bilateral SDH. Classified by MDT as abuse. No criteria stated

Has the study adequately addressed the risk of incorporation bias and/or circular reasoning?

- [ ] Yes
- [x] No

This form was created inside Universidad de La Laguna.

Google Forms

Study Methodology

Reference Number

443

Year

1999

Authors

Rao et al.

Correct Study? (check the reference number, the first number of the filename, against the reference number in AAP_refs.pdf)

- (●) Yes
- ( ) No

Classification Methods

- [x] MDTs/Experts
- [x] Diagnostic code/medical records
- [ ] Predetermined Criteria
- [ ] Admission/Confession
- [ ] Conviction/Court Confirmation
- [ ] Witnessed
- [ ] Other:

Comment on Methodology

Describe how the study creates a sample of AHT cases

Retrospective review of children <15y wih brain injury.The diagnosis of NAI in this group was made based on the combination of clinical and radiological findings and the exclusion of identifiable cause for the hypoxic ischemic injury and bleeding.

Has the study adequately addressed the risk of incorporation bias and/or circular reasoning?

- [ ] Yes
- [x] No

This form was created inside Universidad de La Laguna.

Google Forms

Study Methodology

Reference Number

94

Year

2001

Authors

Feldman et al.

Correct Study? (check the reference number, the first number of the filename, against the reference number in AAP_refs.pdf)

- (•) Yes
- ( ) No

Classification Methods

- [x] MDTs/Experts
- [ ] Diagnostic code/medical records
- [x] Predetermined Criteria
- [x] Admission/Confession
- [ ] Conviction/Court Confirmation
- [x] Witnessed
- [ ] Other:

Comment on Methodology

Describe how the study creates a sample of AHT cases

Mixture of prospective and retrospective review, where AHT was graded by study researchers using a categorization scheme that was later collapsed to reach statistical significance.

Provided criteria but many were subjective

Definite abuse based on corroborated, witnessed or confessed with no further information on how this was elicited or multiple trauma incompatible with given history with no information about how incompatibility was assessed. Researchers said they excluded retinal hemorrhages in their assessment and yet it is listed as a criterion in their assessment.

Has the study adequately addressed the risk of incorporation bias and/or circular reasoning?

- [ ] Yes
- [x] No

This form was created inside Universidad de La Laguna.

Google Forms

Study Methodology

Reference Number

150

Year

2002

Authors

Morad et al.

Correct Study? (check the reference number, the first number of the filename, against the reference number in AAP_refs.pdf)

- (●) Yes
- ( ) No

Classification Methods

- [x] MDTs/Experts
- [ ] Diagnostic code/medical records
- [ ] Predetermined Criteria
- [ ] Admission/Confession
- [ ] Conviction/Court Confirmation
- [ ] Witnessed
- [ ] Other:

Comment on Methodology

Describe how the study creates a sample of AHT cases

Enrollment - at least 2 features from list - 1) abnormal findings on imaging (all have it), 2) skeletal injuries, 3) ocular injury, history of child abuse that included shaking with or without blunt head trauma or an inadequate history to explain the injuries. N.B. 40% have direct impact. Excluded children believed to have suffered from "other forms of child abuse or with history of shaking, but did not exhibit characteristic signs of SBS. No controls.

Has the study adequately addressed the risk of incorporation bias and/or circular reasoning?

- [ ] Yes
- [x] No

This form was created inside Universidad de La Laguna.

Google Forms

Study Methodology

Reference Number

630

Year

2000

Authors

Barlow and Minns

Correct Study? (check the reference number, the first number of the filename, against the reference number in AAP_refs.pdf)

- (●) Yes
- ( ) No

Classification Methods

- [x] MDTs/Experts
- [x] Diagnostic code/medical records
- [x] Predetermined Criteria
- [ ] Admission/Confession
- [ ] Conviction/Court Confirmation
- [ ] Witnessed
- [ ] Other:

Comment on Methodology

Describe how the study creates a sample of AHT cases

Prospective study of hospital records for cases of NAHI. Defined as an acute encephalopathy with subdural haemorrhages, cerebral oedema, retinal haemorrhages,
and fractures, occurring in the context of an inappropriate or inconsistent history, and commonly with additional evidence of other impact or malicious injuries. No information on how any children had which/any/all of these findings.

Has the study adequately addressed the risk of incorporation bias and/or circular reasoning?

- [ ] Yes
- [x] No

This form was created inside Universidad de La Laguna.

Google Forms

Study Methodology

Reference Number

608

Year

1994

Authors

Gilliland et al.

Correct Study? (check the reference number, the first number of the filename, against the reference number in AAP_refs.pdf)

- (•) Yes
- ( ) No

Classification Methods

- [x] MDTs/Experts
- [ ] Diagnostic code/medical records
- [x] Predetermined Criteria
- [x] Admission/Confession
- [ ] Conviction/Court Confirmation
- [x] Witnessed
- [ ] Other:

Comment on Methodology

Describe how the study creates a sample of AHT cases

Prospective study of child deaths.The diagnosis of child abuse was generally made by the constellation of findings of severe injuries with confessions, witnesses, or circumstances
which indicated that the child's death was caused by the intentional act of another person.In the absence of natural disease or adequate history of severe head injury, the presence of retinal and ocular hemorrhage is pathognomonic of child abuse.

Has the study adequately addressed the risk of incorporation bias and/or circular reasoning?

- [ ] Yes
- [x] No

This form was created inside Universidad de La Laguna.

Google Forms

Study Methodology

Reference Number

607

Year

2006

Authors

WygnanskiJaffe et al.

Correct Study? (check the reference number, the first number of the filename, against the reference number in AAP_refs.pdf)

- (•) Yes
- ( ) No

Classification Methods

- [x] MDTs/Experts
- [ ] Diagnostic code/medical records
- [x] Predetermined Criteria
- [ ] Admission/Confession
- [ ] Conviction/Court Confirmation
- [ ] Witnessed
- [ ] Other:

Comment on Methodology

Describe how the study creates a sample of AHT cases

All autopsies of children with accidental or suspected abuse. All autopsies by Charles Smith. (Later discredited) Only the cases performed by this pathologist were included in this study.
SBS diagnosed by : typical abnormal findings on neuroimaging, (2) typical skeletal injury, (3) retinal hemorrhages,(4) history of abusive shaking with or without blunt head trauma, or (5) an inadequate history to explain the observed injuries.

Has the study adequately addressed the risk of incorporation bias and/or circular reasoning?

- [ ] Yes
- [x] No

This form was created inside Universidad de La Laguna.

Google Forms

Study Methodology

Reference Number

606

Year

2017

Authors

Puanglumyai and Lekawanvijit

Correct Study? (check the reference number, the first number of the filename, against the reference number in AAP_refs.pdf)

- (●) Yes
- ( ) No

Classification Methods

- [x] MDTs/Experts
- [x] Diagnostic code/medical records
- [ ] Predetermined Criteria
- [ ] Admission/Confession
- [ ] Conviction/Court Confirmation
- [ ] Witnessed
- [ ] Other:

Comment on Methodology

Describe how the study creates a sample of AHT cases

Autopsy study of medicolegal cases <3 years. Abuse based on 2 of : intracranial hemorrhage, 2) retinal hemorrhage, 3) marked brain swelling on neuroimaging or at autopsy, 4) inadequate history to explain the degree of head injury, and 5) confessions/reliable
witnesses or child abuse being confirmed in legal proceedings.

Has the study adequately addressed the risk of incorporation bias and/or circular reasoning?

- [ ] Yes
- [x] No

This form was created inside Universidad de La Laguna.

Google Forms

Study Methodology

Reference Number

176

Year

2012

Authors

Minns et al.

Correct Study? (check the reference number, the first number of the filename, against the reference number in AAP_refs.pdf)

- (●) Yes
- ( ) No

Classification Methods

- [x] MDTs/Experts
- [ ] Diagnostic code/medical records
- [ ] Predetermined Criteria
- [ ] Admission/Confession
- [ ] Conviction/Court Confirmation
- [ ] Witnessed
- [ ] Other:

Comment on Methodology

Describe how the study creates a sample of AHT cases

Prospective retinal imaging study on children with encephalopathy. Abuse diagnosed by MDT "It is difficult to be certain that a diagnosis of ITBI would have been confirmed by the Child Protection Case Conference, and subsequent legal proceedings, if RHs were not taken into consideration." No criteria for diagnosis of abuse

Has the study adequately addressed the risk of incorporation bias and/or circular reasoning?

- [ ] Yes
- [x] No

This form was created inside Universidad de La Laguna.

Google Forms

Study Methodology

Reference Number

352

Year

2022

Authors

Karmazyn et al.

Correct Study? (check the reference number, the first number of the filename, against the reference number in AAP_refs.pdf)

- (●) Yes
- ( ) No

Classification Methods

- [x] MDTs/Experts
- [x] Diagnostic code/medical records
- [ ] Predetermined Criteria
- [ ] Admission/Confession
- [ ] Conviction/Court Confirmation
- [ ] Witnessed
- [ ] Other:

Comment on Methodology

Describe how the study creates a sample of AHT cases

Retrospective review based on medical records of children with trauma suspected for abuse where the diagnoses had been established by board-certified child abuse pediatricians on the basis of a detailed medical history; complete physical examination including a dilated eye examination by a pediatric ophthalmologist; laboratory values; and imaging studies for accidental trauma, nonaccidental trauma, and medical conditions.

Has the study adequately addressed the risk of incorporation bias and/or circular reasoning?

- [ ] Yes
- [x] No

This form was created inside Universidad de La Laguna.

Google Forms

Study Methodology

Reference Number

593

Year

1984

Authors

Calder et al.

Correct Study? (check the reference number, the first number of the filename, against the reference number in AAP_refs.pdf)

- (●) Yes
- ( ) No

Classification Methods

- [ ] MDTs/Experts
- [ ] Diagnostic code/medical records
- [ ] Predetermined Criteria
- [ ] Admission/Confession
- [ ] Conviction/Court Confirmation
- [ ] Witnessed
- [x] Other:

Trauma was “alleged” and brains referred from another institution. No corroborative methodology described.

Comment on Methodology

Describe how the study creates a sample of AHT cases

These were 12 brains referred from another institution for allegations of repeated trauma where the infants and children had died from trauma. No further information is given about the alleged mechanisms or circumstances.

Has the study adequately addressed the risk of incorporation bias and/or circular reasoning?

- [ ] Yes
- [x] No

This form was created inside Universidad de La Laguna.

Google Forms

Study Methodology

Reference Number

166

Year

2013

Authors

DeRidder et al.

Correct Study? (check the reference number, the first number of the filename, against the reference number in AAP_refs.pdf)

- (•) Yes
- ( ) No

Classification Methods

- [ ] MDTs/Experts
- [ ] Diagnostic code/medical records
- [ ] Predetermined Criteria
- [ ] Admission/Confession
- [ ] Conviction/Court Confirmation
- [ ] Witnessed
- [ ] Other:

Comment on Methodology

Describe how the study creates a sample of AHT cases

Retrospective chart review of infants with subconjunctival haemorrhages referred to CAPs. No criteria for diagnosis of abuse.

Has the study adequately addressed the risk of incorporation bias and/or circular reasoning?

- [ ] Yes
- [x] No

This form was created inside Universidad de La Laguna.

Google Forms

Study Methodology

Reference Number

615

Year

2010

Authors

Fanconi and Lips

Correct Study? (check the reference number, the first number of the filename, against the reference number in AAP_refs.pdf)

- (●) Yes
- ( ) No

Classification Methods

- [x] MDTs/Experts
- [x] Diagnostic code/medical records
- [ ] Predetermined Criteria
- [x] Admission/Confession
- [ ] Conviction/Court Confirmation
- [ ] Witnessed
- [ ] Other:

Comment on Methodology

Describe how the study creates a sample of AHT cases

Hospitals and forensic centers report cases of SBS/AHT prospectively based on their diagnostic criteria. Used to determine incidence in a particular country and not to question the validity of the diagnosis. Criteria include clinical symptoms, eye findings and radiological findings or confession of shaking.

Has the study adequately addressed the risk of incorporation bias and/or circular reasoning?

- [ ] Yes
- [x] No

This form was created inside Universidad de La Laguna.

Google Forms

Study Methodology

Reference Number

213

Year

2002

Authors

Hoskote et al.

Correct Study? (check the reference number, the first number of the filename, against the reference number in AAP_refs.pdf)

- (selected) Yes
- ( ) No

Classification Methods

- [ ] MDTs/Experts
- [ ] Diagnostic code/medical records
- [ ] Predetermined Criteria
- [ ] Admission/Confession
- [ ] Conviction/Court Confirmation
- [ ] Witnessed
- [ ] Other:

Comment on Methodology

Describe how the study creates a sample of AHT cases

retrospective case note review of children <16y with SDH. AHT based on full clinical, radiological and social assessment.The useful predictors for NAHI were age less than 16 weeks, inconsistent history, presence of retinal haemorrhages, positive skeletal survey and unexplained bruising The diagnosis of NAHI was considered definite when supported by confirmatory court proceedings.

Has the study adequately addressed the risk of incorporation bias and/or circular reasoning?

- [ ] Yes
- [x] No

This form was created inside Universidad de La Laguna.

Google Forms

Study Methodology

Reference Number

103

Year

1979

Authors

Zimmerman et al.

Correct Study? (check the reference number, the first number of the filename, against the reference number in AAP_refs.pdf)

- (selected) Yes
- ( ) No

Classification Methods

- [x] MDTs/Experts
- [ ] Diagnostic code/medical records
- [ ] Predetermined Criteria
- [ ] Admission/Confession
- [ ] Conviction/Court Confirmation
- [ ] Witnessed
- [ ] Other:

Comment on Methodology

Describe how the study creates a sample of AHT cases

CT study of abused children with acute head trauma. Diagnosis of abuse :admitted abuse; evidence of bruises, fractures, retinal hemorrhages, or repeated trauma; and the findings of social workers, psychologists, and court employees.

Has the study adequately addressed the risk of incorporation bias and/or circular reasoning?

- [ ] Yes
- [x] No

This form was created inside Universidad de La Laguna.

Google Forms
